# Non-Invasive Physical Plasma as an Oncological Therapy Option: Modulation of Cancer Cell Growth, Motility, and Metabolism Without Induction of Cancer Resistance Factors

**DOI:** 10.3390/cancers17213517

**Published:** 2025-10-31

**Authors:** Yanqing Wang, Benedikt Eggers, Alexander Abazid, Holger H. H. Erb, Matthias B. Stope

**Affiliations:** 1 Physical Plasma Medicine Laboratories, Department of Gynecology and Gynecological Oncology, University Hospital Bonn, Venusberg-Campus 1, 53127 Bonn, Germany; 2 Department of Oral, Maxillofacial and Plastic Surgery, University Hospital Bonn, Welschnonnenstrasse 17, 53111 Bonn, Germany; 3 Department of General, Visceral and Thorax Surgery, Bundeswehr Hospital Berlin, Scharnhorststrasse 13, 10115 Berlin, Germany; alexanderabazid@bundeswehr.org; 4 Department of Urology, Faculty of Medicine, University Hospital Carl Gustav Carus, Technische Universität Dresden, Fetscherstraße 74, 01307 Dresden, Germany

**Keywords:** non-invasive physical plasma, cold atmospheric plasma, plasma oncology, physical plasma medicine, reactive oxygen species, mitochondria membrane potential, heat shock protein, cytoskeleton, lactate dehydrogenase

## Abstract

**Background**: Physical plasma, the fourth state of matter formed through gas ionization, has shown promise in various clinical applications, including wound healing and antimicrobial therapy. Recently, Non-invasive physical plasma (NIPP) selectively disrupts tumor cell proliferation and metabolism without inducing cytoprotective stress responses, positioning it as a promising adjunct in oncological therapies, though its underlying mechanisms remain insufficiently understood. **Methods**: In this study, we investigated the effects of NIPP (Plasma Care device) on six tumor cell lines, ovarian (SKOV-3, OVCAR-3), prostate (LNCaP, PC-3), and breast (MCF-7, MDA-MB-231). Cell proliferation and migration were assessed using CASY analysis and scratch assays, while cytoskeletal integrity, heat shock protein (HSP) expression, and key metabolic indicators were evaluated through immunofluorescence, Western blotting, and biochemical assays. **Results**: NIPP treatment significantly inhibited tumor cell proliferation and migration, disrupted cytoskeletal organization, and altered metabolic activity in a time-dependent manner. These effects were associated with increased intracellular reactive oxygen species (ROS), decreased mitochondrial membrane potential (MMP), enhanced glycolysis, and elevated lactate production. Notably, despite cellular stress, neither HSP expression nor superoxide dismutase (SOD) activity showed significant changes, suggesting a lack of classical stress-response activation. **Conclusions**: Our findings indicate that NIPP selectively impairs tumor cell function by inducing oxidative stress and metabolic disruption, without triggering protective HSP-mediated resistance pathways commonly seen in radiotherapy and chemotherapy. These results highlight the therapeutic potential of NIPP, particularly via the Plasma Care device, as a novel anticancer strategy.

## Simple Summary

Non-invasive physical plasma (NIPP), delivered using the Plasma Care device, was tested on six types of cancer cells from ovarian, prostate, and breast tumors. The treatment reduced cell growth and movement, disrupted cell structure, and changed how the cells produced energy. It increased oxidative stress but did not activate typical protective responses like heat shock proteins. These results suggest that NIPP may be a promising new method to fight cancer by targeting tumor cells without triggering resistance mechanisms.

## 1. Introduction

Physical plasma is the fourth physical state of matter, distinct from solids, liquids, and gases [1,2]. It is characterized by numerous charged particles in the form of ions or electrons and maintains an overall electrical neutrality [3]. Over the past two decades, plasma medicine has increasingly garnered attention from researchers [4]. This emerging field combines plasma physics, life sciences, and clinical medicine, focusing on applications in disinfection, wound healing, and cancer treatment [5]. Plasma Care is a newly developed handheld, portable device that generates non-invasive physical plasma (NIPP). It utilizes the principle of dielectric barrier discharges, with atmospheric air as the working gas for plasma generation. The NIPP device Plasma Care produces various active substances, including atomic nitrogen, oxygen, hydroxyl radicals, superoxide, nitric oxide, and more [6,7,8,9]. These active substances effectively deactivate bacteria and accelerates the healing process [10]. Non-invasive physical plasma (NIPP) selectively disrupts tumor cell proliferation and metabolism without inducing cytoprotective stress responses, positioning it as a promising adjunct in oncological therapies. Plasma devices generate ROS that interact at the air–liquid interface to form various reactive species, including hydroxyl radicals (•OH), hydrogen peroxide (H_2_O_2_), superoxide anions (O_2_^−^), and singlet oxygen [11]. These species modulate critical oncogenic and stress-related signaling pathways, such as glycolysis, oxidative phosphorylation, MAPK, NF-κB, and p53, thereby influencing tumor cell metabolism and survival [12,13].

Additionally, ROS can oxidize thiol groups in actin and tubulin, leading to cytoskeletal disruption and impaired cell migration [14]. Tumor cells, characterized by elevated basal ROS levels; highly polarized mitochondrial membranes; and weakened antioxidant defenses, like decreased glutathione (GLT) and superoxide dismutase (SOD), are more susceptible to ROS-induced damage compared to normal cells [15,16]. Persistent ROS accumulation can also reprogram tumor cell energy metabolism from oxidative phosphorylation to aerobic glycolysis (Warburg effect), favoring lactate production even under normoxic conditions [17]. Lactate accumulation promotes tumor proliferation, immune evasion, and microenvironment acidification, facilitating angiogenesis and suppressing cytotoxic immune cell functions, thereby creating an immunosuppressive milieu [18]. NIPP has been shown to disrupt glycolytic metabolism, inhibit lactate production, and potentially reverse immunosuppression, enhancing antitumor immune responses [19,20].

Currently, there are only a limited number of studies focusing on tumor therapy or tumor cell destruction despite a growing trend in tumor-related research. For instance, Mateu-Sanz et al. [21] demonstrated that NIPP treated medium can augment the cytotoxicity of doxorubicin (DOX) against prostate cancer PC-3 cells, impeding their migration and clonogenic potential. Importantly, nonmalignant cells remained unaffected by the combination of NIPP treated medium plus DOX. Researchers have employed the Piezobrush PZ2, a handheld NIPP-generating device based on piezoelectric direct discharge technology, to treat HT29 colorectal cancer cells and A549 lung cancer cells [22]. The treatment hindered cell proliferation and migration while promoting apoptosis [23,24]. NIPP was demonstrated to increase the concentration of NO and ROS in HepG2 cells, triggering lipid peroxidation and effectively inducing cancer cell death [25]. In addition, NIPP induced ROS/RNS accumulation, impaired antioxidant defenses, and activated endoplasmic reticulum stress, thereby leading to apoptosis in HepG2 cells [26]. However, these studies have only unveiled a fraction of the mechanisms underlying NIPP effects. There are more mechanisms of NIPP yet to be explored. Moreover, different NIPP-producing devices can yield varying effects on tumor cells, and different types of tumor cells may respond differently to NIPP generated by the same device. Consequently, comprehensive research from various perspectives using different devices and cancer cells is essential to gather robust evidence and uncover the potential mechanisms of NIPP on tumor cells. This research can ultimately contribute to the development of innovative cancer therapy strategies.

In this study, we explored the effects of NIPP on various tumor cell lines, including ovarian (SKOV-3, OVCAR-3), prostate (LNCaP, PC-3), and breast (MCF-7, MDA-MB-231) cancer cells. We examined the impact on cell proliferation, migration, and cytoskeletal arrangement. We also investigated the expression levels of a series of heat shock proteins (HSPs) related to oxidative active substance stimulation. Additionally, we assessed mitochondrial damage and changes in glucose metabolism. Our findings reveal, for the first time, that NIPP can inhibit glucose metabolism in tumor cells and accelerate lactate production. These results provide robust evidence supporting the potential application of Plasma Care in tumor treatment.

## 2. Materials and Methods

### 2.1. Cell Lines

SKOV-3 (Cryovial: 300343, Cell Lines Service GmbH, Heidelberg, Germany), OVCAR-3 (Cryovial: 300307, Cell Lines Service GmbH, Heidelberg, Germany), LNCaP (Cryovial:300265, Cell Lines Service GmbH, Heidelberg, Germany), PC-3 (Cryovial: 300312, Cell Lines Service GmbH, Heidelberg, Germany), MCF-7 (Cryovial: 300273, Cell Lines Service GmbH, Heidelberg, Germany), and MDA-MB-231 (Cat. no.: EP-CL-0150, Elabscience Biotechnology Inc., Houston, TX, USA) cell lines were purchased, aliquoted, and stored frozen. LNCaP and PC-3 cells were cultured in RPMI 1640 medium (Cat. no.: P04-16500PAN-Biotech, Adenbach, Germany) with 10% fetal bovine serum (FBS, Cat. no.: P30-3033, PAN-Biotech, Adenbach, Germany) and 0.125% penicillin-streptomycin (Cat. no.: 06-07100, PAN-Biotech, Adenbach, Germany). SKOV-3 cells were cultured in DMEM/F12 medium (Cat. no.: P04-16500, PAN-Biotech, Adenbach, Germany) with 5% FBS and 0.12% penicillin-streptomycin. MCF-7 and MDA-MB-231 cells were cultured in DMEM/F12 medium with 10% FBS and 0.125% penicillin-streptomycin. OVCAR-3 cells were cultured in RPMI medium with 10% FBS, 0.5% insulin (Cat. no.:51500056, Thermo Fisher Scientific, Frankfurt, Germany), and 0.125% penicillin-streptomycin. All cells were cultured at 37 °C in an incubator containing 5% CO_2_.

### 2.2. Non-Invasive Physical Plasma Device

The NIPP device was procured from Terraplasma-Medical, Garching bei München, Germany. The core of Plasma Care device is the physical plasma source, which generates physical plasma through microdejections. It is equipped with ultra-thin ceramic plates and sensitive electrical contacts. NIPP is partially ionized. The unique advantage of these physical plasmas is their ability to persist at room temperature and atmospheric pressure. Plasma Care device utilizes patented thin-film technology, representing an advancement in surface micro-discharge technology. The Plasma Source Unit comprises a high-voltage grid electrode, a dielectric, and a grounded structured electrode. By applying a high voltage of 3.5 kV, micro-discharges with a few millimeters expand in the electrode structured in squares. These micro-discharges ionize the gas, generating NIPP (Source: https://www.terraplasma-medical.com/technology/, accessed on 23 October 2024).

### 2.3. NIPP Treatment

**Figure 1:**
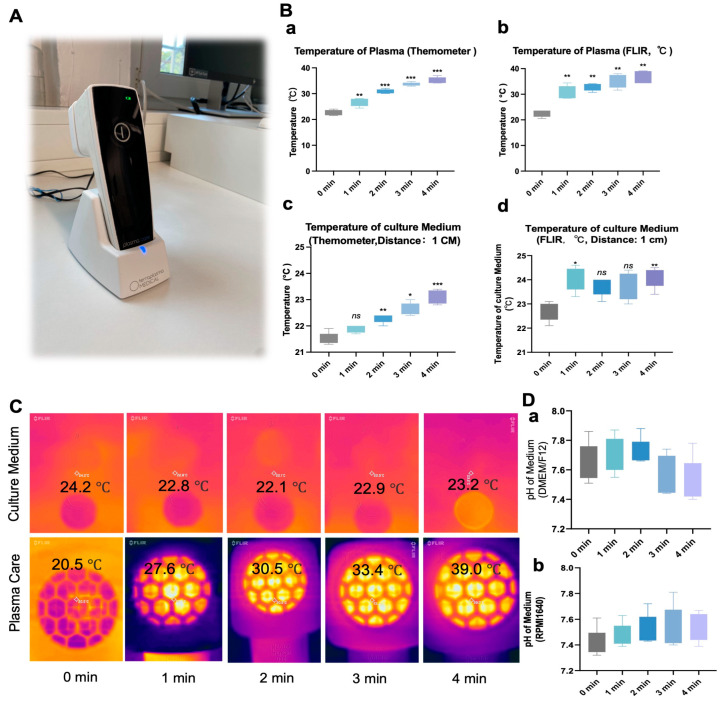
Temperature and pH in the cell culture medium remain stable after NIPP exposure. (**A**): The Plasma Care device; (**B**): Measurement of Plasma Care device temperature using both a thermometer (**a**) and FLIR infrared camera (**b**); Measurement of the cell culture medium temperature after Plasma Care treatment (1 min, 2 min, 3 min, 4 min) using a thermometer (**c**) and FLIR infrared camera (**d**); (**C**): FLIR infrared camera images of the cell culture medium and Plasma Care after treatment at various time intervals. (**D**): Temperature readings of the cell culture medium (**a**,**b**) after treatment with Plasma Care (1 min, 2 min, 3 min, 4 min); Each experiment was conducted at least three times. Each experimental group was compared to the control group. ***: *p* < 0.001, **: *p* < 0.01, *: *p* < 0.05.

NIPP is depicted as the charging device in Figure 1A. The operational parameters were as follows: Frequency Reception: 13.56 MHZ, voltages: 3–4 kV, Reception Bandwidth: 14 kHz, and Effective Radiant Power: 200 mW. The distance between the Plasma Care device and the culture plate ranged from 1 to 6 cm. The surface temperature during irradiation was monitored using an uncoupled infrared thermocouple. During treatment, cells were covered with 200 µL culture medium. After treatment, 2 mL was added to the culture plates.

### 2.4. pH and Temperature Measurement in Culture Media

Cell-free complete cell culture media (DMEM/F12 and RPMI 1640) underwent NIPP treatment for 1, 2, 3, and 4 min. Immediately following NIPP treatment, the pH and temperature of the culture media were measured using a pH meter (Benchtop pH Meters pH 50 VioLab Basic, Art. No. HAE2.1, Roth, Germany), an infrared thermometer (FLIR, USA), and a digital penetration thermometer (Cat. No.: 30.1033, TFA Dostmann, Germany), respectively.

### 2.5. Viable Cell Count Detection

CASY Cell Counter (ols-bio, Bremen, Germany) was utilized to determine the viable cell count for all cell lines. Adherent cells were detached by 0.1% trypsin and resuspended in the CASY ton solution. Measurements were performed using a capillary with a diameter of 150 µm and cell line-specific gate settings to differentiate between living cells, dead cells, and cellular debris. The number of living cells was determined in duplicate for each passage.

### 2.6. Wound-Healing Assay

Cells were seeded in 6-well culture plates. After cell overgrowth on the plate, a 200 µL pipette tip was used to create a straight line on the cell plate, which was then photographed. Following treatment with NIPP, the cells were cultured in the incubator for 24 h, and additional photographs were taken.

### 2.7. Fluorescence Microscopy

Cells were seeded on cover slides. At 24 h, 48 h, and 72 h after NIPP treatment, all cells were fixed using 4% paraformaldehyde for 15 min. Cell membrane permeabilization was achieved using 0.05% Triton X-100 (Ferak, Berlin, Germany) for 5 min. Visualization of the actin cytoskeleton was accomplished using a green fluorescent phalloidin conjugate (Cat. No.: 00042, Biotium CF™488A, Sunnyvale, CA, USA) at a concentration of 2 µg/mL in PBS, while a blue fluorescent DAPI (1 µg/mL, Cat. No.: AG-CR1-3668-M005, AdipoGen Life Sciences, Epalinges, Switzerland) was employed to visualize the nucleus. Images were captured using a fluorescent phase-contrast microscope (ZOETM Fluorescent Cell Imager, BIORAD, San Diego, CA, USA) at 20× magnification.

### 2.8. Western Blot Analysis

Cells were lysed in Laemmli buffer and heated at 95 °C for 5 min. Proteins were separated by electrophoresis on a 10% SDS-PAGE gel and transferred to a PVDF membrane for 30 min. Membranes were blocked with 5% non-fat milk in TBST for 1 h at room temperature. Primary antibodies were incubated overnight at 4 °C. After three washes with TBST, membranes were incubated with secondary antibodies for 1 h at room temperature. Following another three TBST washes, signals were detected using ECL reagent. A 100x Protease inhibitor Cocktail (Cat. No.: #5871, Cell Signaling Technology, Frankfurt, Germany) was used. In the current study, the primary antibodies employed were as follows: Anti-HSP27 (Mouse, 1:10,000, Cat. No.: #2402, Cell Signaling Technology, Frankfurt, Germany); Anti-HSP40 (Rabbit, 1:10,000, Cat. No.: #4871, Cell Signaling Technology, Frankfurt, Germany); Anti-HSP70 (Rat, 1:10,000, Cat. No.: #4873, Cell Signaling Technology, Frankfurt, Germany; Anti-HSP90a (Rabbit, 1:10,000, Cat. No.: #8165, Cell Signaling Technology, Frankfurt, Germany); Anti-HSP90b (Rabbit, 1:10,000, Cat. No.: #5087, Cell Signaling Technology, Frankfurt, Germany); Anti-GAPDH (Rabbit, 1:10,000, Cat. No.: #2118, Cell Signaling Technology, Frankfurt, Germany). In this study, secondary antibodies included: Anti-rabbit (1:10,000, Cat. No.: #7074, Cell Signaling Technology, Frankfurt, Germany); Anti-rat (1:10,000, Cat. No.: #7077, Cell Signaling Technology, Frankfurt, Germany); Anti-Mouse (1:10,000, Cat. No.: #7076, Cell Signaling Technology, Frankfurt, Germany). Both GAPDH and target protein bands were visualized on the same membrane. The Western blot bands were scanned using an Azure Biosystems instrument (Biozym, Hamburg, Germany). ECL Western Blotting Substrate (Cat. No.: 34075, Thermo Fisher Scientific, Frankfurt, Germany) was used.

### 2.9. Evaluation of Mitochondria Membrane Potential

The Mitochondria Membrane Potential Kit was procured from Sigma-Aldrich (Cat. No.: MAK159, Sigma-Aldrich, Merck KGaA 126, Darmstadt, Germany). This kit utilizes JC-10, a superior alternative to JC-1, to assess the loss of Mitochondrial membrane potential (MMP) in cells. The microplate reader (Infinite^®^ 200 PRO, TECAN, Männedorf, Switzerland) was employed with a fluorometric method to detect MMP. Cells (3000/well) were seeded in 96-well plates overnight. The experiment followed the instructions in the kit. Monitor the fluorescence intensity (Green fluorescent λex = 490/λem = 525 nm) and (Red fluorescent λex = 540/λem = 590 nm) for ratio analysis. The red/green fluorescence intensity ratio was used to determine MMP. All values were subsequently normalized to the control group. The experiment was performed with six replicates.

### 2.10. Evaluation of Glucose Level

The EnzytecTM Liquid D-Glucose kit was obtained from Sigma-Aldrich (Art. No.: E8140, R-Biopharm AG, Darmstadt, Germany). The microplate reader (Infinite^®^ 200 PRO, TECAN, Männedorf, Switzerland) was employed using a colorimetric method to detect the OD value of glucose. The experiment followed the kit’s instructions, and the calculation of glucose levels was carried out per the formula provided in the instructions. All values are relative to each cell and were normalized to the control group. Each experiment was repeated three times.

### 2.11. Evaluation of H_2_O_2,_ NO_2_^−^ and NO_3_^−^ Level in Medium

The Amplex™ Red Hydrogen Peroxide/Peroxidase Assay Kit was purchased from Invitrogen (Cat. No. A22188, Invitrogen, Carlsbad, CA, USA). A microplate reader with a fluorometric method was used to detect H_2_O_2_ levels. The experiment followed the instructions in the kit. All values are relative to each cell and were normalized to the control group. The experiment had six replicates. A Quantofix Relax reflectance photometer (Macherey-Nagel, Düren, Germany) with Quantofix nitrite (NO_3_^−^ measuring range 0.0–80.0 mg/L; Macherey-Nagel, Düren, Germany), and Quantofix nitrate 100 (NO_3_^−^; measuring range 0.0–100.0 mg/L; Macherey-Nagel, Düren, Germany) was used to determine selected ROS.

### 2.12. Evaluation of ROS Level Intracellular

Fluorometric Intracellular ROS Kit was purchased from Sigma-Aldrich (Cat. No.: MAK145, Sigma-Aldrich, Merck KGaA 126, Darmstadt, Germany). A microplate reader was used with a colorimetric method to detect the OD value of ROS. The experiment followed the instructions in the kit. Measure the fluorescence intensity at lex = 490/lem = 525 nm. All values are relative to each cell and were normalized to the control group. Each experiment was repeated three times.

### 2.13. Evaluation of Lactate Level

The Lactate Kit was procured from Sigma-Aldrich (Cat. No.: MAK064, Sigma-Aldrich, Merck KGaA 126, Darmstadt, Germany). A microplate reader was used with a colorimetric method to detect the OD value of lactate. The experiment followed the instructions in the kit, and the calculation of lactate levels was carried out according to the formula in the instructions. All values are relative to each cell and were normalized to the control group. Each experiment was repeated three times.

### 2.14. Evaluation of Intracellular Lactate Dehydrogenase (LDH) Level

The Cytotoxicity Detection Kit PLUS (LDH) kit was obtained from Roche (Cat. No. 04744926001, Darmstadt, Germany). The microplate reader was used to detect the absorbance at 490 nm. The experiment followed the instructions in the kit. The assay demonstrates a linear relationship between cell number and LDH signal intensity. All values are relative to each cell and were normalized to the control group. The experiment had six replicates.

### 2.15. Evaluation of Intracellular Superoxide Dismutase Activity

SOD catalyzes the dismutation of the superoxide anion (O_2_^−^) into hydrogen peroxide and molecular oxygen, making it one of the most crucial antioxidative enzymes. The SOD Assay Kit was obtained from Sigma-Aldrich (Cat. No.: 19160, Sigma-Aldrich, Merck KGaA 126, Darmstadt, Germany). The indirect method using nitroblue tetrazolium was employed in this experiment. The experiment followed the instructions in the kit. All values were finally normalized to the control group, and the experiment had six replicates.

### 2.16. Statistical Analysis

Data are presented as the mean ± SEM to indicate the precision of the estimated mean. For comparisons among multiple groups, statistical significance was determined using one-way analysis of variance (ANOVA) followed by Dunnett’s post hoc test for comparison against a single control group. All analyses were performed using GraphPad Prism 8 (GraphPad Software, Boston, MA, USA). Significance levels are denoted as follows: * *p* < 0.05, ** *p* < 0.01, and *** *p* < 0.001.

## 3. Results

### 3.1. NIPP Did Not Significantly Alter the Culture Medium Temperature and pH

Given that NIPP is a high-energy state of matter typically produced under conditions of high temperature, high pressure, or high-frequency electric fields, we first investigated the impact of a NIPP-generating device on cell culture medium at room temperature. Figure 1A is the NIPP device. Our results (Figure 1B(a,b)) revealed that the temperature of plasma care device increased from 24 °C to 38 °C when operated for 4 min operation. We also assessed the temperature of two types of media used in our cell culture experiments. Despite a statistically significant *p*-value for groups with varying treatment durations compared to the control group, the maximum temperature between the two media was approximately 25 °C, which is well below physiological body temperature (Figure 1B(c,d)). As shown in Figure 1C, the cell culture medium exhibited only a minimal temperature increase after treatment with Plasma Care, despite a significant temperature rise in the Plasma Care device itself. These findings suggest that NIPP treatment does not cause thermal damage, such as burns or scalding, to human cells or tissues. NIPP contains of reactive species, including atomic nitrogen, atomic oxygen, hydroxyl radicals, superoxide, and nitric oxide. These reactive components can interact with the cell culture medium, potentially generating acidic compounds that may alter pH, a critical factor for cell survival. Therefore, we measured the pH of the medium after NIPP treatment. Regarding the pH effect (Figure 1D(a,b)), only minimal changes were observed, none of which reached statistical significance.

### 3.2. NIPP-Induced Accumulation of Hydrogen Peroxide (H_2_O_2_), Nitrite (NO_2_^−^) and Nitrate (NO_3_^−^) in the Medium

**Figure 2:**
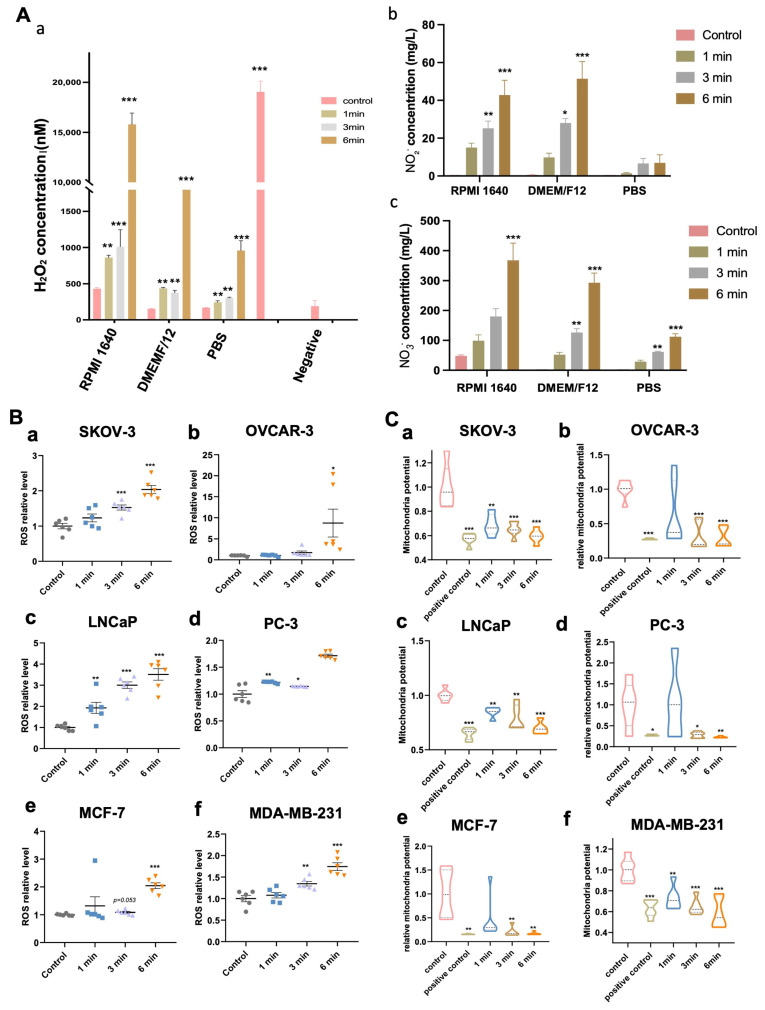
NIPP-induced alterations in extracellular H_2_O_2_ levels, intracellular ROS production, and MMP in tumor cells. (**A**): H_2_O_2_ level in the cell culture medium RPMI 1640 and DMEM/F12, and PBS after treatment with NIPP (**a**); NO_2_^−^ level in the cell culture medium RPMI 1640 and DMEM/F12, and PBS after treatment with NIPP (**b**); NO_3_^−^ level in the cell culture medium RPMI 1640 and DMEM/F12, and PBS after treatment with NIPP (**c**). (**B**): ROS level inside the tumor cells SKOV-3 (**a**), OVCAR-3 (**b**), LNCaP (**c**), PC-3 (**d**), MCF-7 (**e**), MDA-MB-231 (**f**) cells, after treatment with the NIPP in 30 min (*n* = 6). All data are normalized to the control group; (**C**): Mitochondria membrane potential relative level in the medium of SKOV-3 (**a**), OVCAR-3 (**b**), LNCaP (**c**), PC-3 (**d**), MCF-7 (**e**), MDA-MB-231 (**f**) cells after the exposure of NIPP 24 h, 48 h, and 72 h. Every experiment was replicated at least 3 times. All data are normalized to the control group. *** *p* < 0.001, **: *p* < 0.01, *: *p* < 0.05.

NIPP-generated ROS can be produced through interactions with both air and liquid phases. Oxygen-based reactive species include hydroxyl radicals (-OH), hydrogen peroxide (H_2_O_2_), superoxide (O_2_*^−^*), singlet oxygen (1O_2_), ozone (O_3_), nitrogen oxide (NO), nitrogen dioxide (NO_2_), nitrogen tetroxide (N_2_O_4_), nitrogen trioxide (NO_3_), nitrous oxide (N_2_O), and peroxynitrite (ONOO-) [27,28]. Hydroxyl radicals and nitric oxide are primarily formed through NIPP–air interactions, while more stable species such as nitrites, nitrates, and H_2_O_2_ are predominantly generated via NIPP–liquid interactions. Bekeschus et al. demonstrated that while H_2_O_2_ is a dominant contributor to cellular oxidation induced by NIPP, it is not the sole factor [27]. In our results (Figure 2A), we observed a significant increase in H_2_O_2_, Nitrite (NO_2_^−^) and Nitrate (NO_3_^−^) levels in the RPMI 1640 and DMEM/F12 cell culture medium. Furthermore, H_2_O_2_, NO_2_^−^ and NO_3_^−^ concentration increased proportionally with longer NIPP exposure times. In contrast, no detectable accumulation of H_2_O_2_, NO_2_^−^ and NO_3_^−^ was observed in PBS, suggesting that the oxidative byproducts are largely derived from the nutritional components of the culture media. These findings indicate that plasma treatment induces oxidative stress primarily through interactions with media constituents, rather than through water or buffer systems alone.

### 3.3. NIPP-Induced Accumulation of Intracellular ROS of Tumor Cells

As previously mentioned, NIPP enable to generate a substantial quantity of ROS, which can penetrate cells though the cell membrane, and initiate a series of reactions. To investigate the changes in intracellular ROS levels following varying durations of NIPP exposure, we assessed ROS levels within the cells. As shown in Figure 2B, average intracellular ROS levels in each cell line exhibited an increasing trend with longer NIPP treatment durations, suggesting that ROS likely plays a significant role in mediating subsequent cellular responses.

### 3.4. NIPP-Induced Mitochondria Membrane Potential Decrease

Since NIPP generates substantial amounts of ROS that can penetrate tumor cells, it is essential to note that the intracellular ROS accumulation can lead to mitochondrial damage, characterized by a reduction in mitochondrial membrane potential (MMP) and the release of cytochrome c [29]. To assess mitochondrial damage, we investigated whether ROS accumulation induced by NIPP correlates with a decrease in MMP in tumor cells. Our experimental results supported this hypothesis, demonstrating a significant reduction in MMP with increasing durations of NIPP treatment (Figure 2C).

### 3.5. NIPP Attenuates Tumor Cell Growth

**Figure 3:**
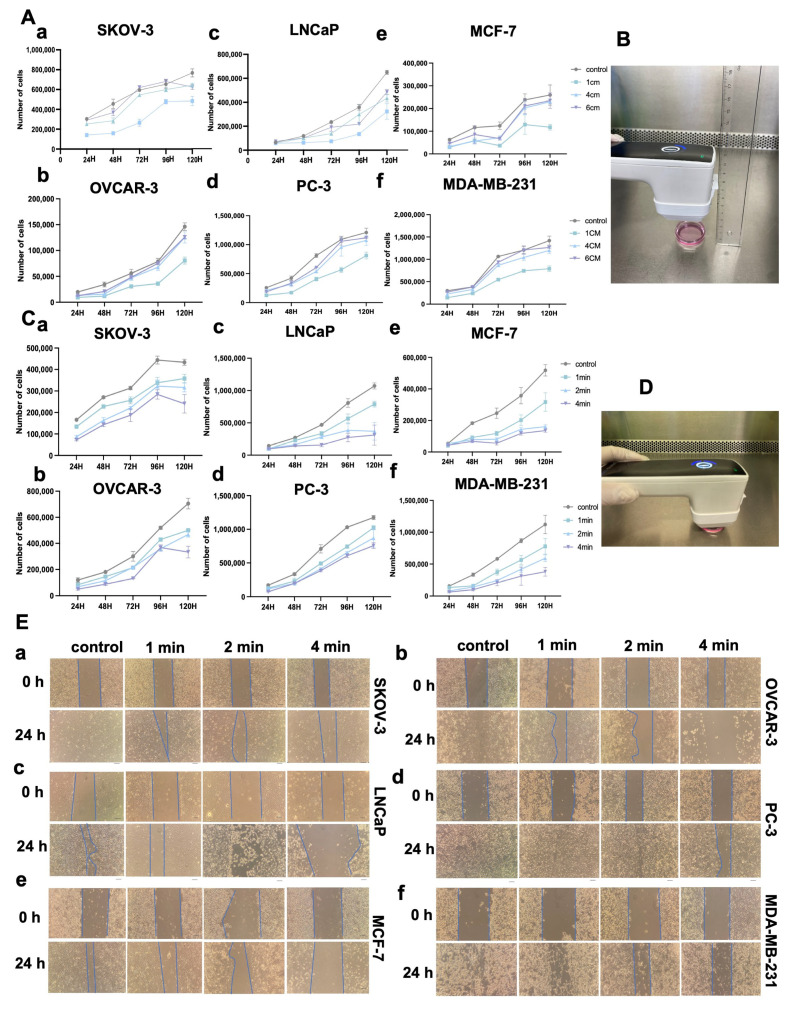
NIPP inhibits the proliferation of tumor cells. (**A**): Cell growth curves for SKOV-3 (**a**), OVCAR-3 (**b**), LNCaP (**c**), PC-3 (**d**), MCF-7 (**e**), and MDA-MB-231 (**f**) cells. The groups were categorized based on their distance from NIPP to the medium surface: the control group, 1 cm group, 3 cm group, and 6 cm group. All groups, except the control group, were exposed to NIPP for 4 min. Each experiment was conducted at least three times. (**B**): Visualization of NIPP treatment tumor cells with different distances. (**C**): Cell growth curves for SKOV-3 (**a**), OVCAR-3 (**b**), LNCaP (**c**), PC-3 (**d**), MCF-7 (**e**), and MDA-MB-231 (**f**) cells. The groups were organized based on the treatment time of NIPP for the tumor cells: the control group, 1 min group, 2 min group, and 4 min group. Except for the control group, the distance of NIPP from the culture medium was 1 cm for all groups. (**D**): Visualization of NIPP treatment tumor cells with different duration. Each experiment was repeated at least three times. (**E**): Wound-healing Test of SKOV-3 (**a**), OVCAR-3 (**b**), LNCaP (**c**), PC-3 (**d**), MCF-7 (**e**), and MDA-MB-231 (**f**) cells at 0 and 24 h. Each experimental group was compared with the control group.

To assess the impact of NIPP on tumor cells, we treated SKOV-3, OVCAR-3, LNCaP, PC-3, MCF-7, and MDA-MB-231 cell lines using a NIPP device Plasma Care (Terraplasma Medical, Garching bei München, Germany). The distance between the NIPP device and the surface of the cell culture medium was set at 1 cm, 4 cm, and 6 cm. As shown in Figure 3A,B, all experimental groups were exposed to NIPP for 4 min, except for the untreated control group. Notably, as the distance between the NIPP device and the culture medium increased, the inhibitory effect of NIPP on tumor cell proliferation progressively diminished. As illustrated in Figure 3C,D, NIPP generally exhibited an inhibitory effect on tumor cell proliferation, and this effect became more pronounced with longer treatment duration.

### 3.6. NIPP Affects Tumor Cells’ Migration Ability and Cell Morphology

**Figure 4:**
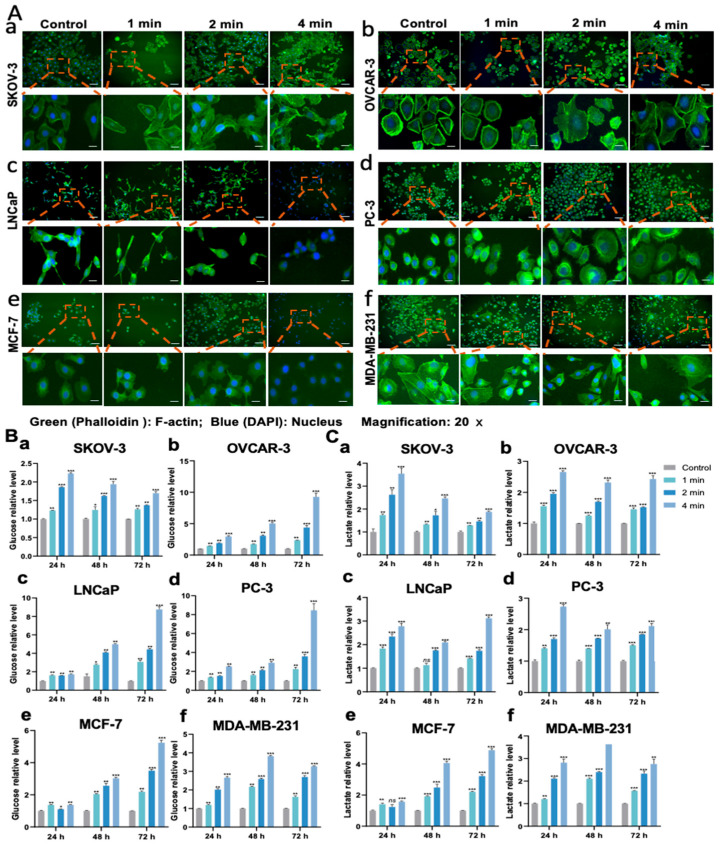
NIPP treatment alters the cytoskeletal organization of tumor cells and glucose consumption in tumor cell culture medium. (**A**): Immunofluorescence of SKOV-3 (**a**), OVCAR-3 (**b**), LNCaP (**c**), PC-3 (**d**), MCF-7 (**e**), and MDA-MB-231 (**f**) cells. Green represents F-actin, and Blue represents the Nucleus. Magnification 20×. Next row of pictures are partial screenshots from the previous row. (**B**): Glucose relative level in the medium of SKOV-3 (**a**), OVCAR-3 (**b**), LNCaP (**c**), PC-3 (**d**), MCF-7 (**e**), and MDA-MB-231 (**f**) cells after the exposure to NIPP for 24 h, 48 h, and 72 h. (**C**): Lactate relative level in the medium of SKOV-3 (**a**), OVCAR-3 (**b**), LNCaP (**c**), PC-3 (**d**), MCF-7 (**e**), and MDA-MB-231 (**f**) cells after the exposure to NIPP for 24 h, 48 h, and 72 h. Every experiment was replicated at least 3 times. Each experimental group was compared with the control group. ***: *p* < 0.001, **: *p* < 0.01, *: *p* < 0.05.

To investigate the impact of NIPP on tumor cell migration, we performed a wound-healing assay. As shown in Figure 3E, compared with the control group, the migration distance of tumor cells was significantly reduced, particularly in the group treated with NIPP for 4 min. These results demonstrate that NIPP exerts a time-dependent inhibitory effect on tumor cell motility. Furthermore, after 24 h of NIPP exposure, we observed a marked reduction in cell proliferation and viability. Many cells exhibited floating and clumping behavior, especially in the 4 min treatment group. To assess morphological and cytoskeletal changes, we performed immunofluorescence staining to examine the expression and localization of F-actin. As shown in Figure 4A, immunofluorescence analysis 24 h post-NIPP treatment revealed clear cytoskeletal disorganization compared to untreated cells. This effect was particularly evident in OVCAR-3 cells (Figure 4A(b)). where F-actin staining, which was concentrated along the cell membrane in the control group, became increasingly disordered with longer treatment durations. In LNCaP cells, F-actin staining was barely detectable in the 4 min treatment group, and cell morphology was no longer clearly visible (Figure 4A(c)). In PC-3 cells, the average cell diameter appeared to increase following NIPP exposure (Figure 4A(d)). In MCF-7 cells, similar to LNCaP, cytoskeletal staining was nearly absent and cell morphology was indistinct in the 4 min treatment group (Figure 4A(e)). In contrast, MDA-MB-231 cells exhibited less pronounced cytoskeletal alterations compared to the other cell lines (Figure 4A(f)). In summary, NIPP significantly disrupts the cytoskeletal structure of tumor cells, which plays a crucial role in cell motility, migration, and the maintenance of normal cellular morphology.

### 3.7. NIPP-Induced Mass Glucose in the Medium

Glucose metabolism is the primary source of ATP production in cells, and it is well established that oxidative stress can influence the glucose metabolism of tumor cells. To assess this, we measured glucose concentrations in the culture supernatants of tumor cells. As shown in Figure 4B, the glucose concentration in the medium, normalized per cell, increased with longer durations of NIPP treatment. Notably, this effect persisted for up to 72 h following treatment. This observation may be explained by two factors: first, the number of cells in the NIPP-treated groups was lower than in the control group, resulting in reduced overall glucose consumption. Second, NIPP may directly alter the glucose metabolic pathways of tumor cells.

### 3.8. NIPP Upregulated Lactate Levels in the Media

Numerous studies have demonstrated that tumor cells often favor aerobic glycolytic, commonly known as the Warburg effect, even in the presence of sufficient oxygen, rather than fully entering the TCA cycle [30,31]. During this process, lactate, the end product of glycolysis, is typically secreted from the cells and can be transported back into mitochondria via monocarboxylate transporters (MCTs) [32,33]. NIPP generates a variety of ROS, which may further promote anaerobic metabolism in tumor cells. Given that lactate is the primary product of anaerobic respiration, we measured lactate concentrations in the culture medium at various time points following NIPP treatment. As shown in Figure 4C, lactate levels per cell increased significantly with longer treatment durations, suggesting that individual cells produced more lactate in a time-dependent manner after NIPP exposure.

### 3.9. NIPP Elevated LDH Activity of Tumor Cells

**Figure 5:**
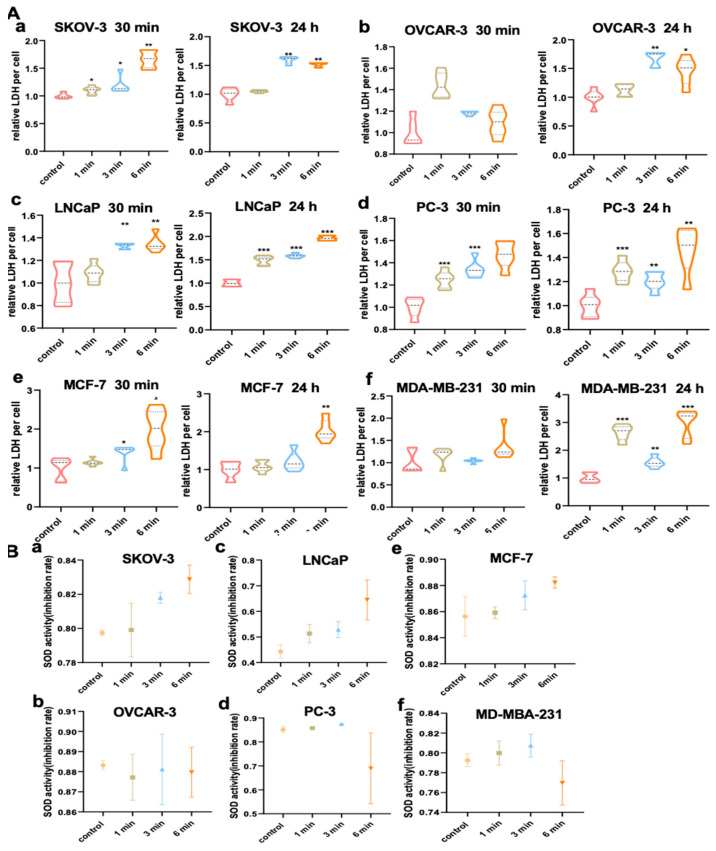
NIPP increases LDH release in tumor cell cultures and induced no significante alterations of SOD levels in tumor cell. (**A**): LDH relative level in the medium of SKOV-3 (**a**), OVCAR-3 (**b**), LNCaP (**c**), PC-3 (**d**), MCF-7 (**e**), and MDA-MB-231 (**f**) cells after the exposure to NIPP for 30 min and 24 h. (**B**): SOD relative level in the medium of SKOV-3 (**a**), OVCAR-3 (**b**), LNCaP (**c**), PC-3 (**d**), MCF-7 (**e**), and MDA-MB-231 (**f**) cells after the exposure to NIPP for 24 h. Every experiment was replicated at least 3 times. Each experimental group was compared with the control group. ***: *p* < 0.001, **: *p* < 0.01, *: *p* < 0.05.

Lactate dehydrogenase (LDH) is a key enzyme in glycolysis and belongs to the oxidoreductase family, catalyzing the transfer of electrons from one molecule (the reductant) to another (the oxidant). Specifically, LDH catalyzes the final step of glycolysis—the reversible conversion of pyruvate to lactate, along with the concomitant conversion of NADH to NAD^+^ [34]. Elevated LDH levels are not only considered a negative prognostic biomarker but also reflect its central role in cancer metabolism.In this study, we assessed intracellular LDH activity following NIPP treatment. As shown in Figure 5A, LDH activity increased significantly with longer treatment durations. Notably, in the 6 min treatment group, a significant increase in LDH activity was observed in SKOV3, LNCaP, PC-3, and MCF-7 cells as early as 30 min post-treatment, while OVCAR-3 and MDA-MB-231 cells did not show a significant change at that time point. However, by 24 h post-NIPP exposure, LDH activity was significantly elevated in all cell lines treated for 6 min, including OVCAR-3 and MDA-MB-231. In contrast, changes in LDH activity in the 1 min and 3 min treatment groups were less pronounced, though still statistically significant.

### 3.10. NIPP Did Not Alter SOD Activity of Tumor Cells

Superoxide dismutase (SOD) plays a critical role in maintaining the cellular redox balance by converting superoxide anions (O_2_^−^) into the less reactive species H_2_O_2_ and H_2_O [35,36]. Given that NIPP generates high levels of reactive oxygen species (ROS), which exert oxidative stress on tumor cells, we evaluated SOD activity following NIPP treatment. As shown in Figure 5B, our results indicate that SOD activity did not change significantly after treatment, even in cells exposed to NIPP for 6 min.

### 3.11. NIPP Did Not Induce HSP27 Expression in Tumor Cells

**Figure 6:**
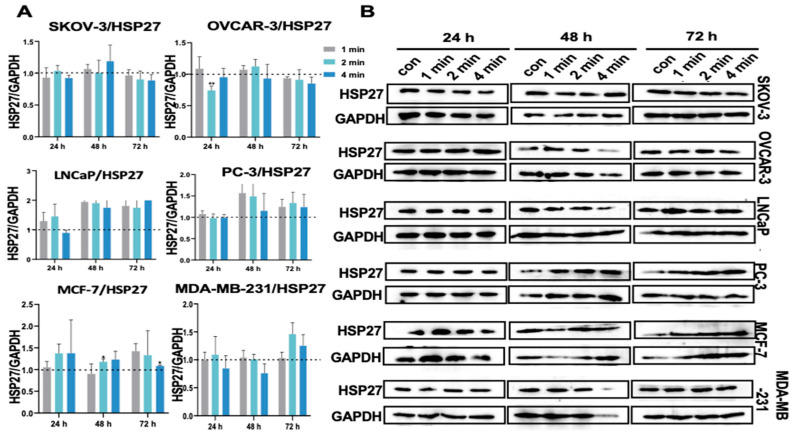
(**A**): Quantification of HSP27 relative expression level in SKOV-3, OVCAR-3, LNCaP, PC-3, MCF-7, and MDA-MB-231 cells after the exposure to NIPP for 24 h, 48 h, and 72 h. (**B**): Western blot signals. Each experiment was replicated at least 3 times. Experimental groups were compared to control group. The uncropped bolts are shown in Supplementary Materials.

HSP27 is known to play a significant role in the control of cytoskeletal organization [37,38] and is recognized for its antiapoptotic and antioxidant properties [39]. To examine the effects of NIPP on tumor cells, we utilized Western blotting to detect the expression levels of HSP27 protein on days 1, 2, and 3. As can be seen from Figure 6, no significant changes were observed in all the cell lines. This result suggests that, despite producing a series of ROS under the exposure of NIPP, they do not induce the expression of HSP27.

### 3.12. NIPP Did Not Significantly Change HSP40 Expression in Tumor Cells

**Figure 7:**
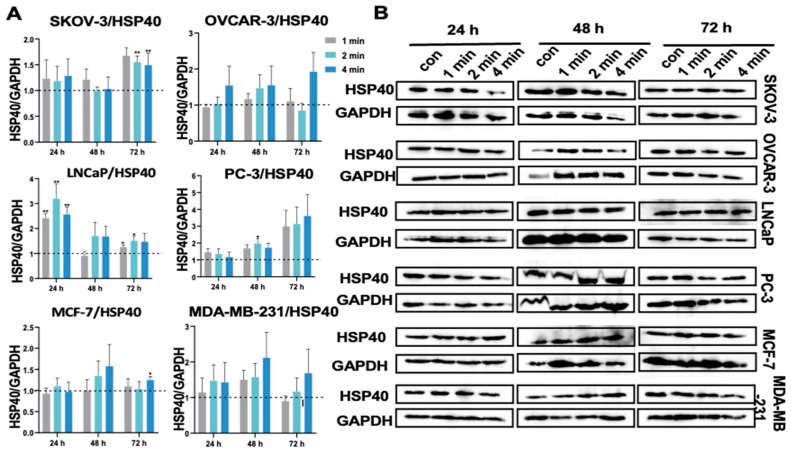
(**A**): Quantification of HSP40 relative expression level in SKOV-3, OVCAR-3, LNCaP, PC-3, MCF-7, and MDA-MB-231 cells after the exposure to NIPP for 24 h, 48 h, and 72 h. (**B**): Western blot signals. Each experiment was replicated at least 3 times. Experimental groups were compared to control group. **: *p* < 0.01, *: *p* < 0.05. The uncropped bolts are shown in Supplementary Materials.

HSP40 is known to potentially restore proteasomal function by reducing oxidative stress-induced damaged protein levels. Additionally, HSP40 reactivates ATP production levels from mitochondria [40]. HSP40 protein exerts a cytoprotective effect on cells and inhibits oxidative stress induced HSP70 degradation [40]. Barrett et al. [41] demonstrated that oxidative stress generated by mitochondria activates HSP70 and HSP40, characterized by the translocation of HSPs to the nucleus and increased protein content. Considering this, we examined HSP40 protein expression. The results (Figure 7) showed that HSP40 expression was significantly upregulated only in PC-3 cells on day 1 after NIPP treatment. In SKOV-3 cells, we observed an upregulation of HSP40 protein expression on day 3, but the increase was only about 1.5-fold, although statistically significant compared to the control group. No significant changes were observed in the SKOV-3, OVCAR-3, LNCaP, MCF-7, and MDA-MB-231 cell lines.

### 3.13. NIPP Did Not Alter HSP70 Expression in Tumor Cells

**Figure 8:**
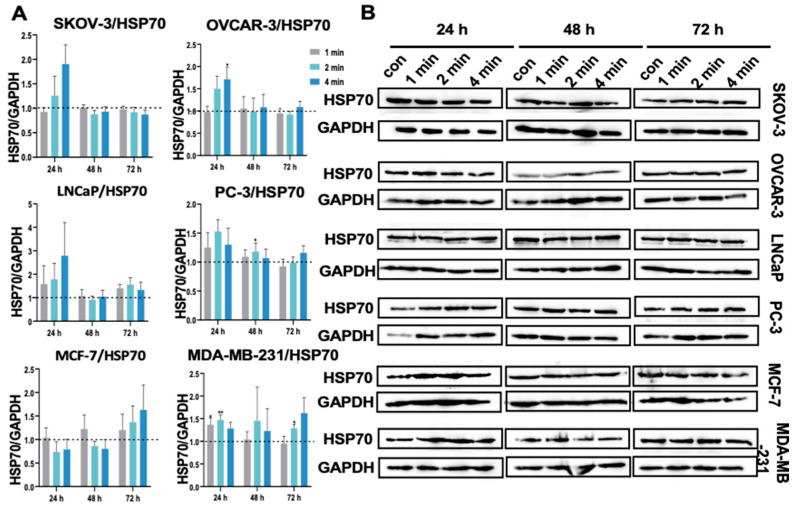
(**A**): Quantification of HSP70 relative expression level in SKOV-3, OVCAR-3, LNCaP, PC-3, MCF-7, and MDA-MB-231 cells after the exposure to NIPP for 24 h, 48 h, and 72 h. (**B**): Western blot signals. Each experiment was replicated at least 3 times. Experimental groups were compared to control group. **: *p* < 0.01, *: *p* < 0.05. The uncropped bolts are shown in Supplementary Materials.

Oxidative stress is known to be one of the factors that increase HSP70 levels [42]. Overexpression of HSP70 can counteract apoptosis caused by oxidative stress and produce cytoprotective effects [42,43]. In our current study, HSP70 protein expression was significantly upregulated only in OVCAR-3 cells in the 4 min group on day 1 after treatment with NIPP (Figure 8). In PC-3 and MDA-MB-231 cells, although some statistically significant changes were observed, the extent of upregulation was not substantial.

### 3.14. NIPP Did Not Induced HSP90α Expression in Tumor Cells

**Figure 9:**
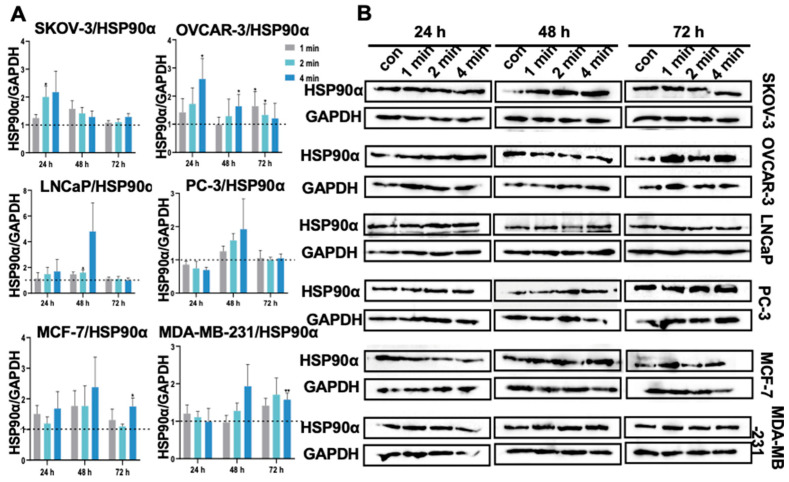
(**A**): Quantification of HSP90α relative expression level in SKOV-3, OVCAR-3, LNCaP, PC-3, MCF-7 cells, and MDA-MB-231 after the exposure to NIPP for 24 h, 48 h, and 72 h. (**B**): Western blot signals. Each experiment was replicated at least 3 times. Experimental groups were compared to control group. They were statistically evaluated with Student *t*-test. **: *p* < 0.01, *: *p* < 0.05. The uncropped bolts are shown in Supplementary Materials.

Under conditions of cellular stress, the expression of the HSP90α protein is known to increase. HSP90α is also upregulated in various cancers [44]. In leukemia, HSP90α levels are upregulated and have been correlated with disease prognosis [45]. Elevated levels of HSP90α also indicate poor prognosis in breast and pancreatic cancer [46,47]. Therefore, elevated expression of HSP90α in tumors may serve as a prognostic indicator as well as a potential target for therapy [48]. Our current study primarily detected the HSP90α expression. The results showed that no significant changes occurred after NIPP exposure (Figure 9).

### 3.15. NIPP Did Not Boost HSP90β Expression in Tumor Cells

**Figure 10:**
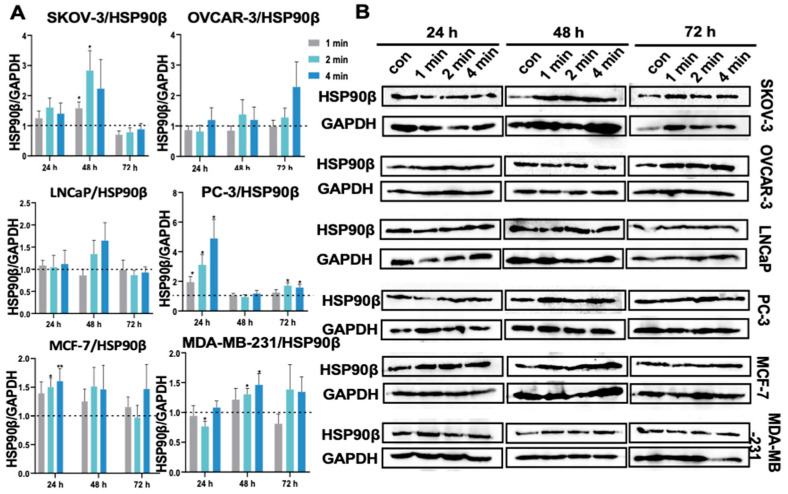
(**A**): Quantification of HSP90β relative expression level in SKOV-3, OVCAR-3, LNCaP, PC-3, MCF-7 cells, and MDA-MB-231 after the exposure to NIPP for 24 h, 48 h, and 72 h. (**B**): Western blot signals. Each experiment was replicated at least 3 times. Experimental groups were compared to control group. They were statistically evaluated with Student *t*-test. **: *p* < 0.01, *: *p* < 0.05. The uncropped bolts are shown in Supplementary Materials.

Stress has been reported to induce high expression of HSP90α, while HSP90β is constitutively expressed. Although HSP90β is predominantly overexpressed in various types of cancer, some cancers appear to be associated with cancer cell survival in the absence of HSP90β, despite the inhibition of HSP90β expression [49]. Some normal tissues, as well as cancer cells, are primarily dependent on HSP90β. For instance, hepatocytes and hepatocellular carcinoma cells primarily require HSP90β, specifically for vascular endothelial growth factor receptor (VEGFR)-mediated angiogenesis [50,51]. Meng and colleagues evaluated angiogenesis in hepatocellular carcinoma following HSP90α or HSP90β knockdown and observed that HSP90β inhibitors inhibited VEGFR-mediated angiogenesis [50,51]. We detected the protein expression levels to understand the expression of HSP90β after NIPP treatment. As shown in Figure 10, SKOV-3, PC-3, and MDA-MB-231 cells showed statistically significant elevated HSP90β expression levels among the six cell lines we tested, but there was no general trend.

## 4. Discussion

The investigation of non-invasive physical plasma (NIPP) efficacy in tumor cells has gained increasing attention in recent years. However, the mechanisms by which NIPP inhibits tumor cell growth and function remain incompletely understood. To address this, we systematically analyzed and compared the effects of NIPP on six tumor cell lines representing three malignancies: ovarian (OVCAR-3 and SKOV-3), prostate (LNCaP and PC-3), and breast cancer (MCF-7 and MDA-MB-231). These cell line pairings are well-established in vitro models that differ in clinically relevant features: cisplatin sensitivity (OVCAR-3/SKOV-3), androgen receptor status (LNCaP/PC-3), and hormone receptor profiles (MCF-7/MDA-MB-231) [52,53]. In this study, we observed that all six cell lines, regardless of their genotype, were sensitive to NIPP. This suggests that NIPP has a broad spectrum of tumor-killing effects and is not limited to tumors of a specific genotype.

Our results demonstrate that NIPP generated by the Plasma Care device significantly inhibits proliferation and migration in all tested tumor cell lines and induces cytoskeletal disorganization. With prolonged NIPP treatment, the inhibitory effects on tumor cell proliferation and migration were significantly enhanced, accompanied by pronounced disruption of the cytoskeleton. These findings were consistent with the observations reported by Li et al. [54] in Human Embryonic Kidney 293 (HEK293) cells, demonstrating comparable inhibitory effects on cellular proliferation and migration. Notably, while their study employed HEK293 cells as a nonmalignant model, our investigation utilized tumor cell lines. This distinction underscores the importance of defining a therapeutic window for NIPP applications, ensuring effective tumor cell eradication while sparing normal tissues. Given these parallel yet distinct findings, careful consideration of safety thresholds and cell-type specificity is warranted when translating NIPP to clinical or therapeutic settings. The use of the “equivalent total oxidation potential (ETOP)” as a standardized chemical dose unit for plasma medicine, which effectively unified the biological effects of different plasma sources into a measurable quantitative indicator [55].

Consistent with previous studies [56,57], we confirmed that NIPP inhibits tumor cell proliferation and migration. Utilizing a clinically approved NIPP device, we observed similar inhibitory effects across various tumor cell types (Figure 3). Since the cytoskeleton is critical for cell motility, these observations align with earlier work by Haralambiev et al. [58] and Jacoby et al. [59], who showed that NIPP alters the actin cytoskeleton in sarcoma cells. Our findings extend this concept by demonstrating cytoskeletal disruption in breast, ovarian, and prostate cancer cells (Figure 4A).

NIPP generates substantial amounts of ROS, including H_2_O_2_, NO_2_^−^, NO_3_^−^, and ONOO^−^, which can damage mitochondria and initiate apoptosis through the mitochondrial pathway [60,61,62,63]. Hong et al. [13] reported that NIPP induces caspase-3–mediated apoptosis in HT29 and A549 cells. Conversely, Marches et al. [64] found that prolonged NIPP exposure (>60 s) in keratinocytes led to cytotoxicity, impaired migration, upregulation of HSP27, and oxidative stress. In contrast, short-term exposures (<60 s) had no cytotoxic effects and even promoted keratinocyte migration and wound healing, without impairing mitochondrial function. However, we could not confirm these findings from nonmalignant cells in our study with cancer cells.

In breast, ovarian, and prostate cancer cell lines, NIPP reduced MMP and altered glucose metabolism. Tahmasebi et al. [65] similarly showed that NIPP increased ROS, collapsed MMP, caused mitochondrial swelling, and induced cytochrome c release in cancerous, but not in nonmalignant, rat ocular mitochondria. Notably, their device used helium-based plasma, whereas our study used ambient-air plasma from the Plasma Care system. This distinction is important: different NIPP technologies and carrier gases yield different ROS compositions and biological effects. Therefore, direct comparisons between devices are limited, and future plasma medicine research must systematically investigate these differences to match devices with specific clinical needs.

LDH is a crucial enzyme in the glycolytic pathway, which is the metabolic process that breaks down glucose into pyruvate. LDH is a key glycolytic enzyme that catalyzes the conversion of pyruvate to lactate under anaerobic conditions and regenerates NAD^+^ from NADH to sustain glycolysis [66,67,68]. Our experiments confirmed increased LDH activity following NIPP treatment. These findings are consistent with reports showing that NIPP, when combined with temozolomide, enhances LDH activity in glioblastoma cells (T98G and A172) [69]. We also observed increased lactate accumulation in the culture medium. This suggests that due to mitochondrial dysfunction, tumor cells may shift toward enhanced anaerobic glycolysis, a hallmark of the Warburg effect [17]. Additionally, since lactate is typically recycled into the TCA cycle via monocarboxylate transporters (MCTs), impaired mitochondrial function may reduce this recycling, further exacerbating metabolic imbalance.

The presence of SOD in mitochondria helps to maintain a balance between ROS production and the cell’s antioxidant defense mechanisms. This balance is critical for cellular homeostasis. SOD is a key mitochondrial antioxidant enzyme that protects mitochondrial DNA, lipids, and proteins by converting superoxide radicals into H_2_O_2_ and water [70]. However, we observed no significant upregulation of SOD activity after NIPP treatment. This contrasts with conventional therapies such as radiotherapy and chemotherapy, which typically activate these stress-response systems and contribute to therapy resistance. The absence of SOD upregulation suggests that NIPP-induced oxidative stress overwhelms tumor antioxidant defenses without activating classical protective pathways. Mechanistically, this indicates that tumor cells are unable to mount a sufficient adaptive stress response, rendering them particularly vulnerable to NIPP. This aligns with prior reports suggesting that NIPP-derived ROS can overwhelm antioxidant defenses, SOD, glutathione, and catalase, leading to DNA double-strand breaks, cell cycle arrest, and apoptosis via the mitochondrial or TNF receptor pathway [26,71,72]. Other antioxidant systems, including glutathione peroxidase and catalase, are similarly affected. A decreased GSH/GSSG ratio and reduced NADPH/NADP^+^ ratio have also been reported in NIPP-treated cancer cells [73]. A correlation also exists between antioxidant system (including SOD) and autophagy, low or inhibited SOD activity causes intense oxidative stress. This stress can overstimulate autophagy, a process that, by surpassing its degradative capacity, consumes essential cellular components and thereby initiates autophagic cell death [74]. The precise mechanism driving this phenomenon remains an open question and a key objective for future research.

NIPP exerts its effects primarily through ROS-induced redox stress, which disrupts mitochondrial function and can trigger HSP expression [75]. However, studies investigating the interaction between NIPP and HSPs remain limited. In this study, we examined the expression of several HSP family members following NIPP treatment. We found no evidence for a systematic or significant upregulation of HSPs, in contrast to some earlier studies. Despite transient elevations in HSP levels at specific time points in some cell lines, the increases were not sustained. Although in other studies, NIPP has been shown to upregulate HSP90 in burn patients [76], induce HSP aggregation in yeast [77], increase HSP27 expression in keratinocytes [64], and elevate HSP60 in osteosarcoma cells in response to elevated H_2_O_2_ [78]. Nevertheless, a broad, protective HSP response has not been consistently observed across cell types. The transcriptional upregulation of HSPs is principally governed by the activation of heat shock factor 1 (HSF1) [79]. It is postulated that supraphysiological levels of oxidative stress can directly oxidize and inhibit HSF1, blocking its transformation into a transcriptionally active trimer and its subsequent import into the nucleus. Concurrently, extant HSP pools may suffer direct oxidative damage from high concentrations of ROS, notably the highly reactive hydroxyl radical (•OH) and peroxynitrite (ONOO^−^). This compromises HSP functionality and tags them for proteasomal degradation. A further layer of complexity is added by the potent induction of autophagy following plasma exposure. While autophagy acts as a pro-survival mechanism by eliminating compromised organelles during mild stress, it can become cytotoxic under intense stress conditions [80]. In this hyperactivated state, it non-selectively degrades cellular components, including HSPs, ultimately overwhelming the protein synthesis machinery and creating a net loss of protective HSPs. Ultimately, a key outcome of NIPP is the absence of a significant increase in protective HSPs within tumor cells. This failure to mount a protective stress response contributes to the efficacy of NIPP in killing cancer cells.

**Figure 11:**
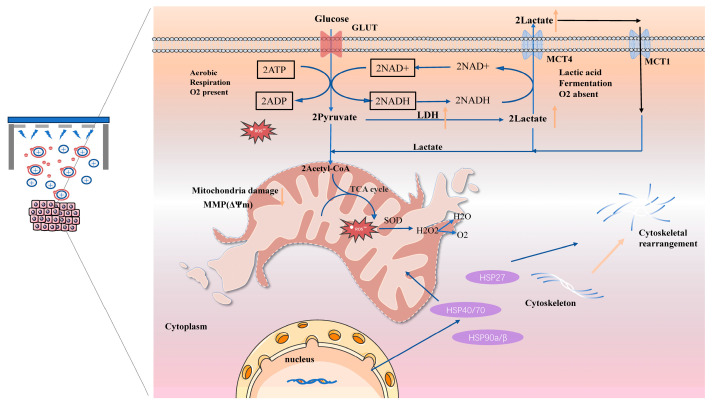
The pathway of NIPP impact on tumor cells. During the discharge process in Plasma care, a significant amount of ROS is generated. These ROS enter tumor cells, disrupt the mitochondria within the tumor cells, and hinder the aerobic glucose metabolism process, leading to increased anaerobic metabolism of glucose and the production of a substantial amount of lactate. Additionally, this process results in an increase in LDH activity, while SOD remains largely unchanged. Furthermore, ROS also alters the cell cytoskeleton, leading to the rearrangement of the cytoskeleton, which subsequently affects the migration and invasive abilities of tumor cells. These processes are not significantly inhibited by the short-term, minor elevation in the expression of heat shock proteins.

Considering all existing cell biological analyses of the cellular and molecular NIPP effect, there also appear to be differences between malignant and nonmalignant cells. Our future work will rigorously test the selectivity of NIPP by employing a co-culture system of cancer and normal cells, as well as by comparing its effects on a range of non-malignant cells. According to a common hypothesis, malignant cells react more sensitively to NIPP exposure, producing more endogenous ROS due to increased metabolism. As a result, the ROS detoxification systems are significantly more stressed, and additional NIPP-induced ROS may no longer be degraded as effectively. Despite clear oxidative stress, neither the expression of HSPs nor the activity of the antioxidant enzyme SOD was significantly altered. This indicates a lack of activation of classical cytoprotective pathways that are commonly induced by conventional treatments such as radiotherapy and chemotherapy. And, the failure to upregulate the HSP system suggests that NIPP bypasses typical cellular stress responses and avoids triggering mechanisms often responsible for therapy resistance. Mechanistically, the loss of tumor cell viability can be attributed to multifactorial interference with mitochondrial function and glucose metabolism. The disruption of energy production, coupled with sustained oxidative damage, leads to mitochondrial dysfunction and metabolic collapse. The pathway of NIPP impact on tumor cells as Figure 11. Our observations suggest a potential link between ROS produced by NIPP and HSPs; however, future studies are required to elucidate the underlying causal mechanism.

## 5. Conclusions

Plasma Care, as a newly developed handheld, portable, NIPP generation device, has excellent portability and clinical operability, but its related basic research is still in the preliminary stage. Therefore, in-depth exploration of the biological effects of NIPP generated by Plasma Care on different types of cells, especially tumor cells, is of great significance for expanding its potential application in tumor treatment. NIPP, applied via the Plasma Care device, demonstrated significant anticancer effects in ovarian, prostate, and breast cancer cell lines in our present study. Figure 11 shows the pathway of NIPP impact on tumor cells. NIPP treatment inhibited cellular proliferation and migration, disrupted cytoskeletal organization, and induced marked metabolic disturbances in a time-dependent manner. These effects were closely associated with elevated intracellular ROS, decreased MMP, enhanced glycolysis, and increased lactate production. Notably, despite clear oxidative stress, neither the expression of HSPs nor the activity of the antioxidant enzyme SOD was significantly altered. This indicates a lack of activation of classical cytoprotective pathways that are commonly induced by conventional treatments such as radiotherapy and chemotherapy. The failure to upregulate the HSP system suggests that NIPP bypasses typical cellular stress responses and avoids triggering mechanisms often responsible for therapy resistance. Mechanistically, the loss of tumor cell viability can be attributed to multifactorial interference with mitochondrial function and glucose metabolism. The disruption of energy production, coupled with sustained oxidative damage, leads to mitochondrial dysfunction and metabolic collapse. Collectively, these findings highlight the unique therapeutic potential of NIPP as a novel anticancer approach. By simultaneously inducing oxidative stress and metabolic impairment, while circumventing HSP-mediated resistance, NIPP offers a promising strategy to improve cancer treatment efficacy. Further in vivo and clinical investigations are warranted to explore its integration into existing oncological protocols.

## Data Availability

The original contributions presented in this study are included in the article. Further inquiries can be directed to the corresponding author.

## Supplementary Materials

The following supporting information can be downloaded at: https://www.mdpi.com/article/10.3390/cancers17213517/s1, The uncropped bolts are shown in Supplementary Materials.

## Abbreviations

The following abbreviations are used in this manuscript:

DBD	Dielectric Barrier Discharge
DOX	Doxorubicin
GSH	Glutathione
GSSG	Oxidized Glutathione
GLUT	Glucose Transporter
HSP	Heat Shock Protein
LDH	Lactate Dehydrogenase
MCT	Monocarboxylate Transporters
MMP	Mitochondrial Membrane Potential
NIPP	Non-invasive Physical Plasma
PBS	Phosphate-Buffered Saline
ROS	Reactive Oxygen Species
SOD	Superoxide Dismutase
TCA	Tricarboxylic Acid Cycle
