# Supplementary material for: Non-Invasive Physical Plasma as an Oncological Therapy Option: Modulation of Cancer Cell Growth, Motility, and Metabolism Without Induction of Cancer Resistance Factors
Source: Cancers (Basel). 2025 Oct 31;17(21):3517. doi: 10.3390/cancers17213517 (PMC12607350; doi:10.3390/cancers17213517)

LNCaP HSP27 day1

I incubated antibody for HSP27 first.
After scan HSP 27, washed the blots , then incubated with antibody for GAPDH

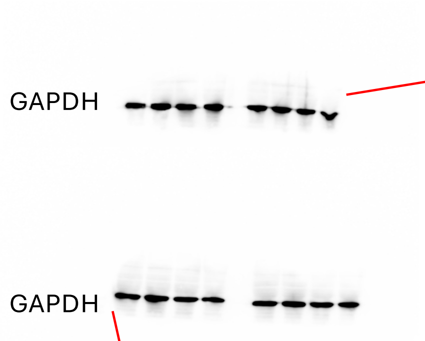


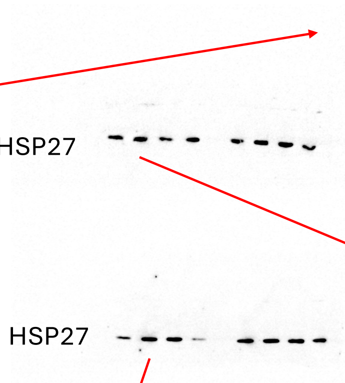


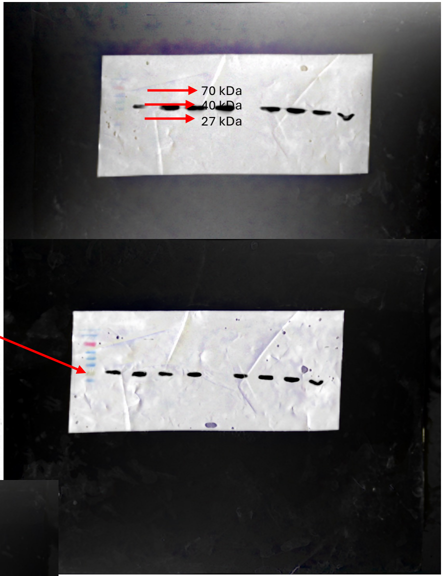


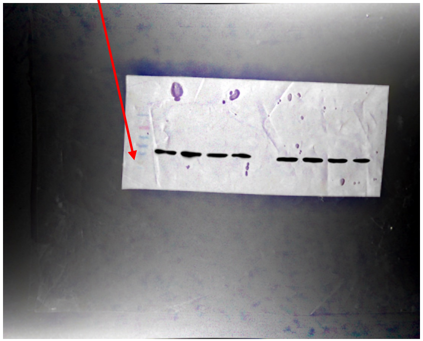

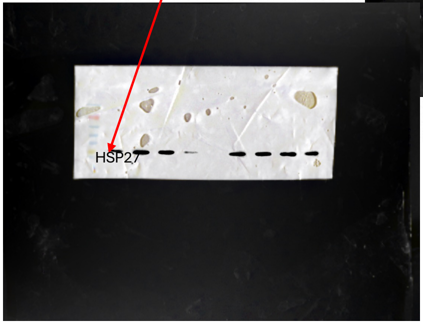

LNCaP HSP27 day2

GAPDH

HSP 27

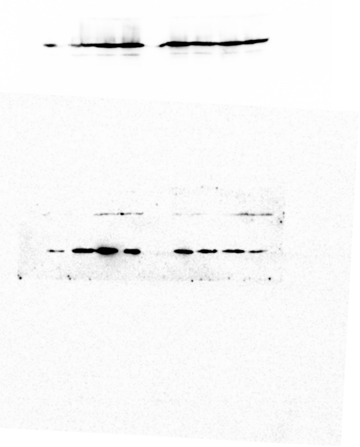

LNCaP  HSP27 day3

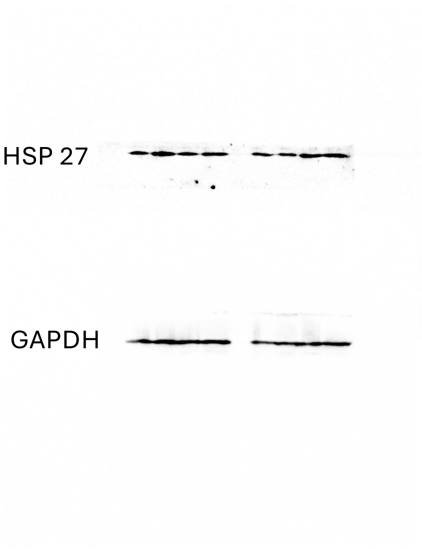

LNCaP  HSP40 day1

HSP40

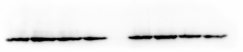

GAPDH

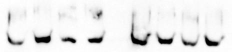

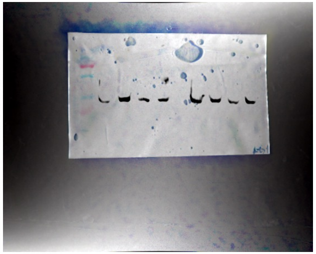

HSP40

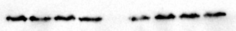

GAPDH

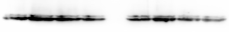

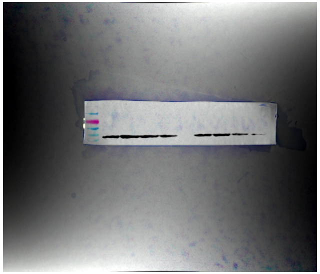

LNCaP  HSP40 day2

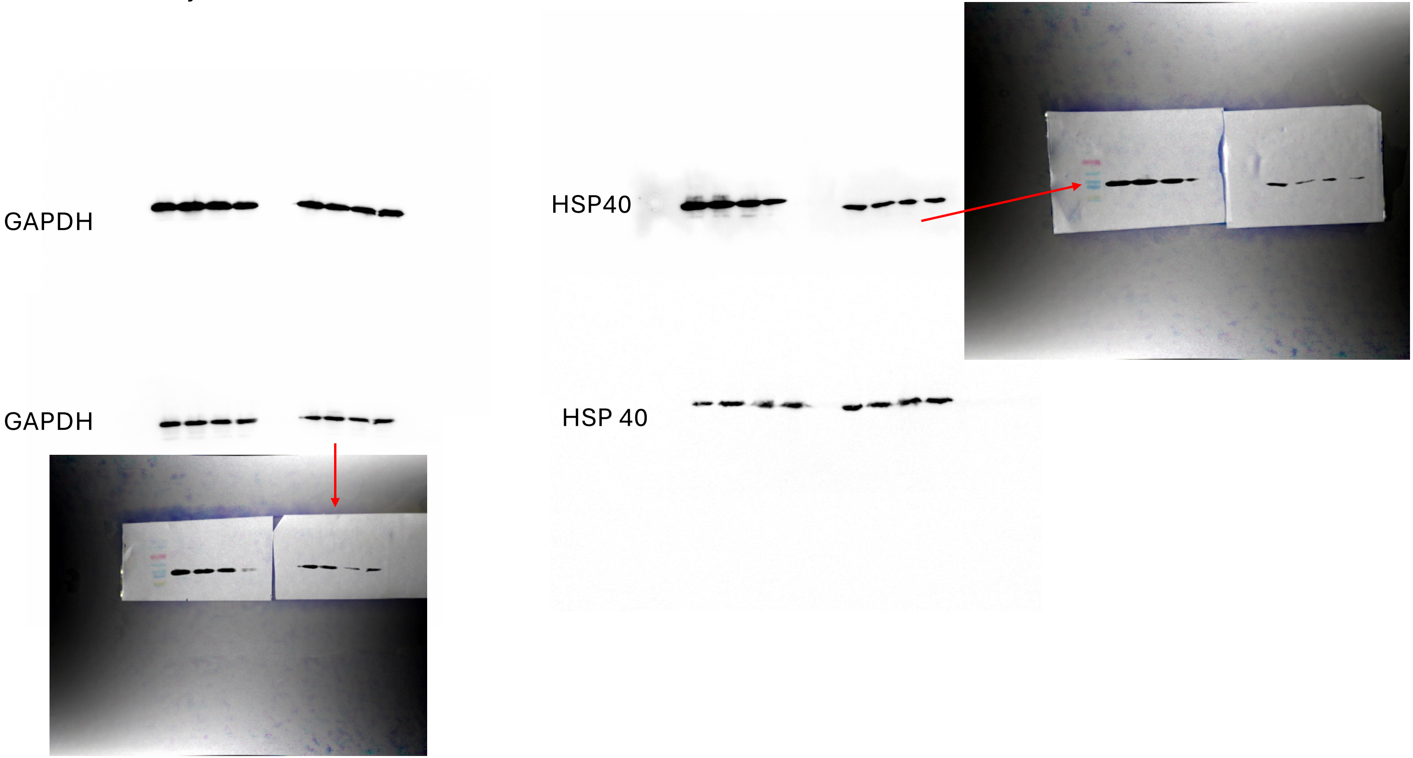

LNCaP HSP40 day3

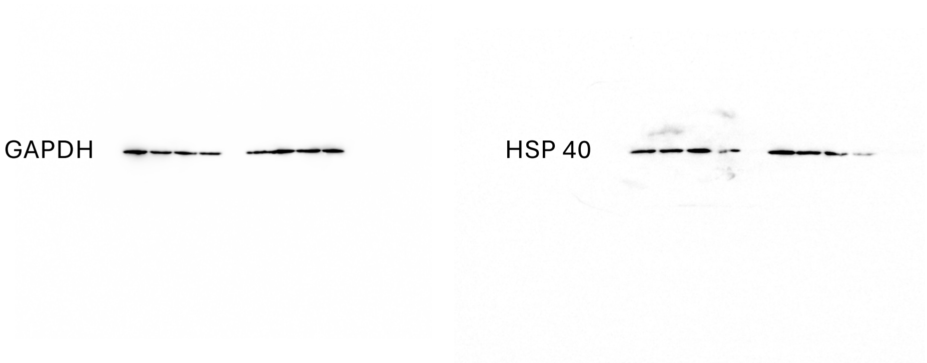

LNCaP HSP70 day1

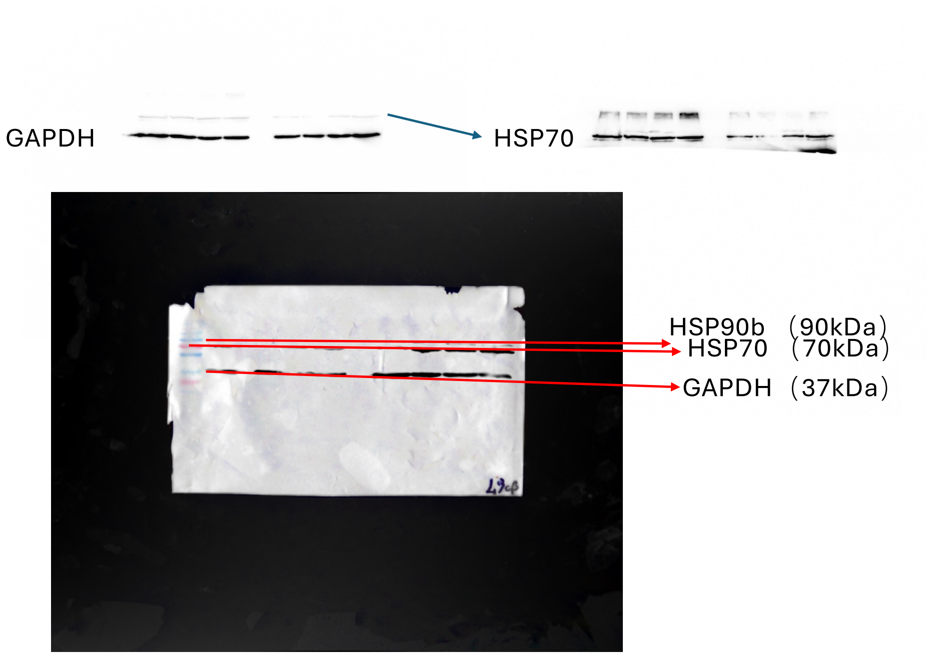

LNCaP  HSP70 day2

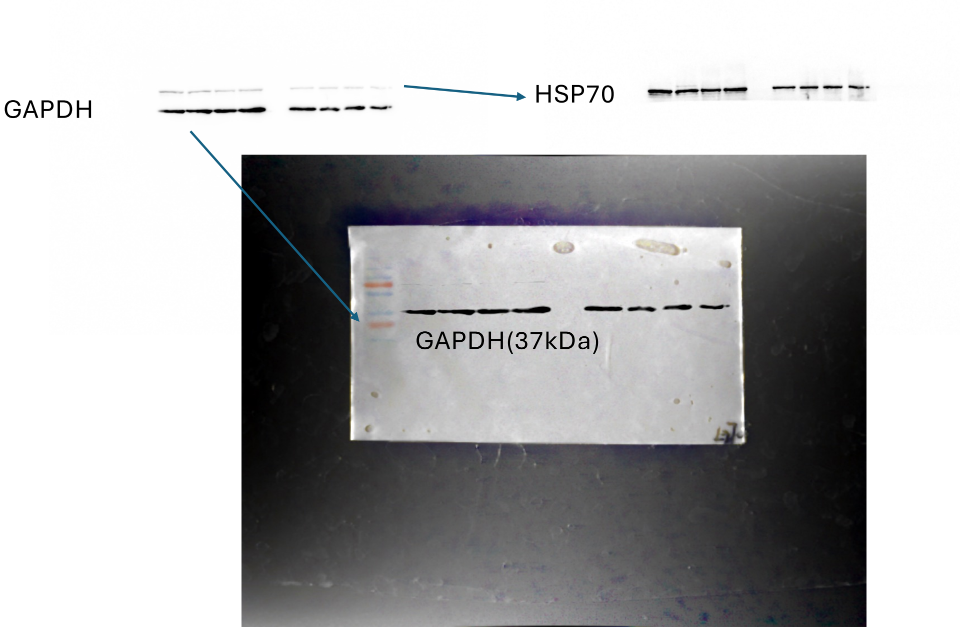

LNCaP HSP70 day3

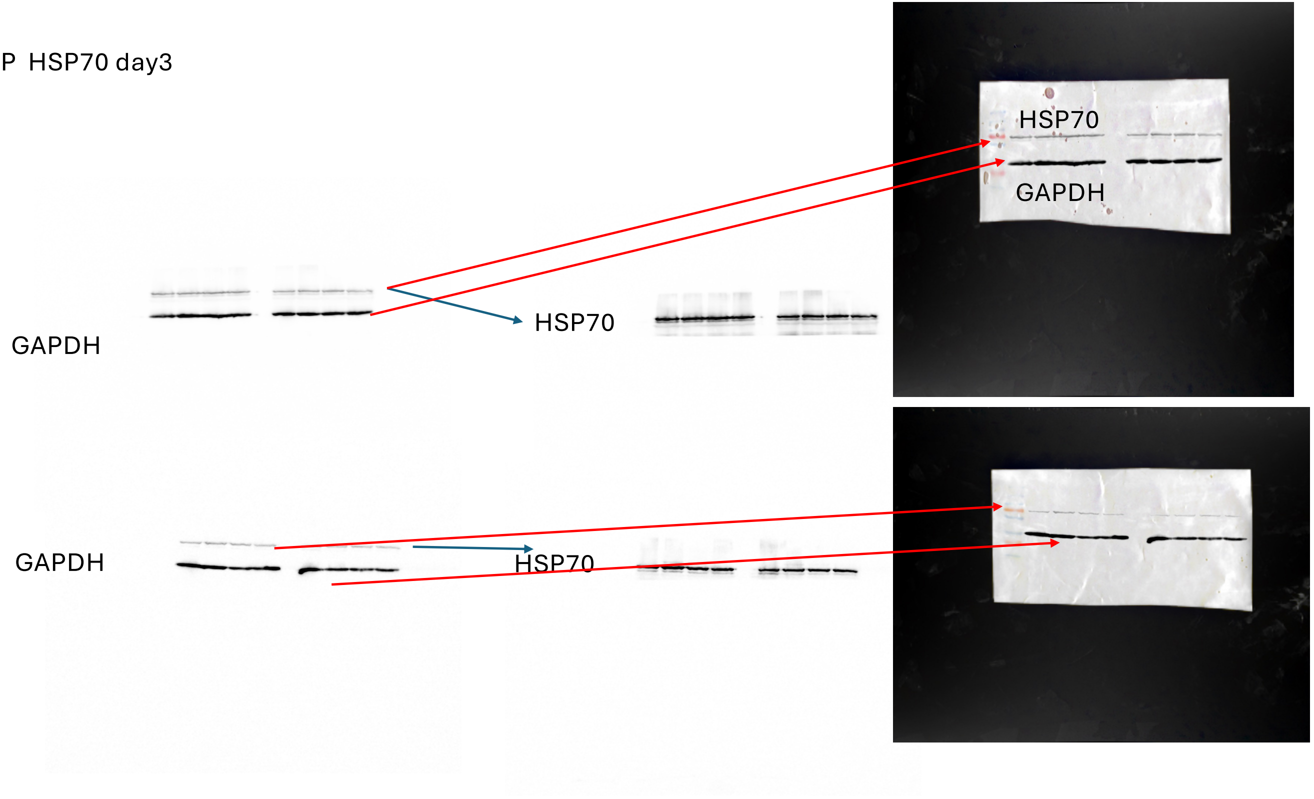

LNCaP  HSP90a day1

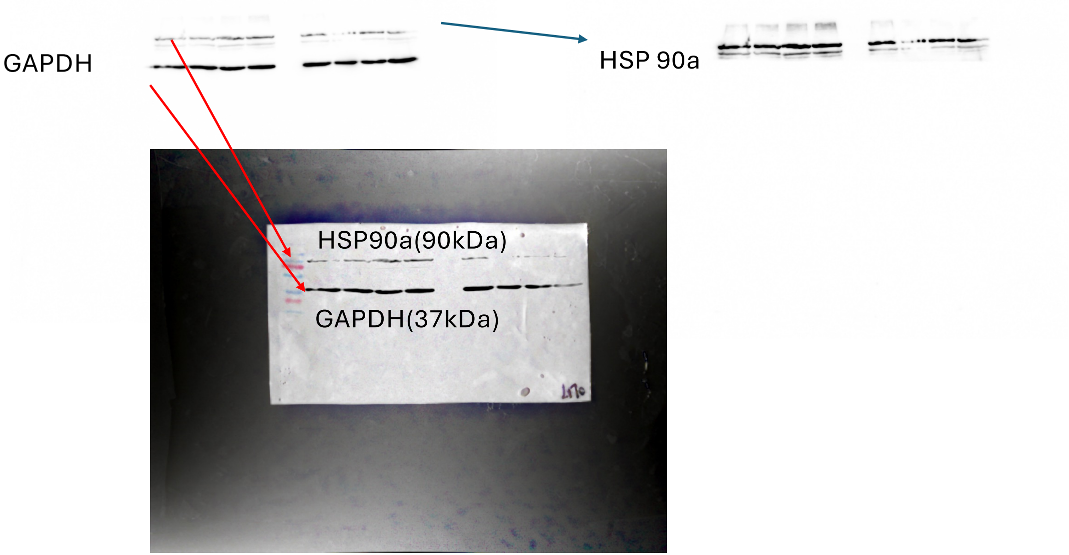

LNCaP HSP90a day2

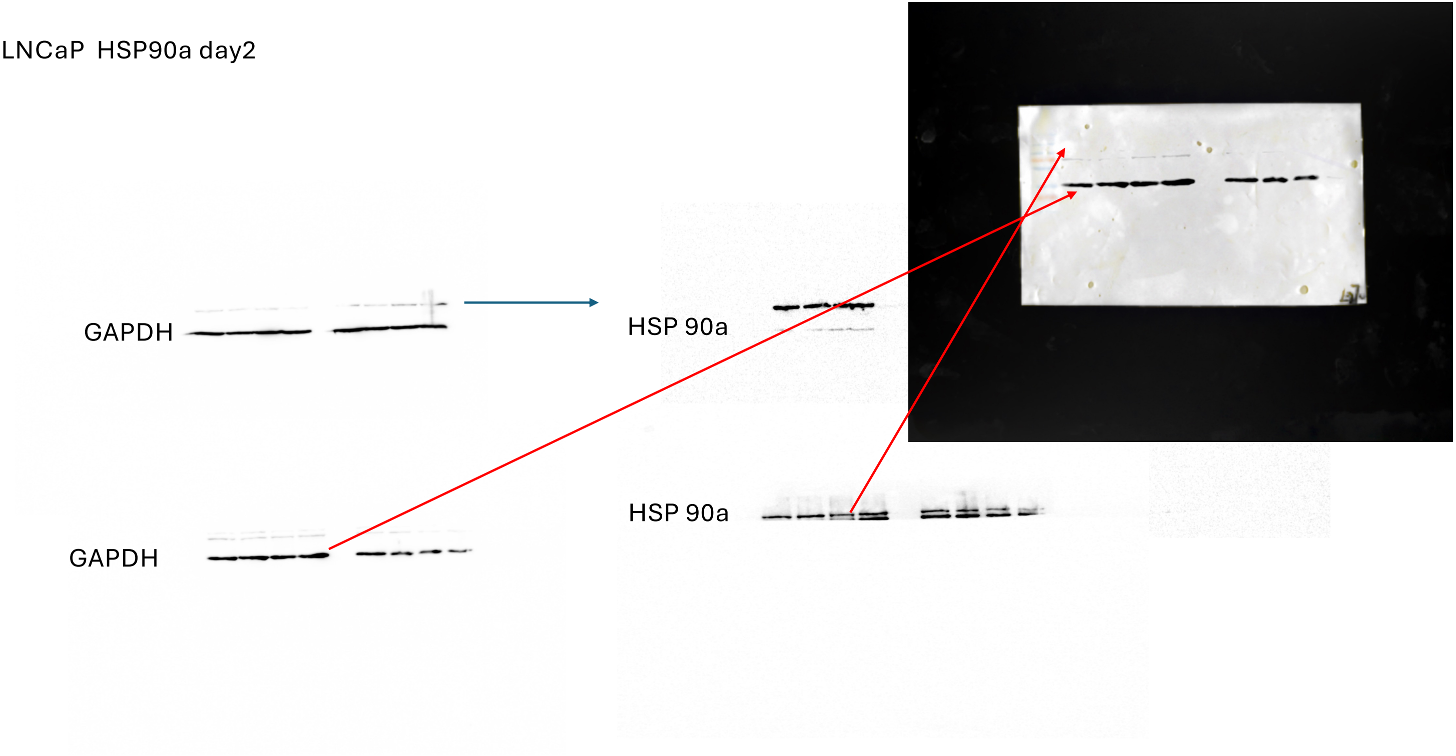

LNCaP  HSP90a day3

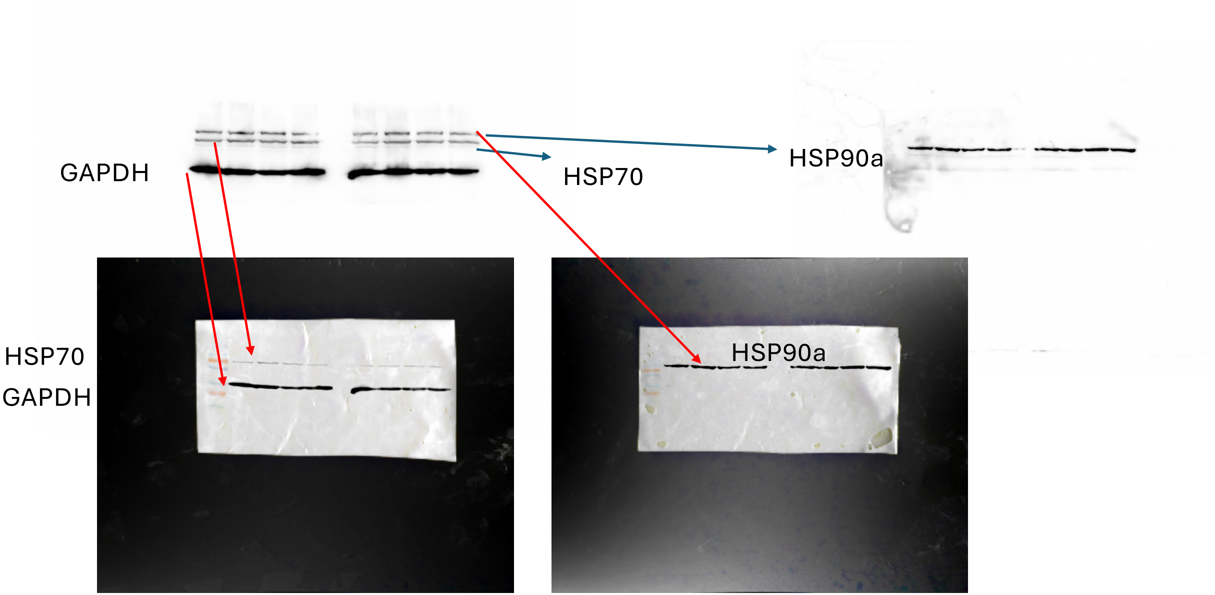

LNCaP HSP90b day1

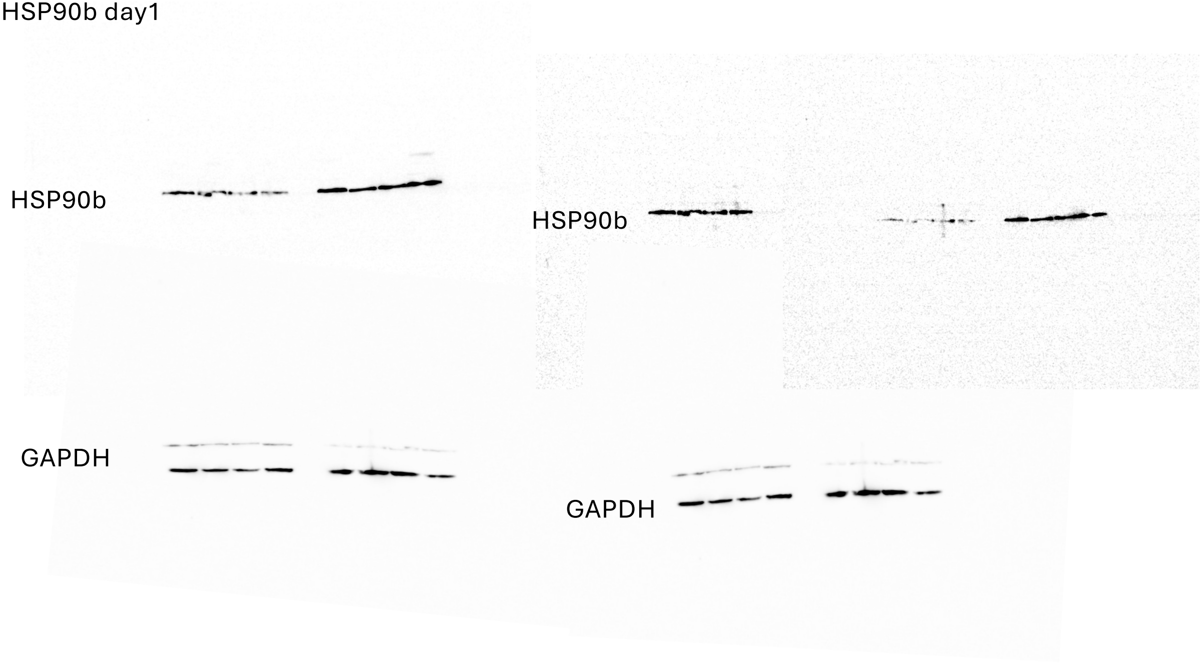

LNCaP HSP90b day2

GAPDH

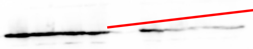

GAPDH

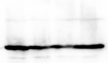

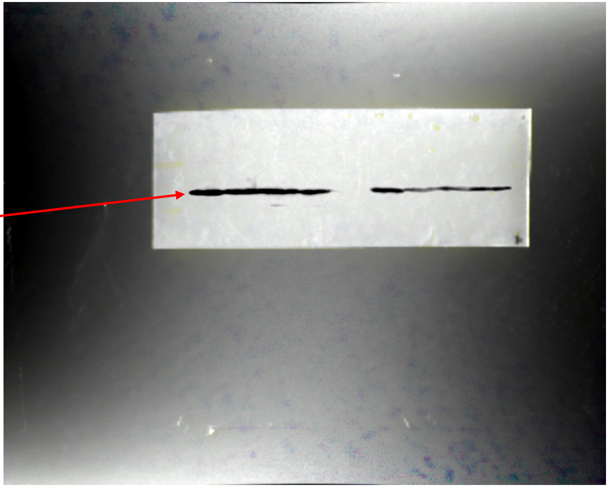

HSP90b

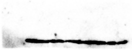

HSP90b

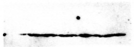

LNCaP HSP90b day3

GAPDH

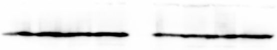

HSP90b

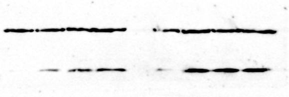

GAPDH

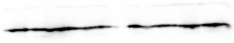

HSP90b

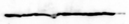

Supplement: Supplementary file 1 [file cancers-17-03517-s001.zip › cancers-3776093-supplementary/RAW BLOTS LNCaP.pdf]
