# Supplementary material for: Non-Invasive Physical Plasma as an Oncological Therapy Option: Modulation of Cancer Cell Growth, Motility, and Metabolism Without Induction of Cancer Resistance Factors
Source: Cancers (Basel). 2025 Oct 31;17(21):3517. doi: 10.3390/cancers17213517 (PMC12607350; doi:10.3390/cancers17213517)

MCF-7 HSP 27 day1

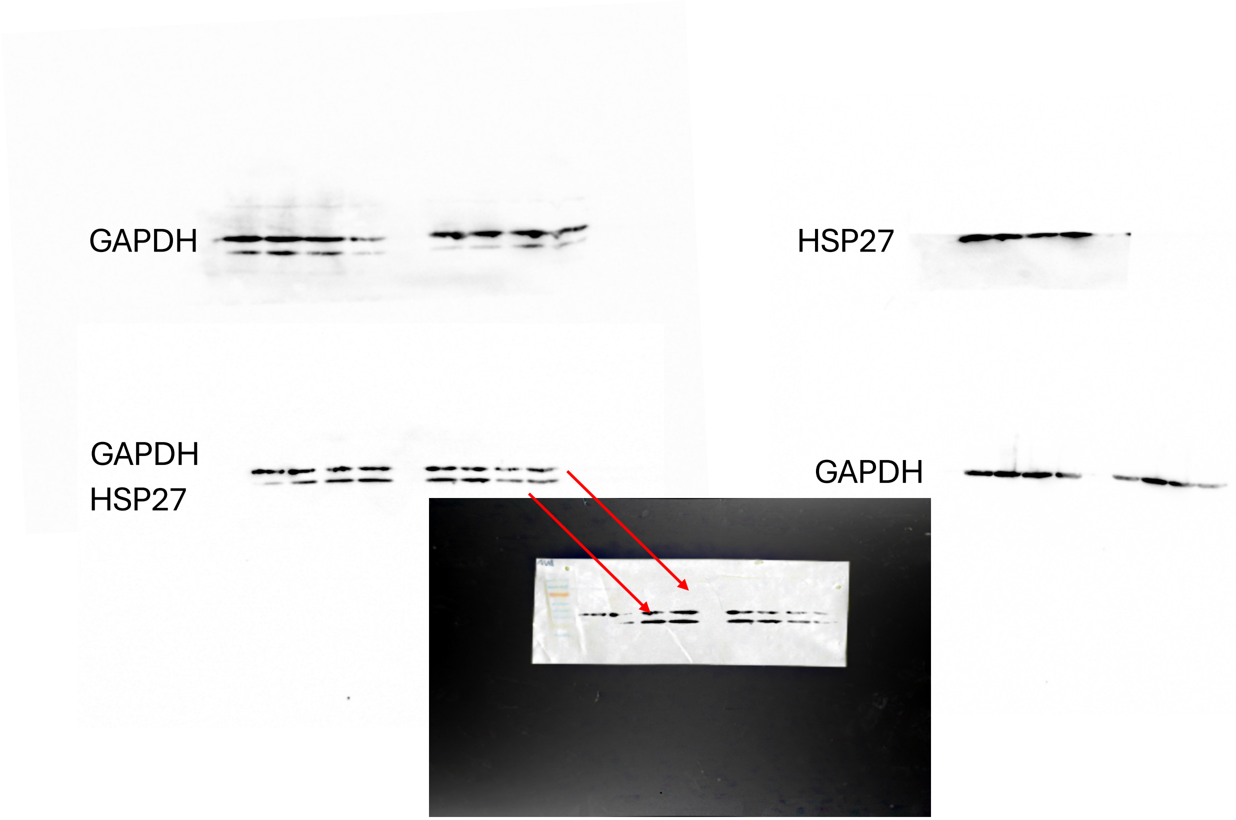

MCF-7 HSP 27 day2

GAPDH

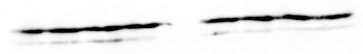

HSP27

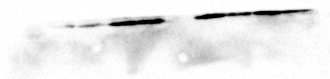

GAPDH

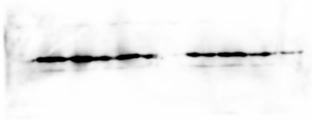

HSP 27

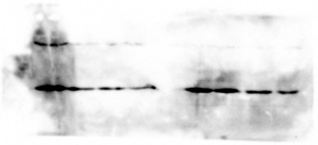

MCF-7 HSP 27 day3

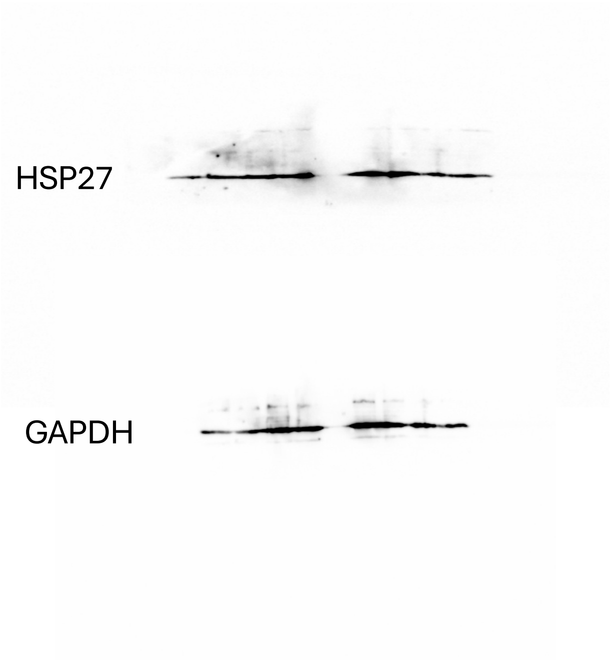

MCF-7 HSP 40 day1

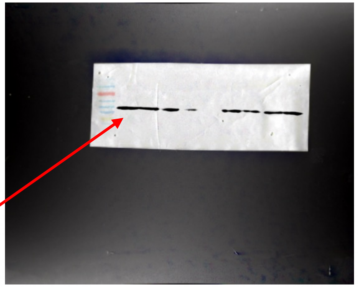

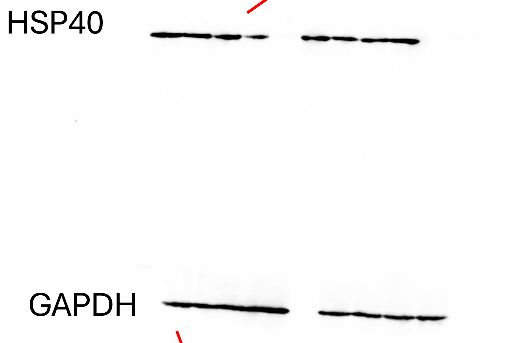

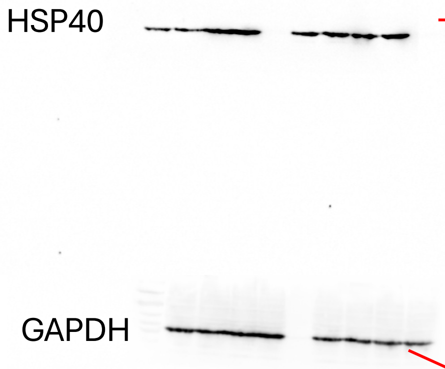

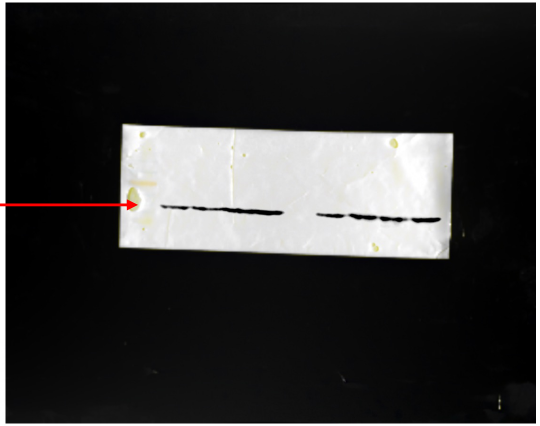

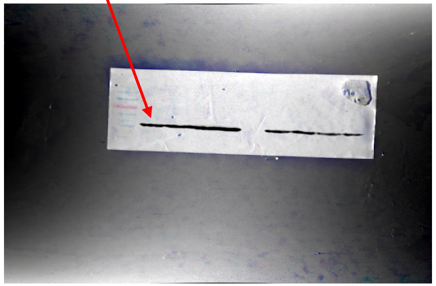

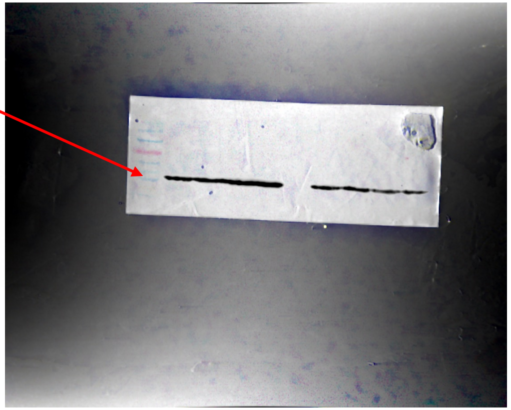

MCF-7 HSP 40 day2

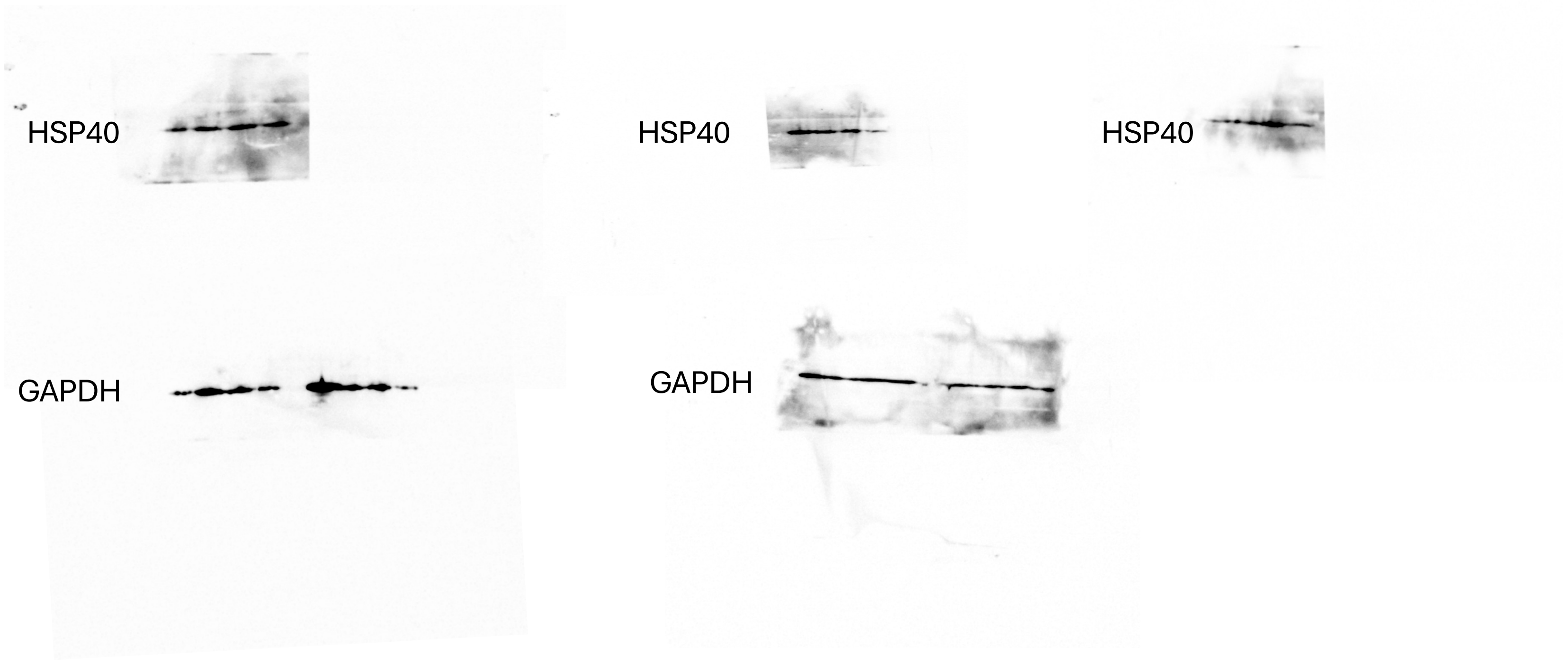

MCF-7 HSP 40 day3

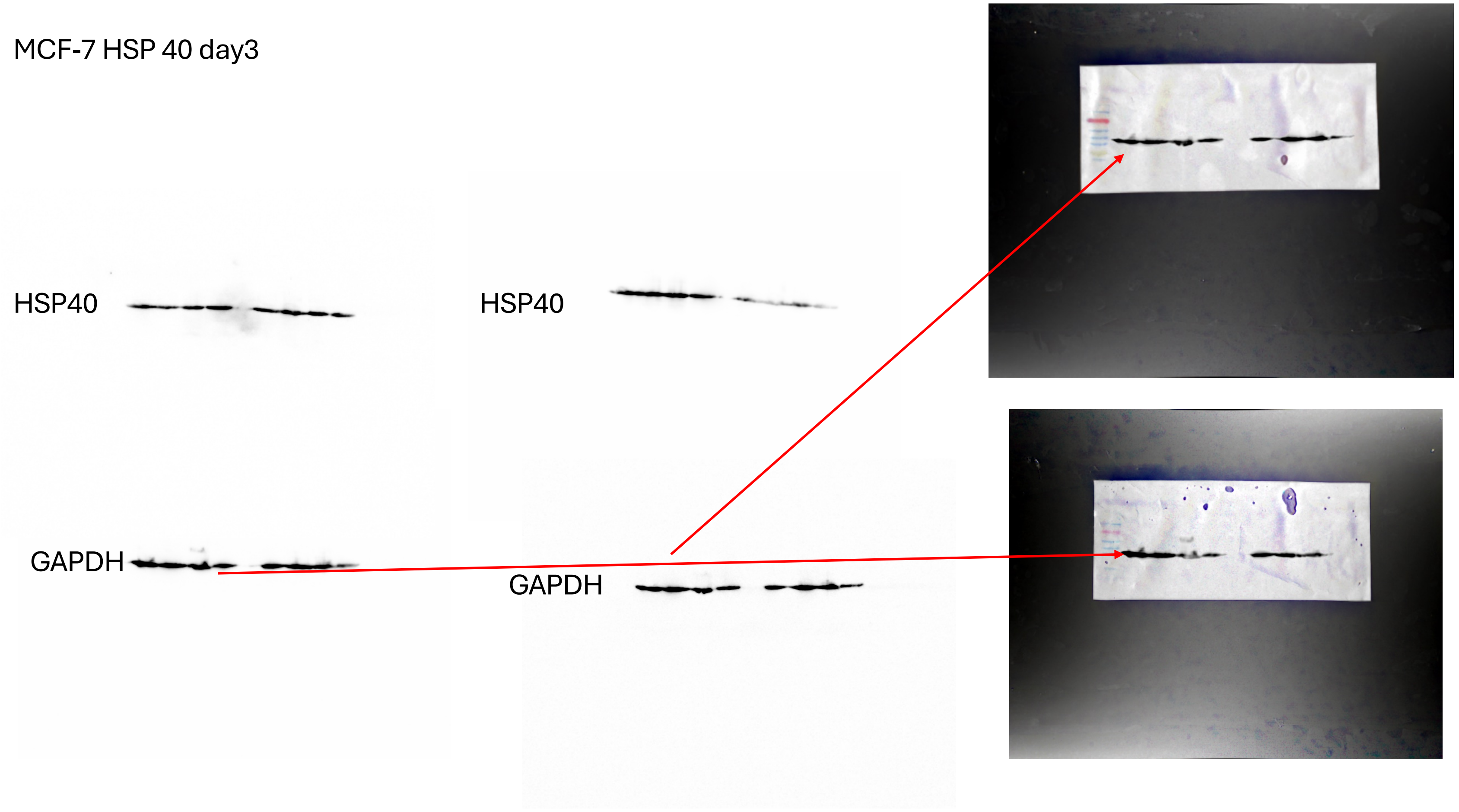

MCF-7 HSP70 day1

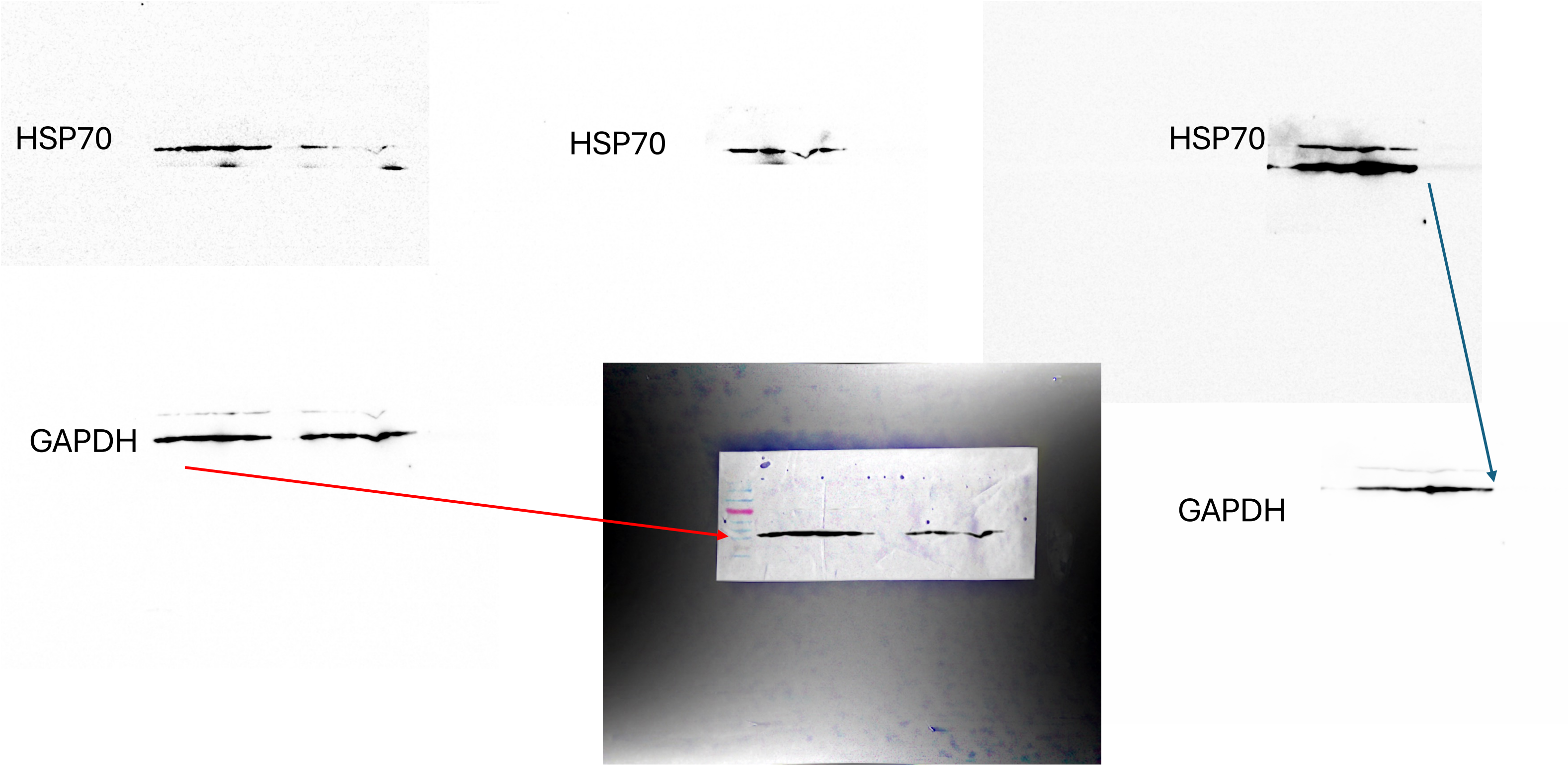

MCF-7 HSP70 day2

HSP70

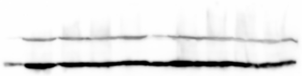

GAPDH

HSP70

GAPDH

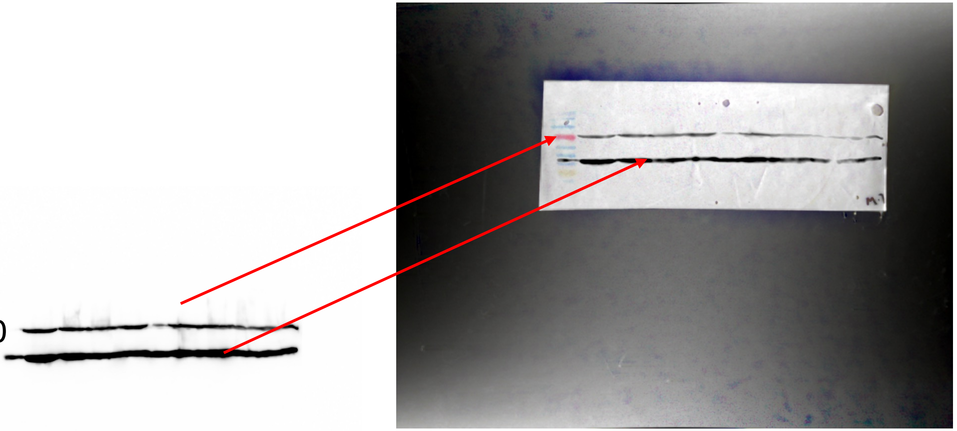

HSP70

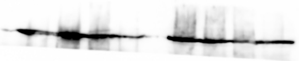

GAPDH

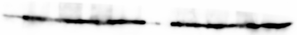

MCF-7 HSP70 day3

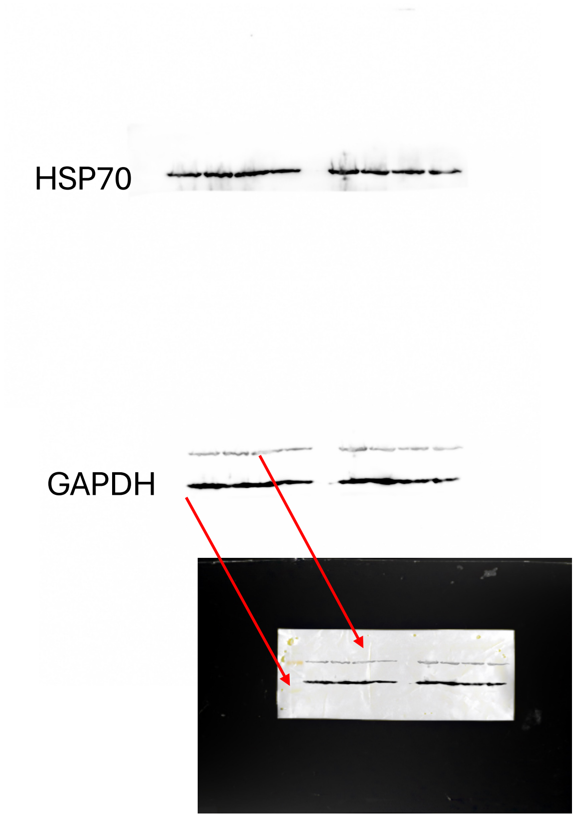

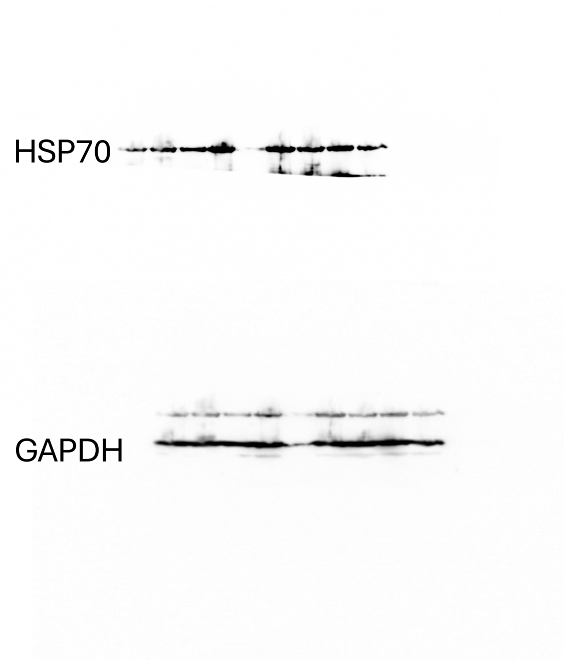

MCF-7 HSP90a day1

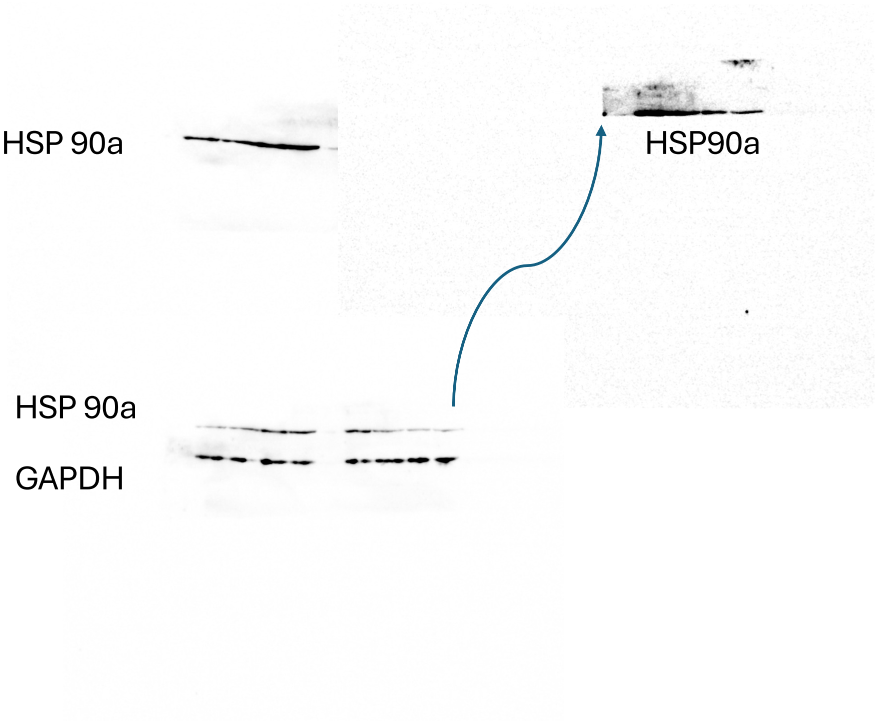

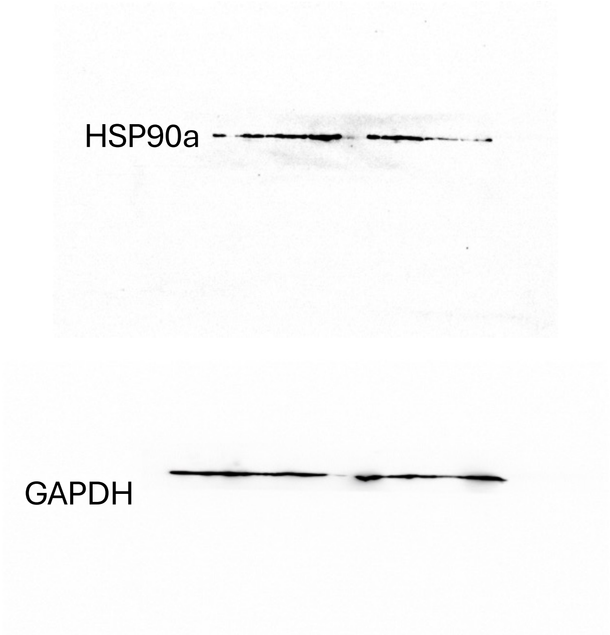

MCF-7 HSP90a day2

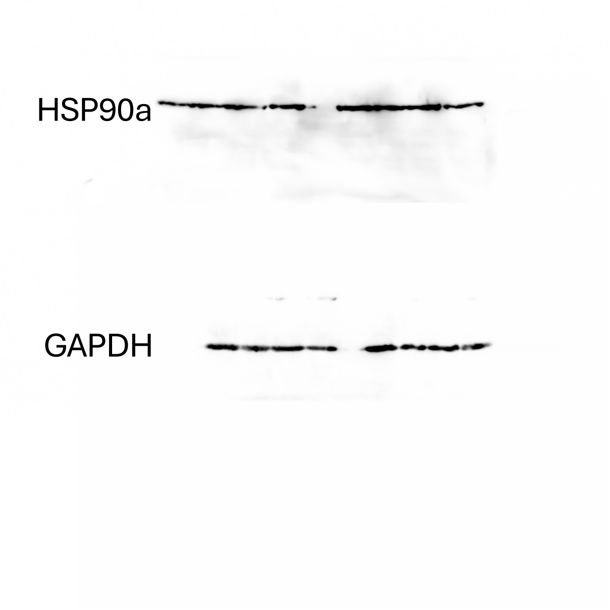

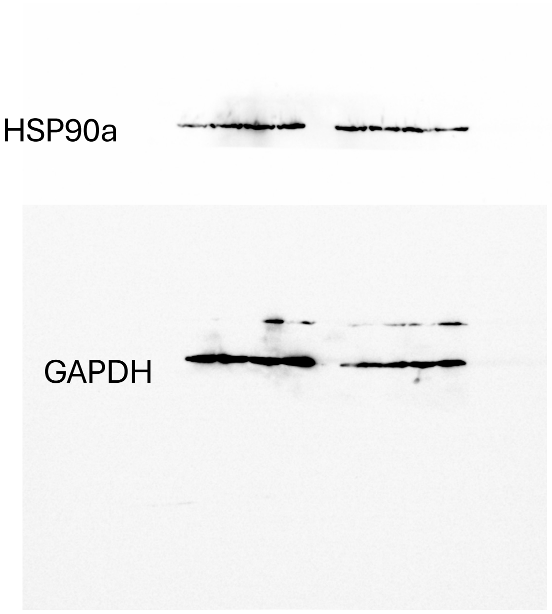

MCF-7 HSP90a day3

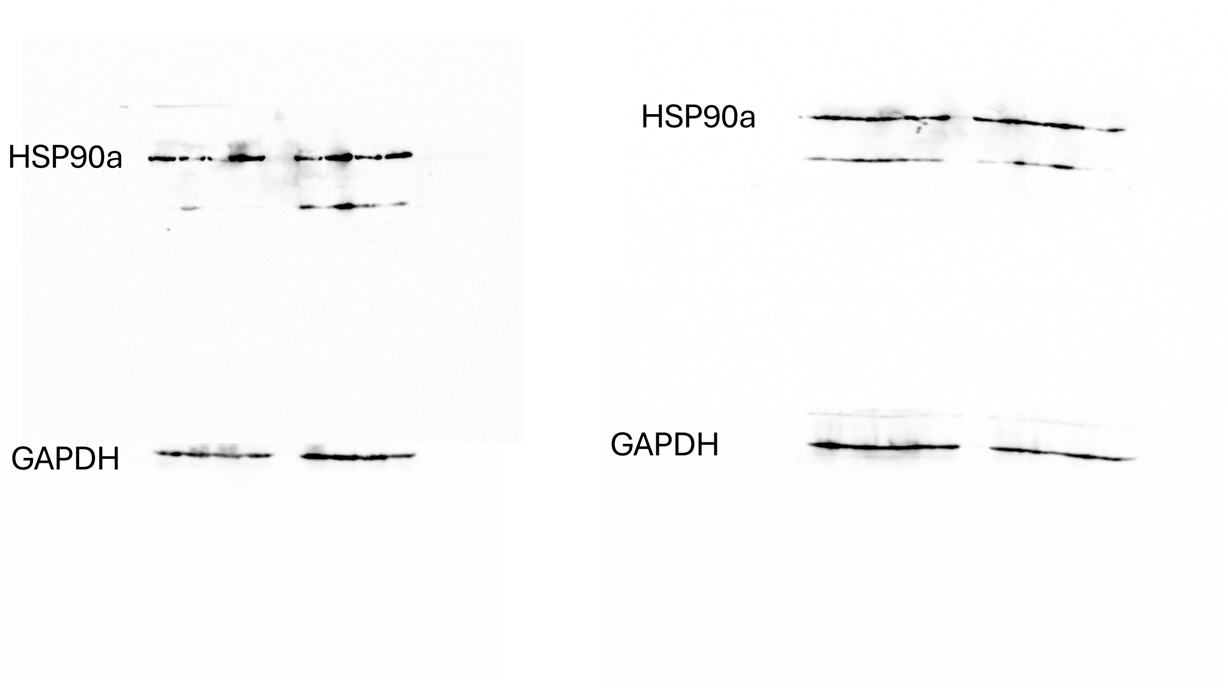

MCF-7 HSP90b day1

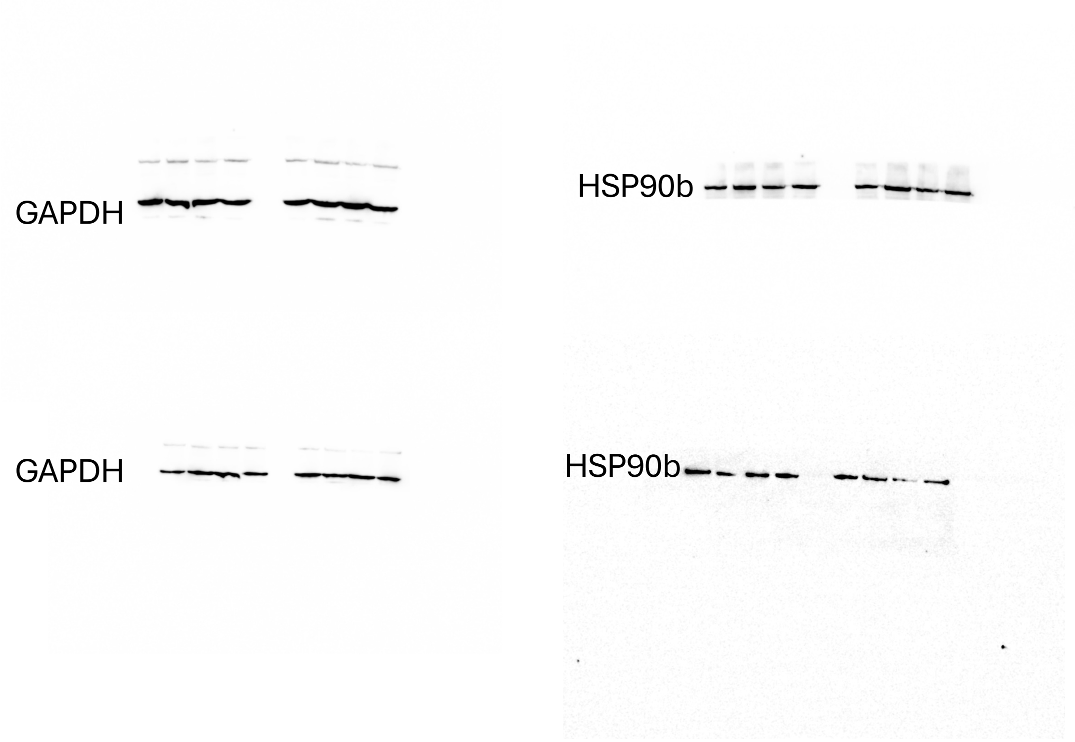

MCF-7 HSP90b day2

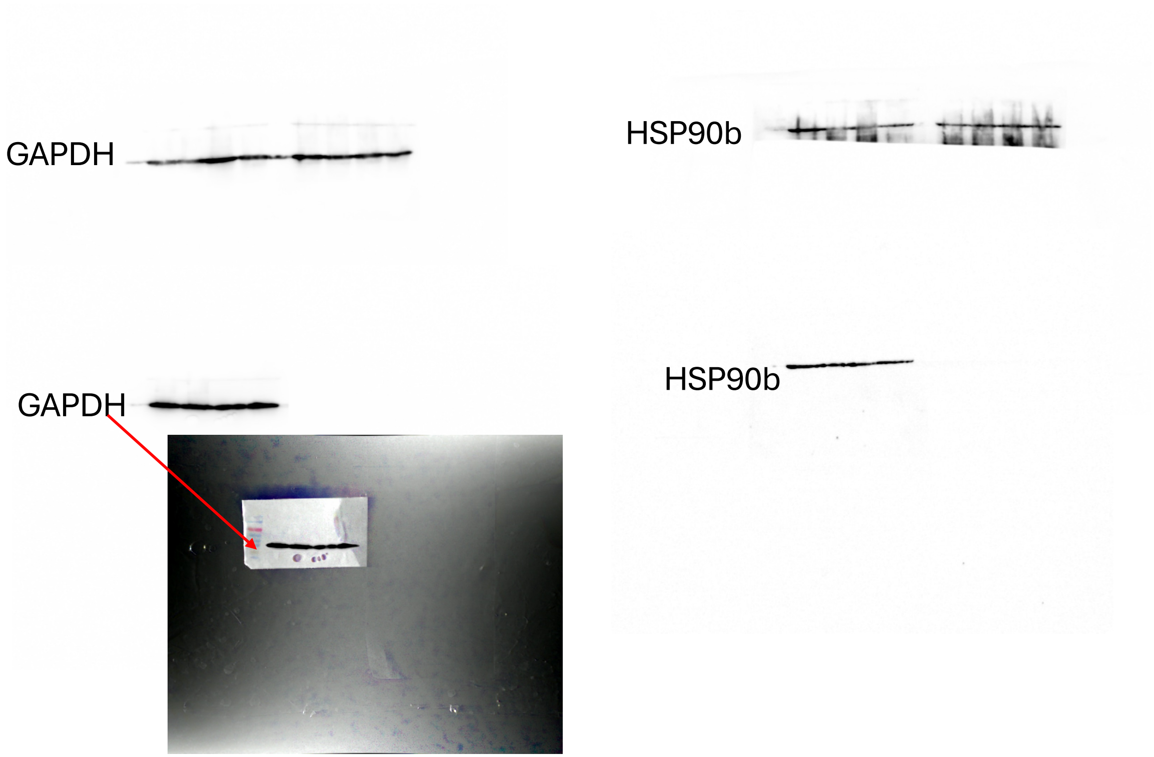

MCF-7 HSP90b day3

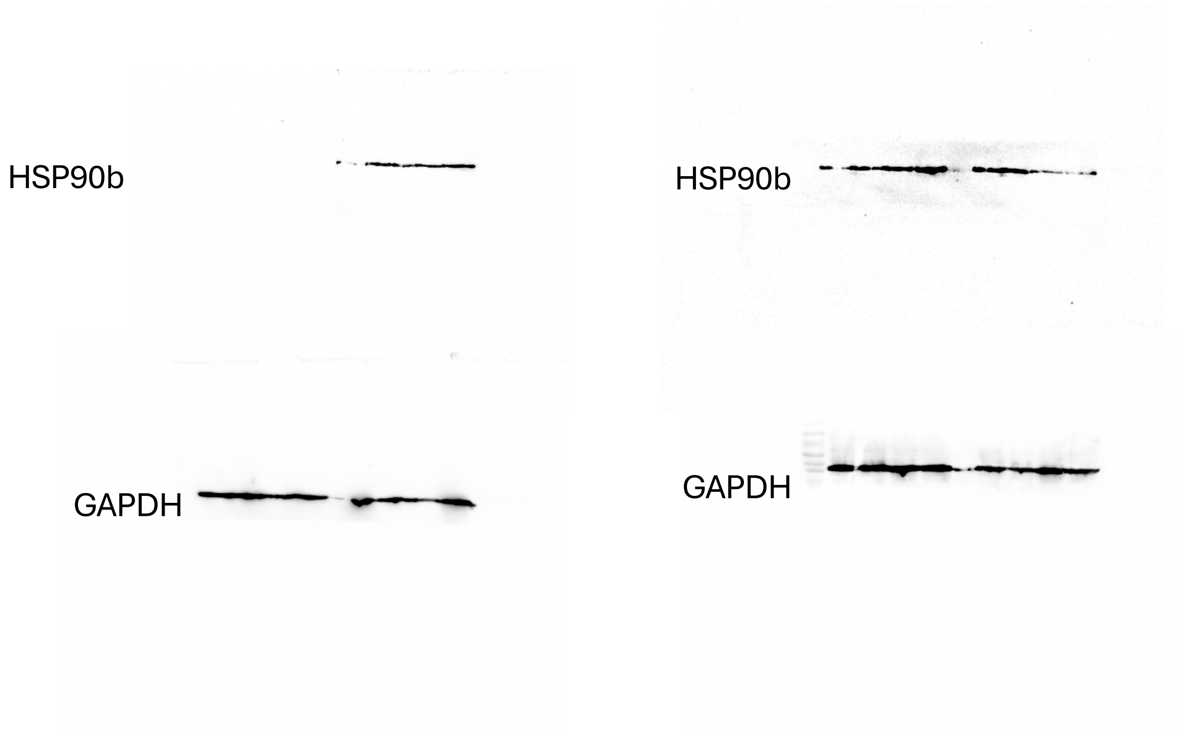

Supplement: Supplementary file 1 [file cancers-17-03517-s001.zip › cancers-3776093-supplementary/RAW BLOTS MCF-7.pdf]
