# Supplementary material for: Non-Invasive Physical Plasma as an Oncological Therapy Option: Modulation of Cancer Cell Growth, Motility, and Metabolism Without Induction of Cancer Resistance Factors
Source: Cancers (Basel). 2025 Oct 31;17(21):3517. doi: 10.3390/cancers17213517 (PMC12607350; doi:10.3390/cancers17213517)

MDA-MB-231 HSP27 day1

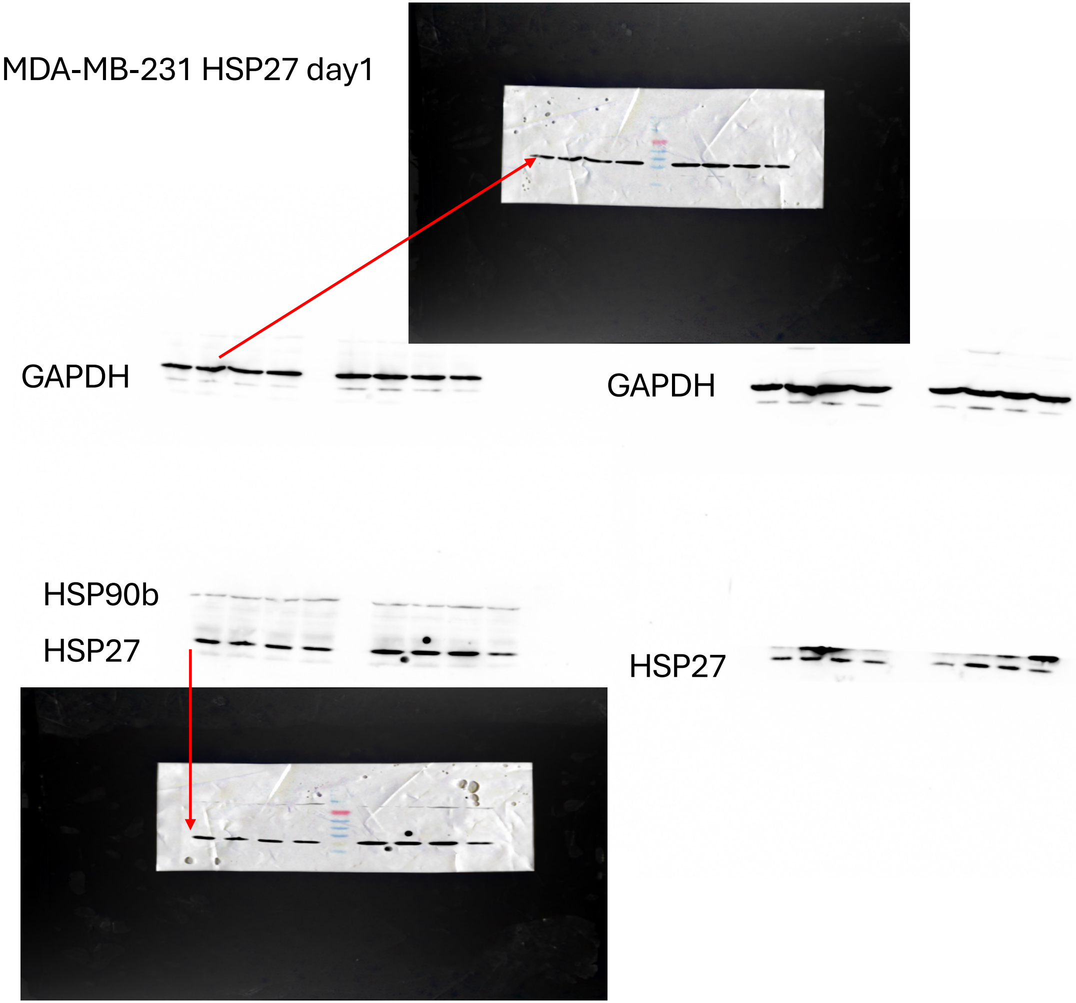

MDA-MB-231 HSP27 day2

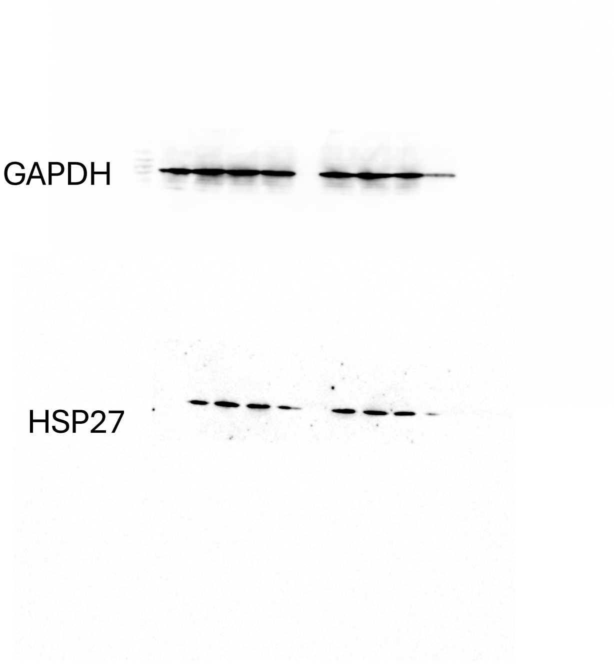


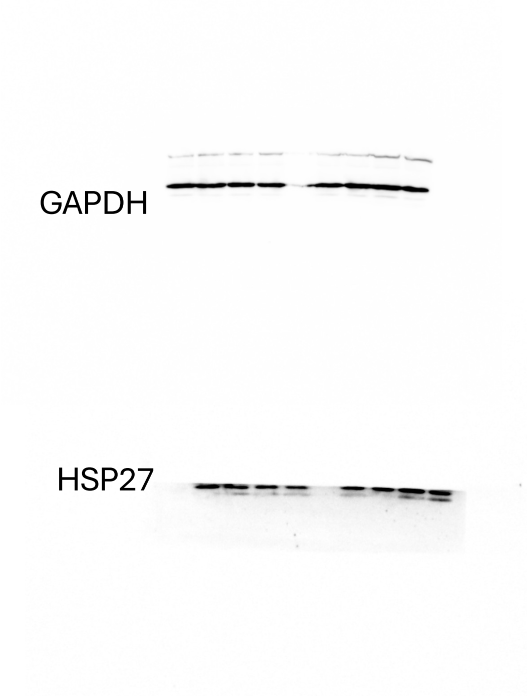

MDA-MB-231 HSP27 day3

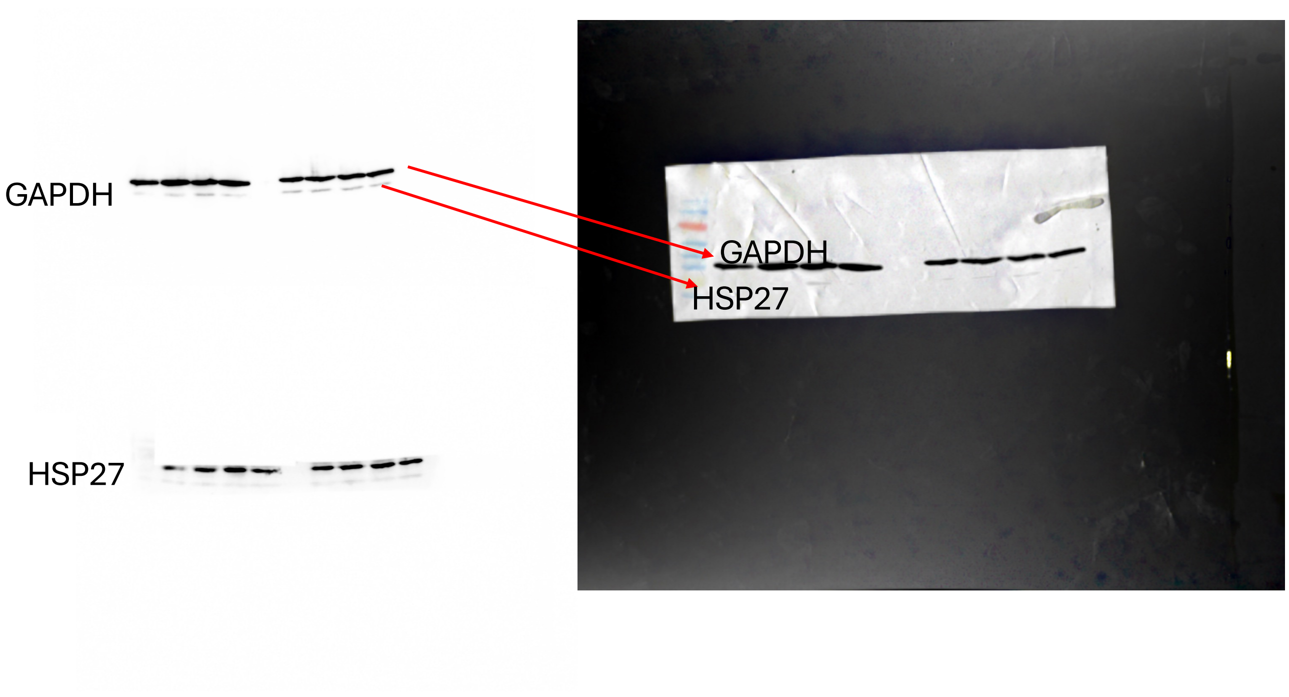

MDA-MB-231 HSP40 day1

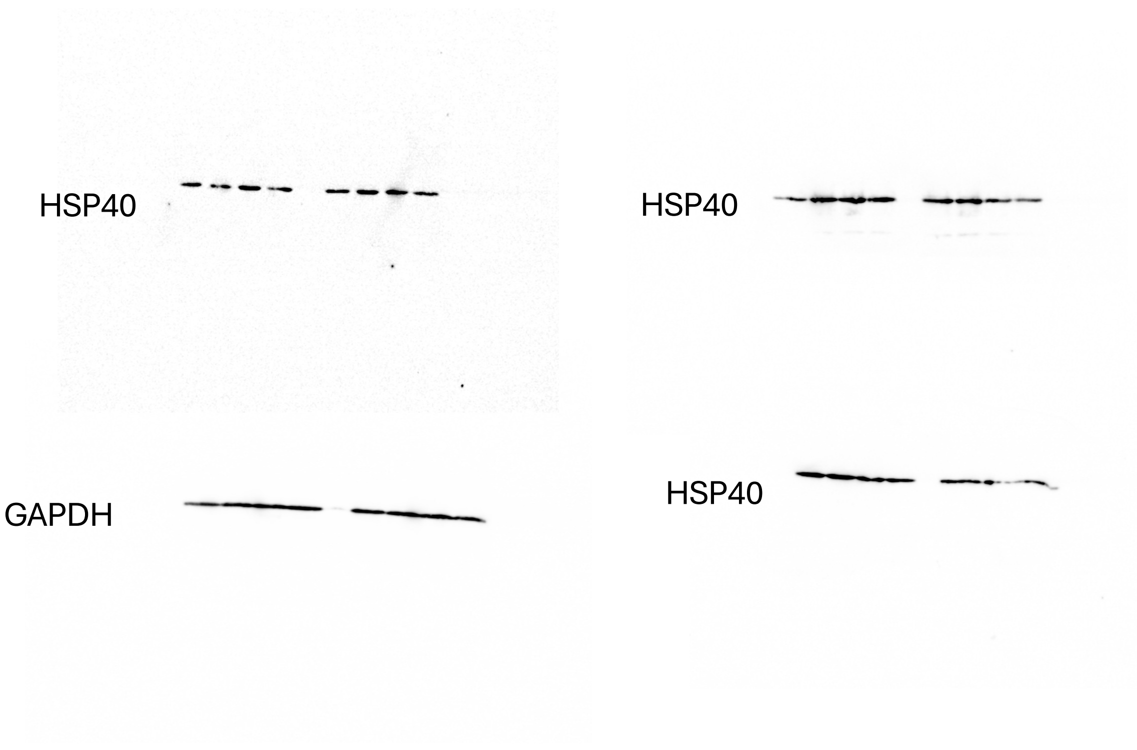

MDA-MB-231 HSP40 day2

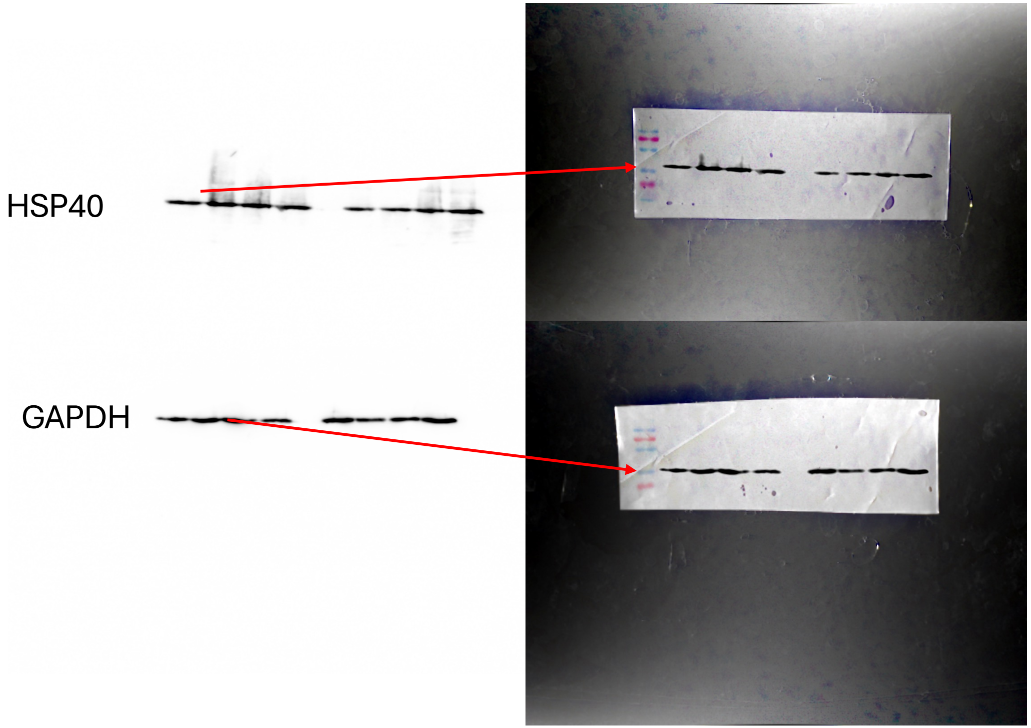

MDA-MB-231 HSP40 day3

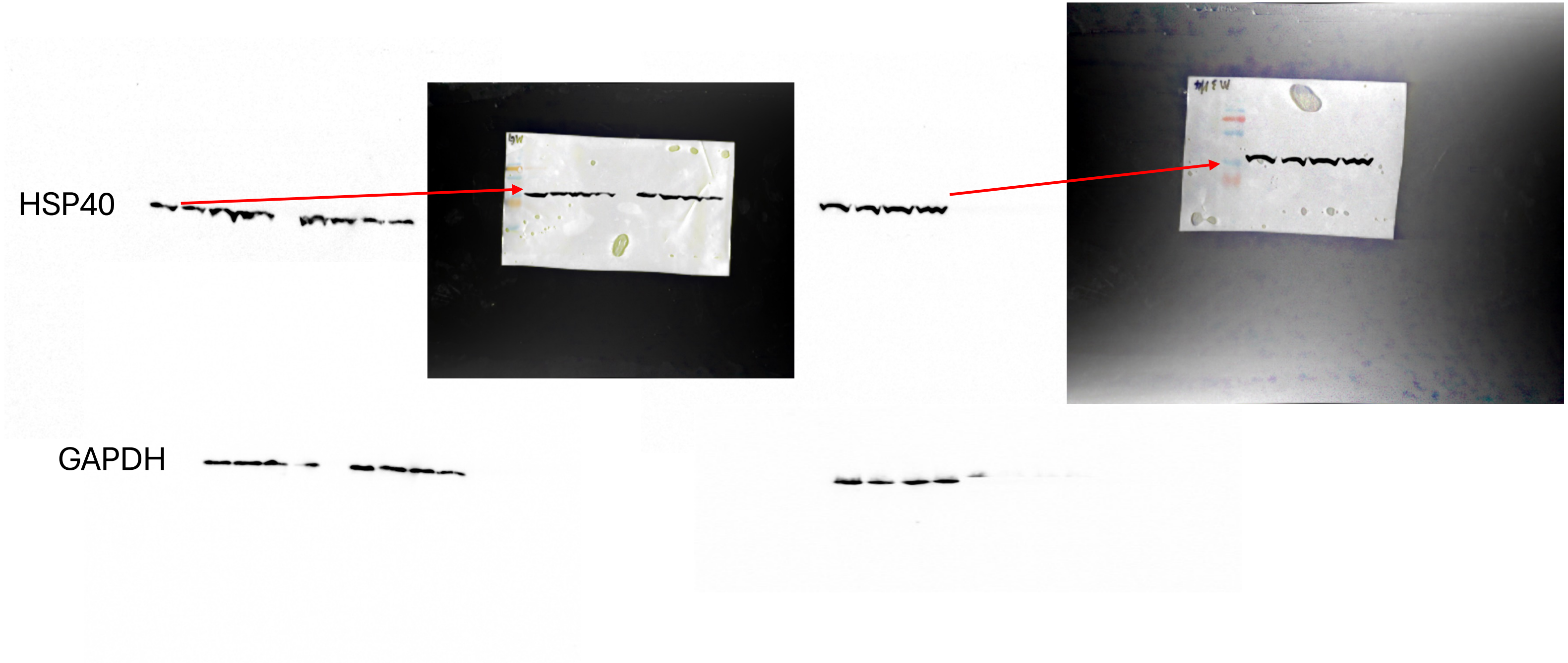

MDA-MB-231 HSP70 day1

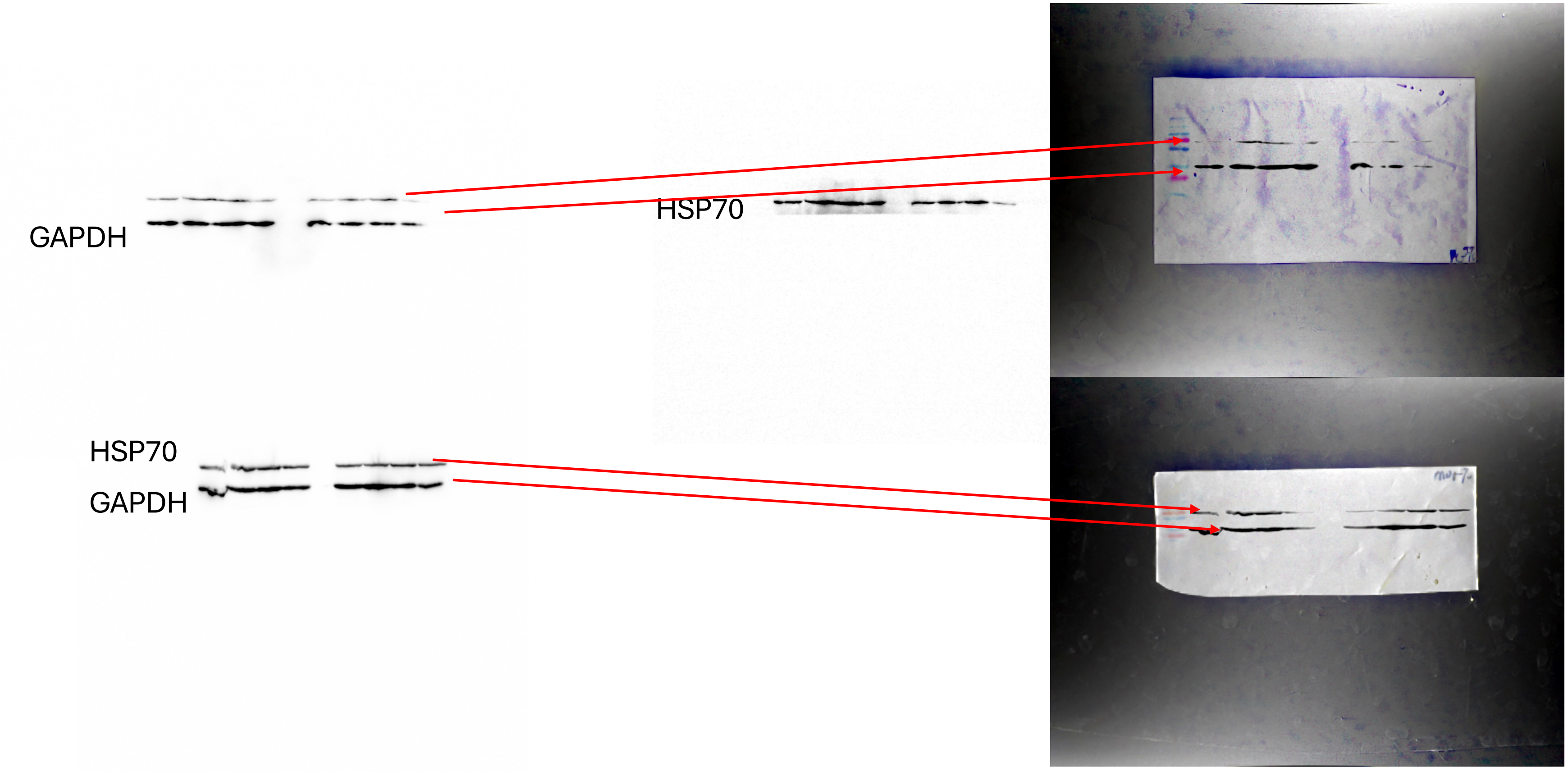

MDA-MB-231 HSP70 day2

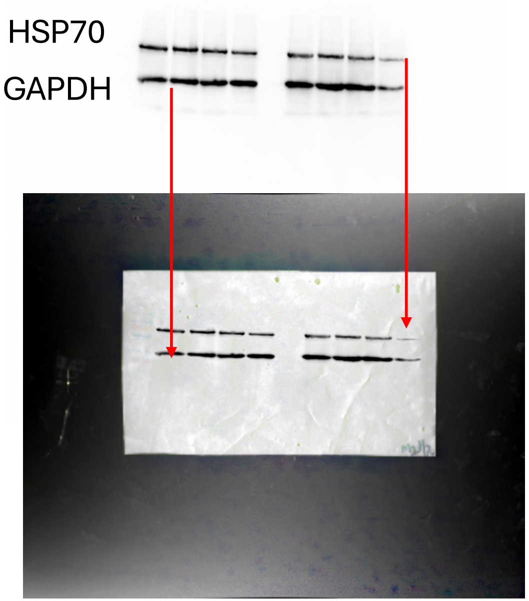


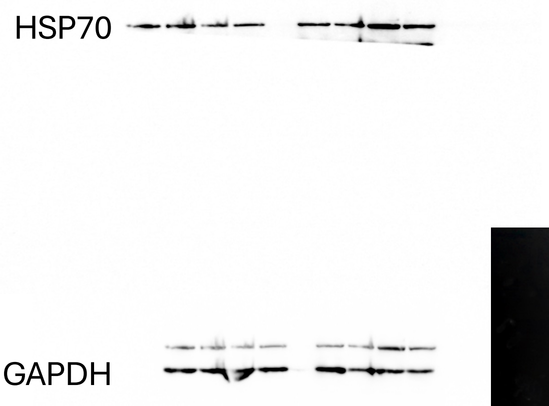


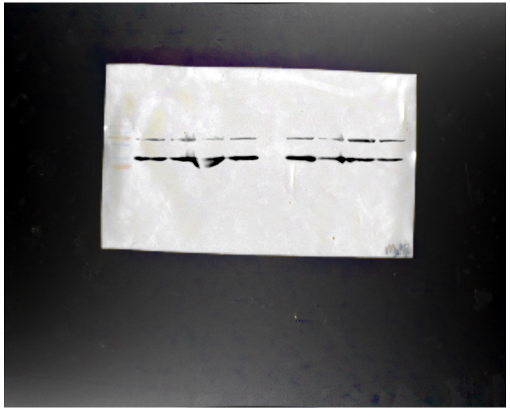

MDA-MB-231 HSP70 day3

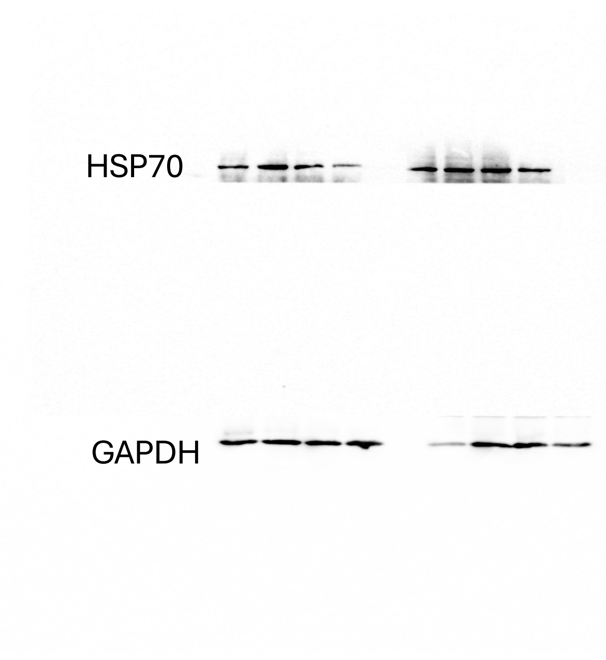


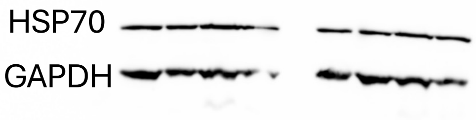


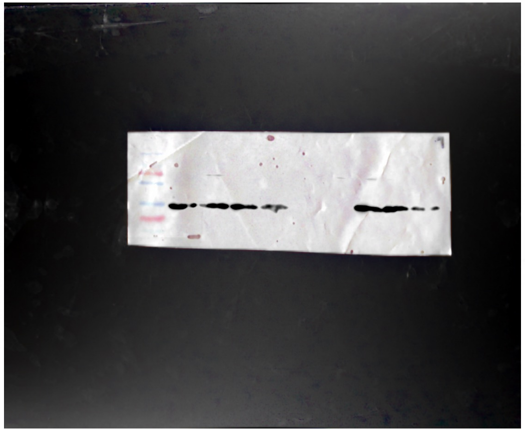

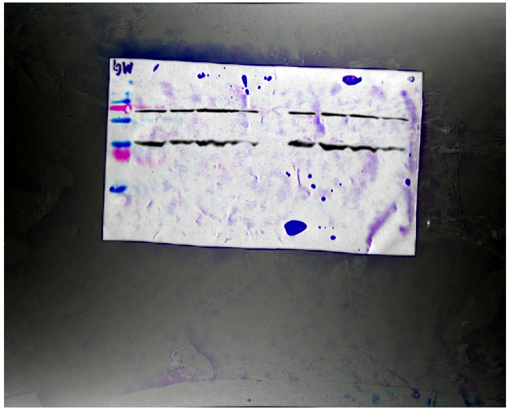

MDA-MB-231 HSP90a day1

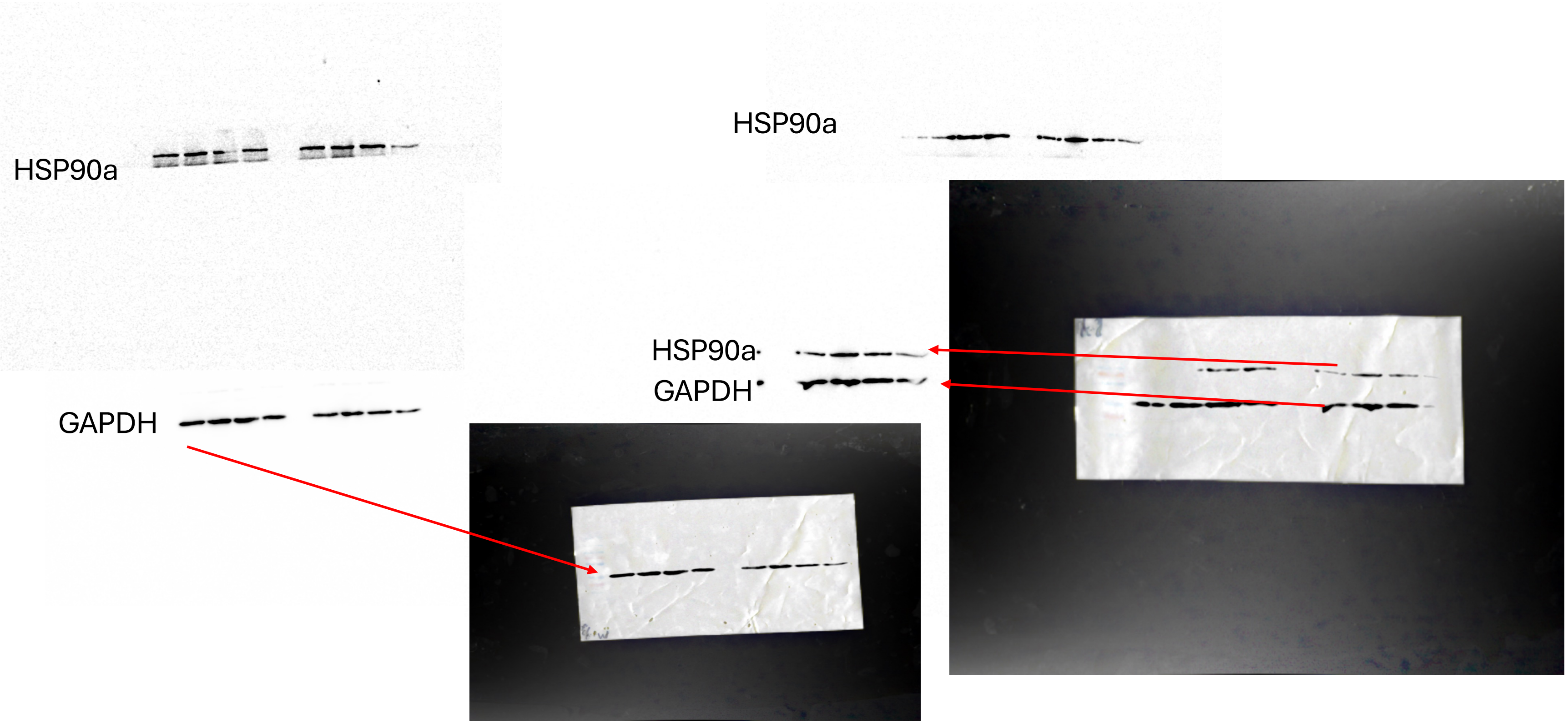

MDA-MB-231 HSP90a day2

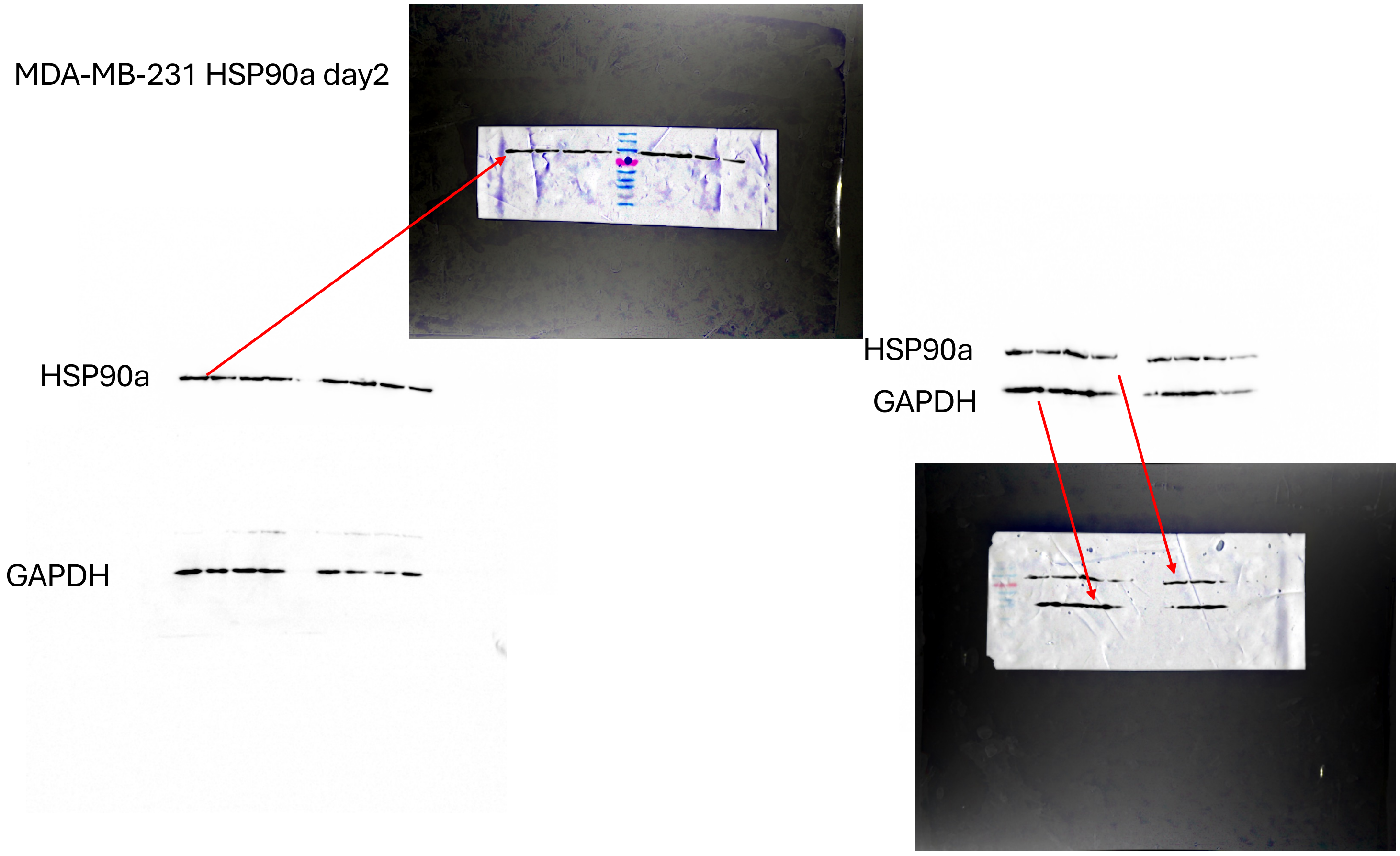

MDA-MB-231 HSP90a day3

HSP90a

GAPDH

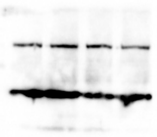

GAPDH

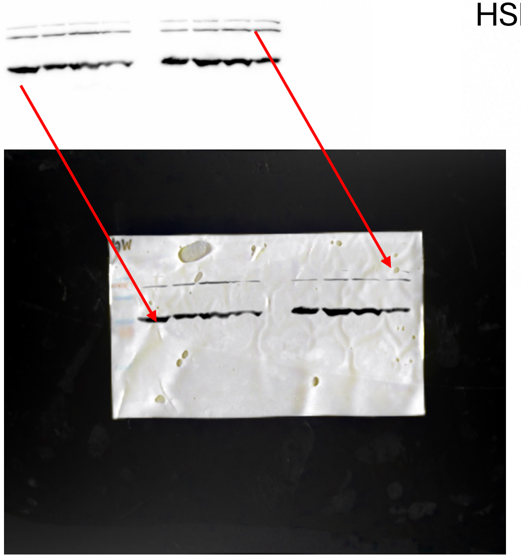

HSP90a

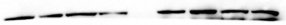

MDA-MB-231 HSP90b day1

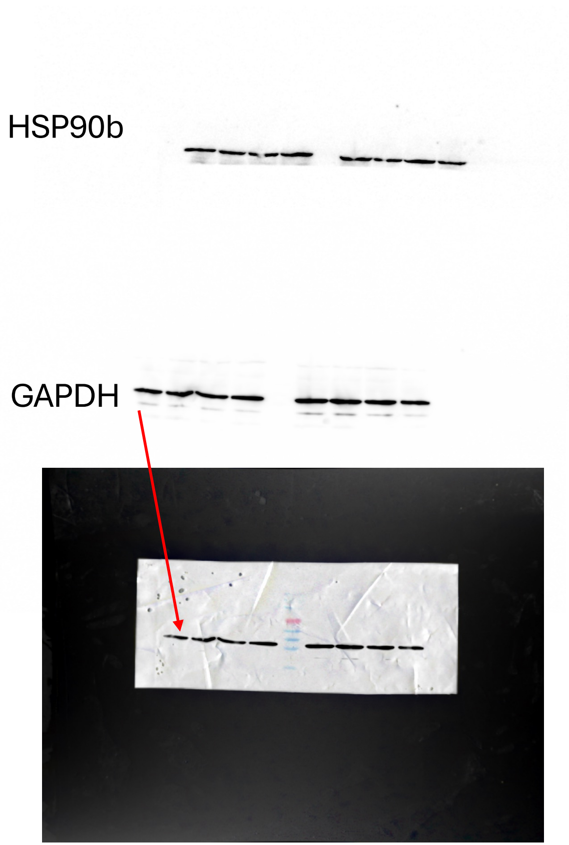


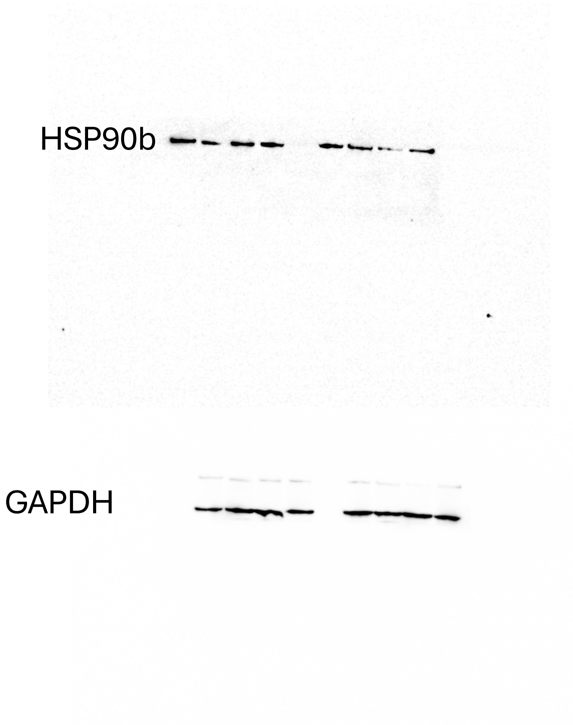

MDA-MB-231 HSP90b day2

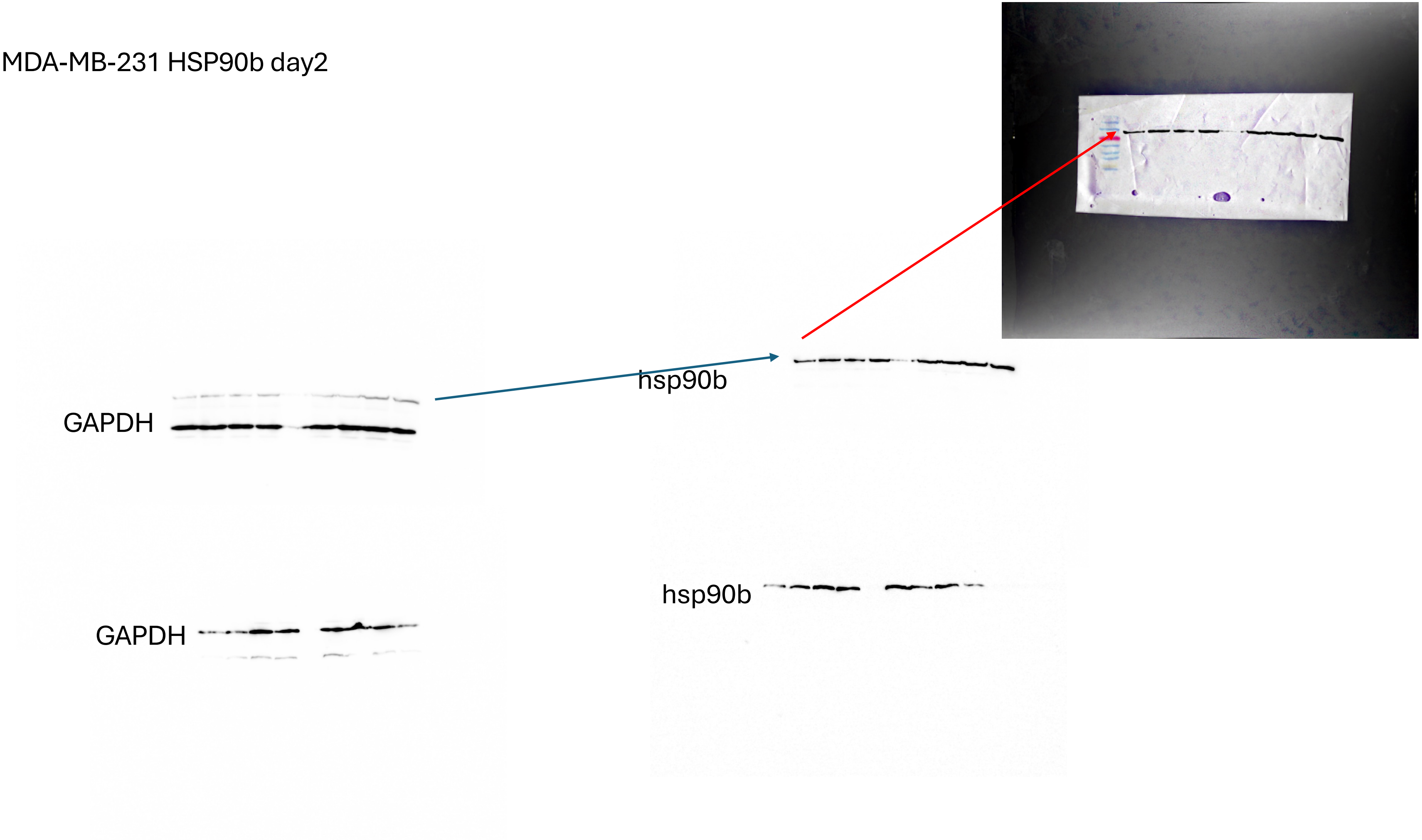

MDA-MB-231 HSP90b day3

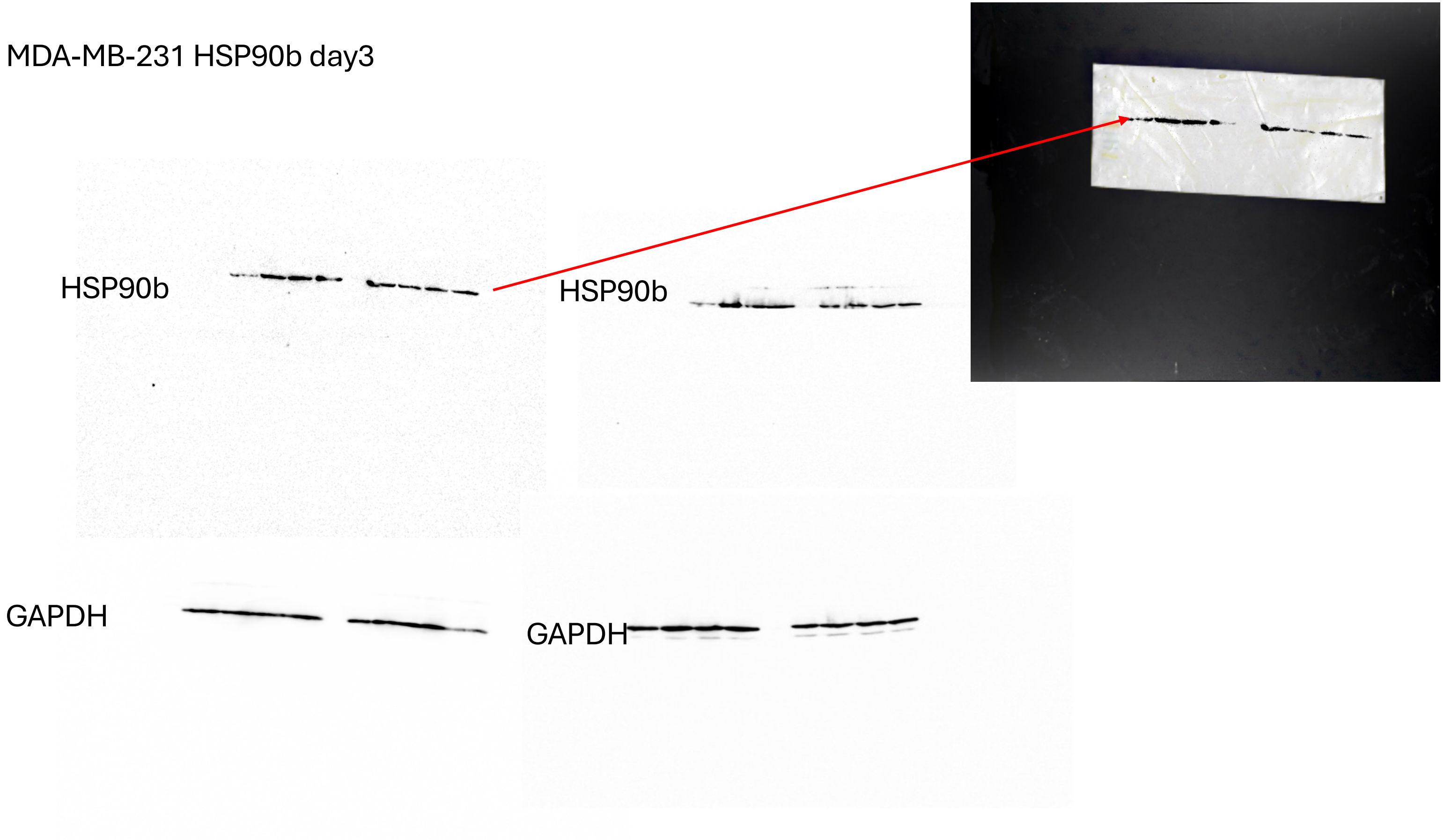

Supplement: Supplementary file 1 [file cancers-17-03517-s001.zip › cancers-3776093-supplementary/RAW BLOTS MDA-MB-231.pdf]
