# Supplementary material for: Non-Invasive Physical Plasma as an Oncological Therapy Option: Modulation of Cancer Cell Growth, Motility, and Metabolism Without Induction of Cancer Resistance Factors
Source: Cancers (Basel). 2025 Oct 31;17(21):3517. doi: 10.3390/cancers17213517 (PMC12607350; doi:10.3390/cancers17213517)

OVCAR3 HSP27   day1

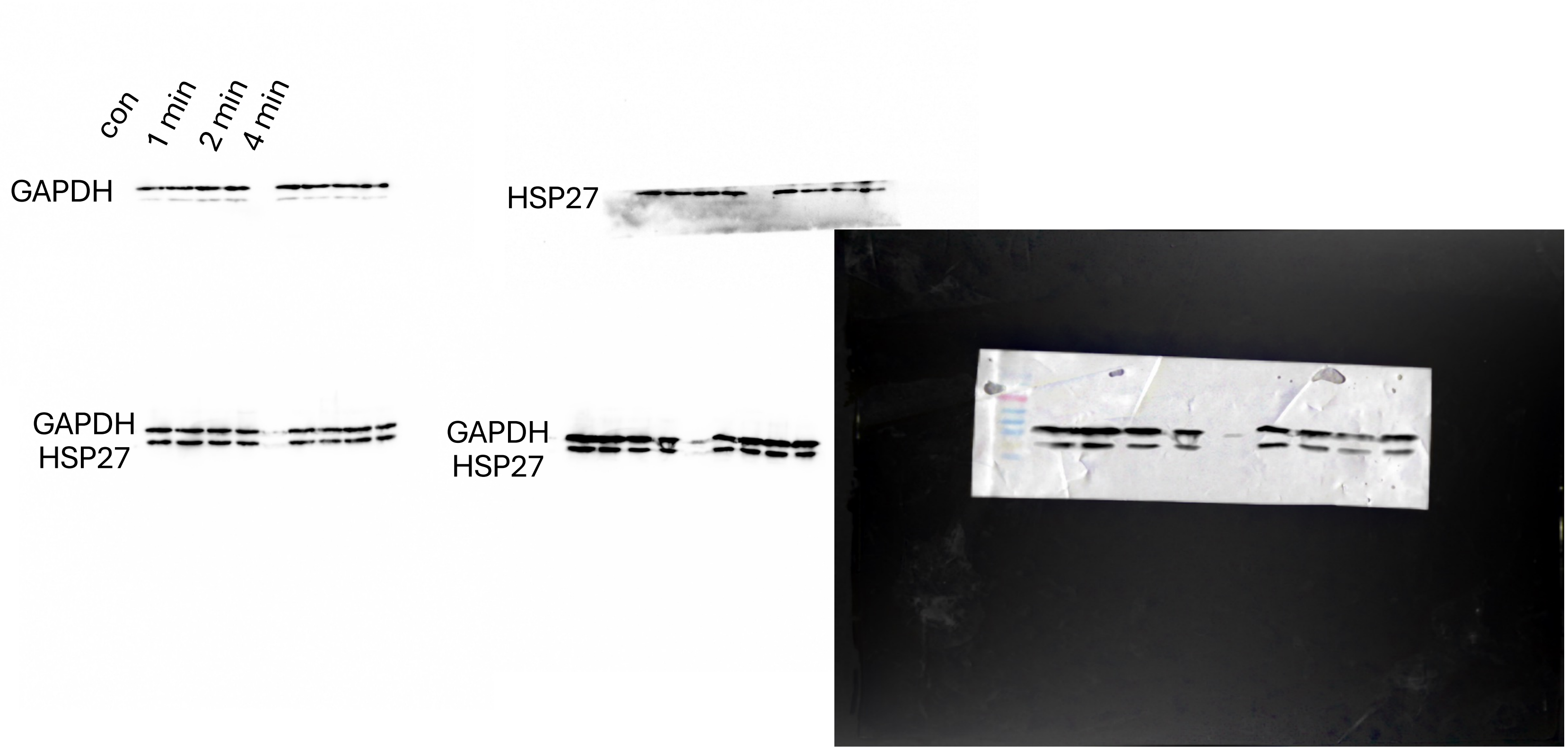

OVCAR3 HSP27 day2

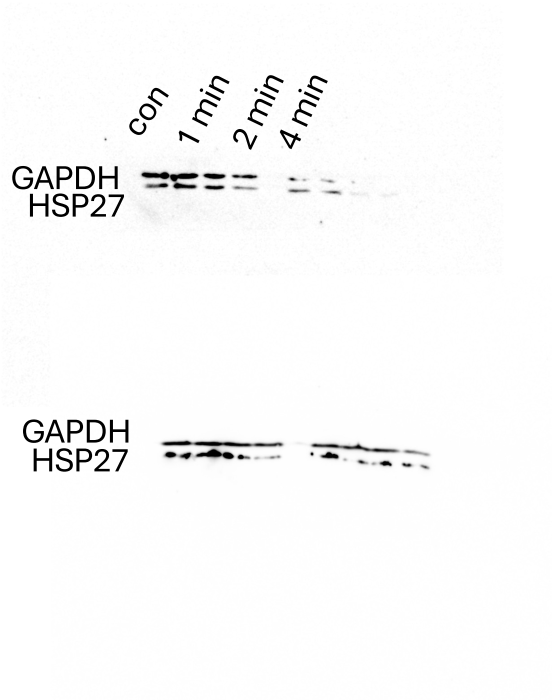

OVCAR3 HSP27 day3

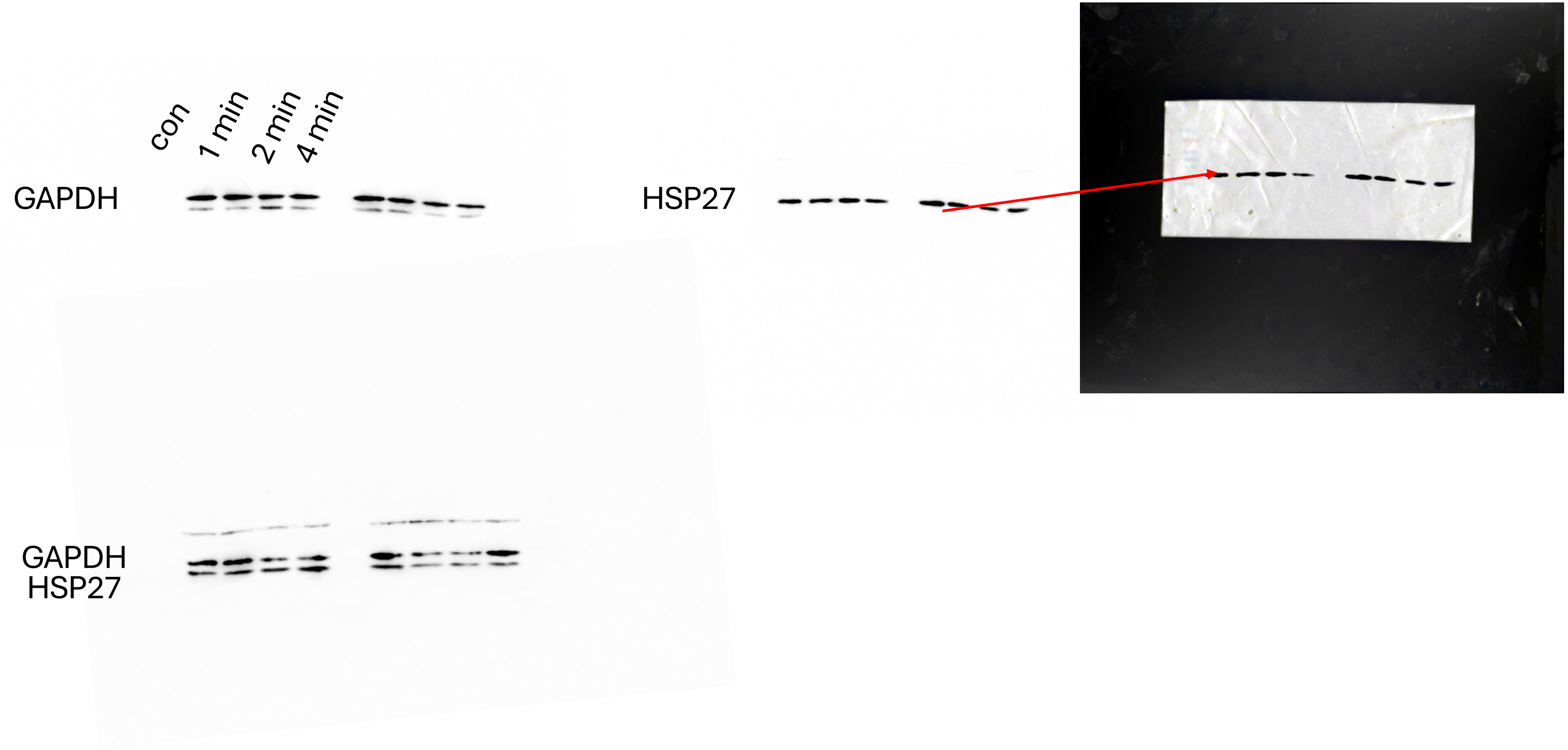

OVCAR3 HSP40   day1

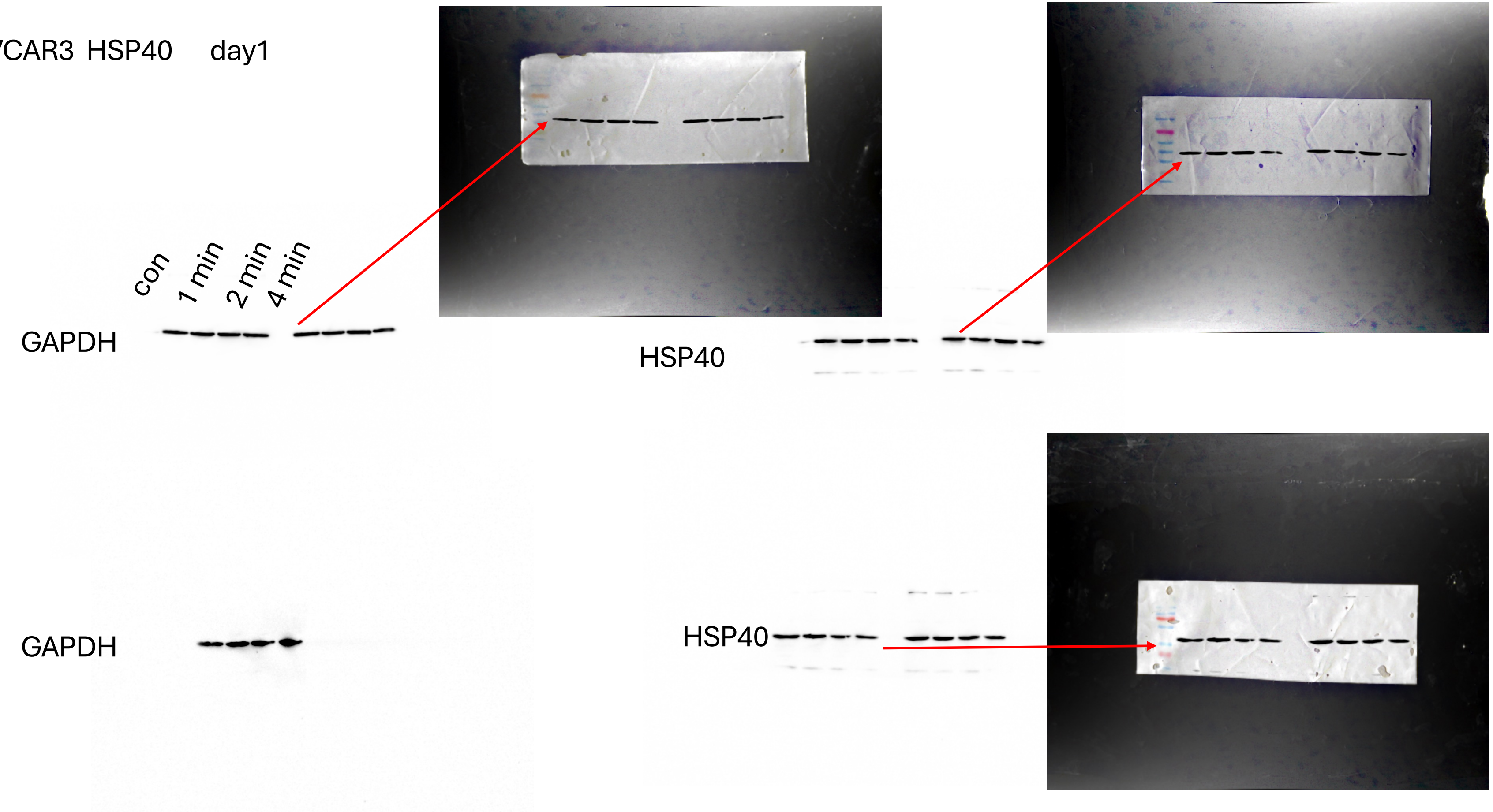

OVCAR3 HSP40 day2

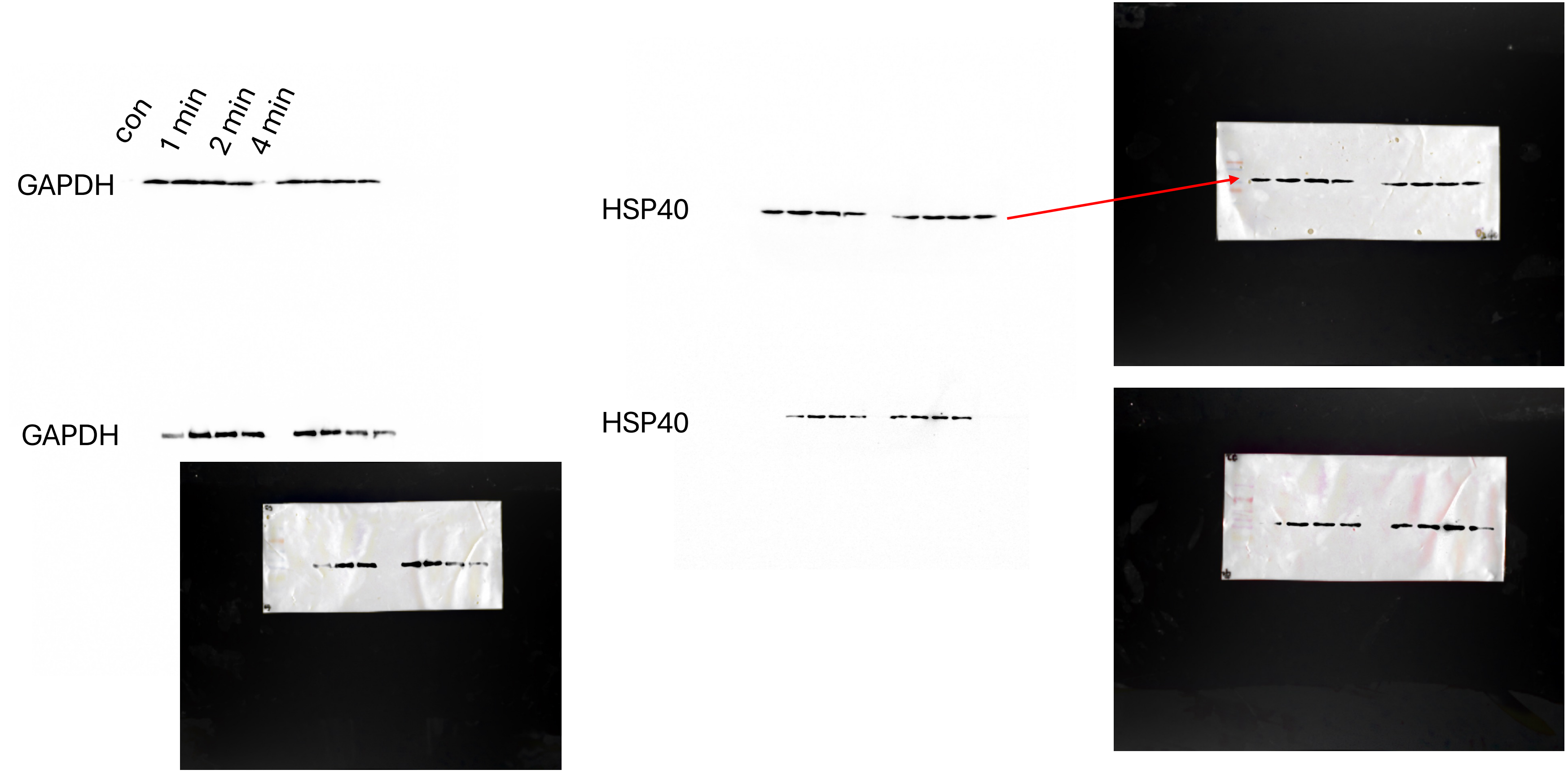

OVCAR3 HSP40 day3

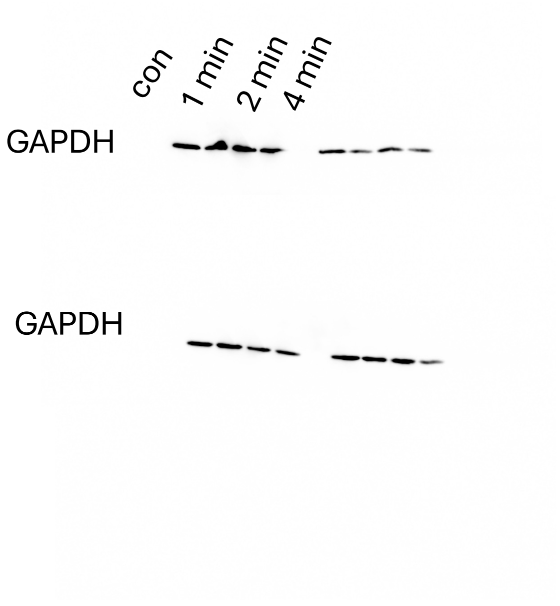

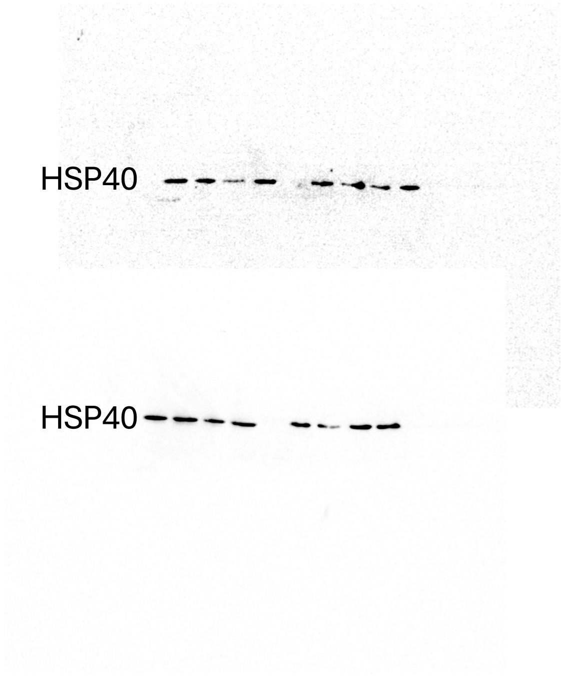

OVCAR3 HSP70 Day 1

con 1 min 2 min 4 min

GAPDH

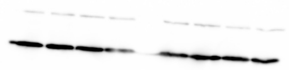

HSP70

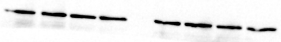

HSP70

GAPDH

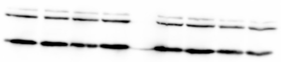

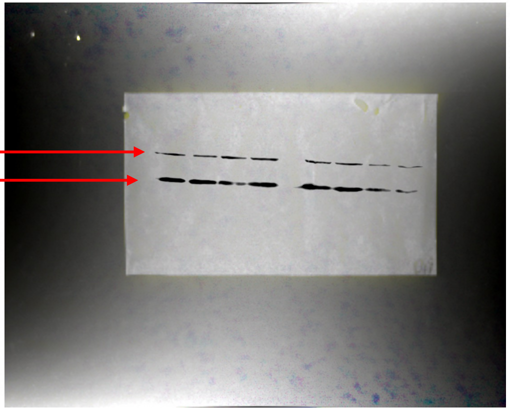

OVCAR3 HSP70 Day2

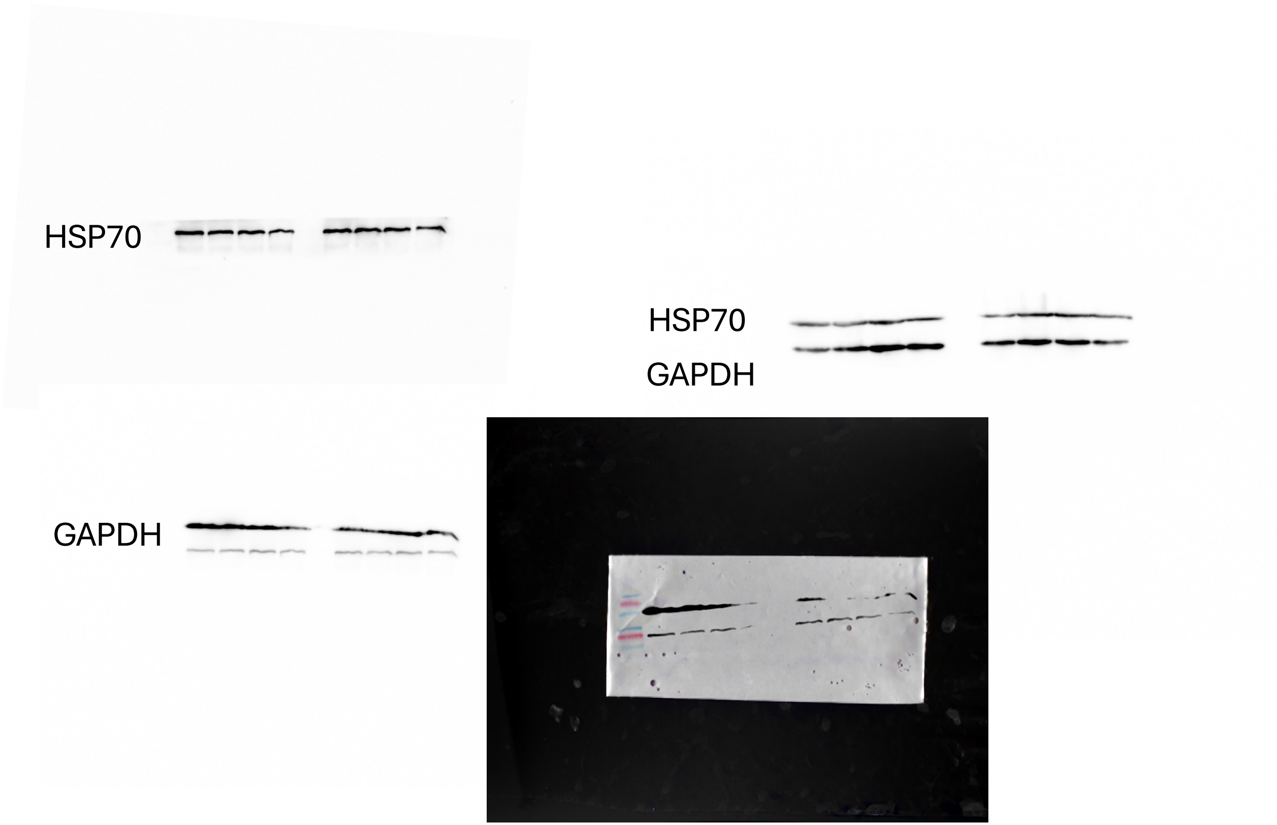

OVCAR3 HSP70 Day3

GAPDH

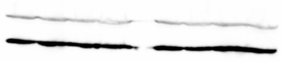

HSP70

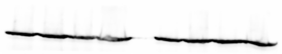

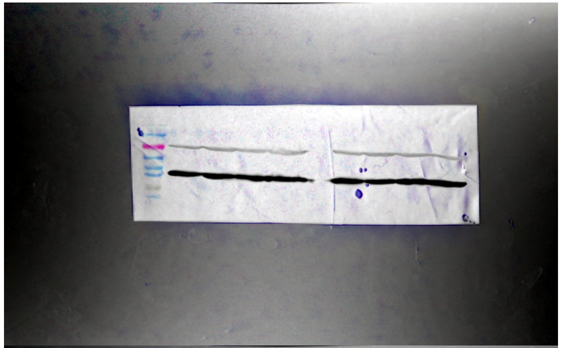

GAPDH

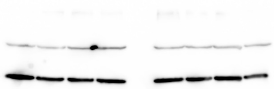

HSP70

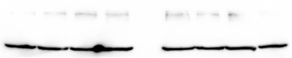

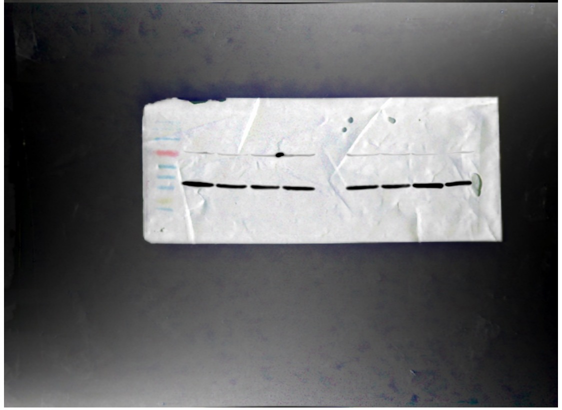

OVCAR3 HSP90a Day1

GAPDH

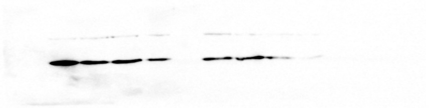

HSP90a

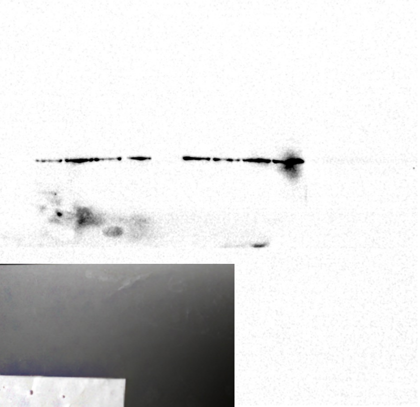

HSP90a

GAPDH

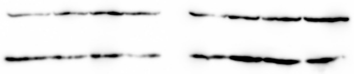

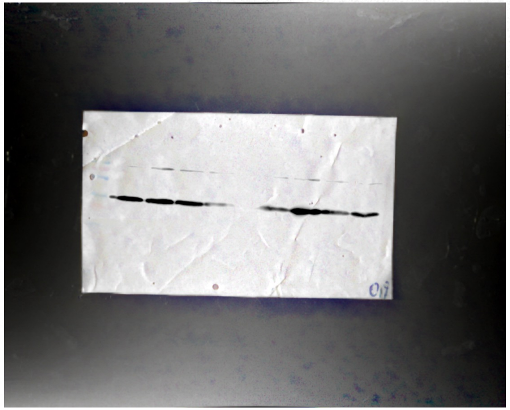

OVCAR3 HSP90a   Day2

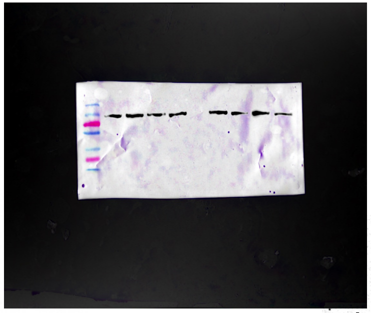

HSP90a

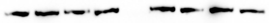

HSP90a

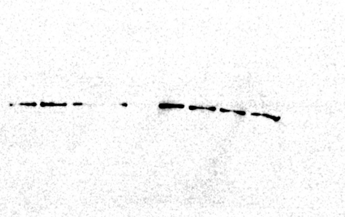

GAPDH

GAPDH

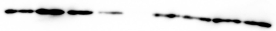

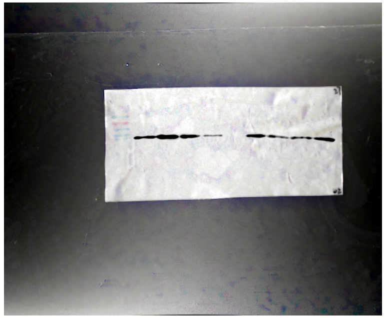

OVCAR3 HSP90a Day3

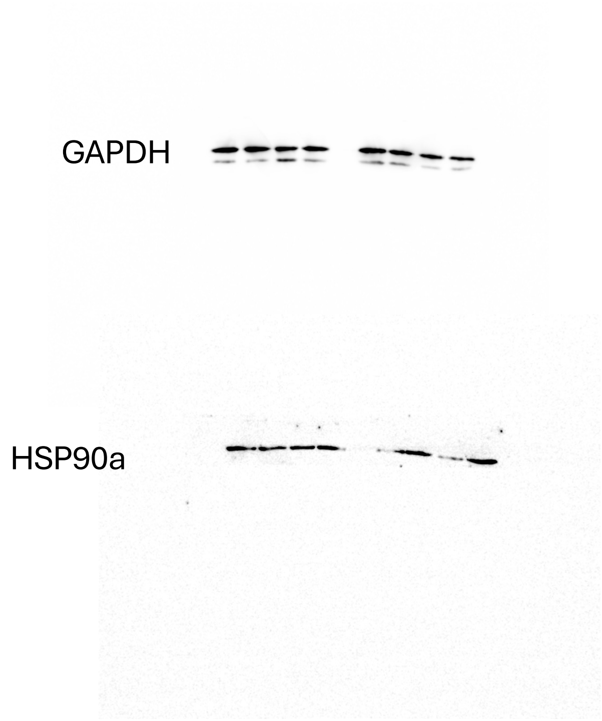

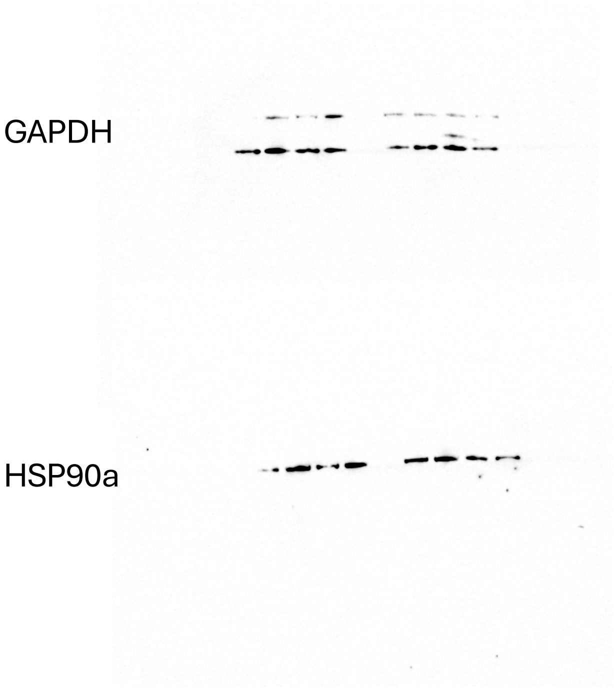

OVCAR3 HSP90b Day1

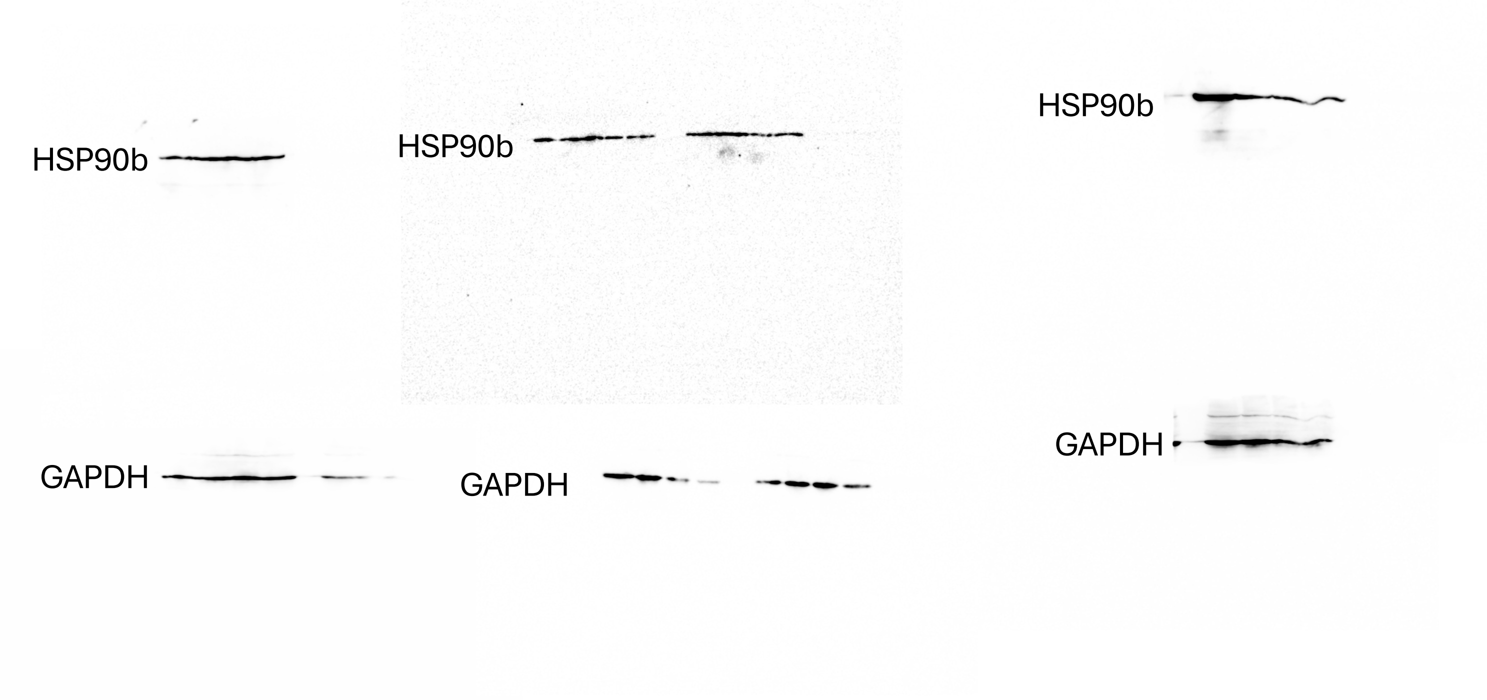

OVCAR3 HSP90b Day2

GAPDH

GAPDH

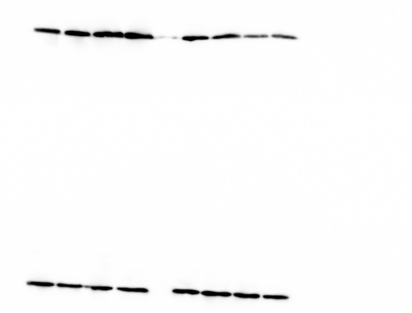

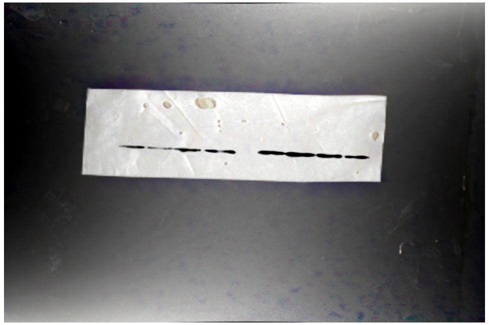

HSP90b

HSP90b

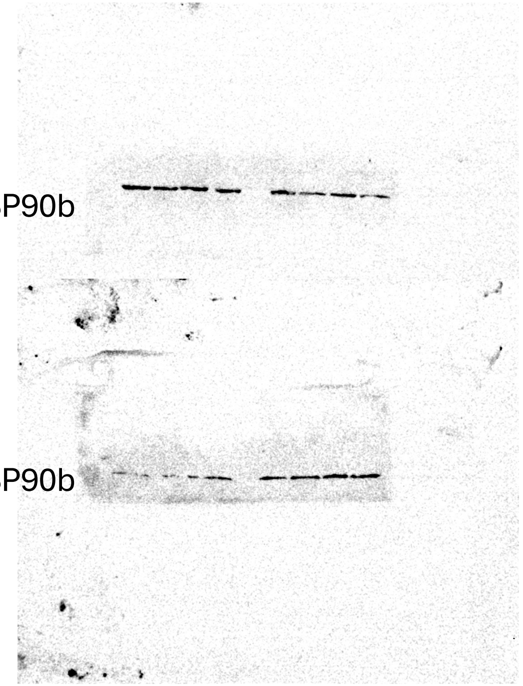

OVCAR3 HSP90b Day3

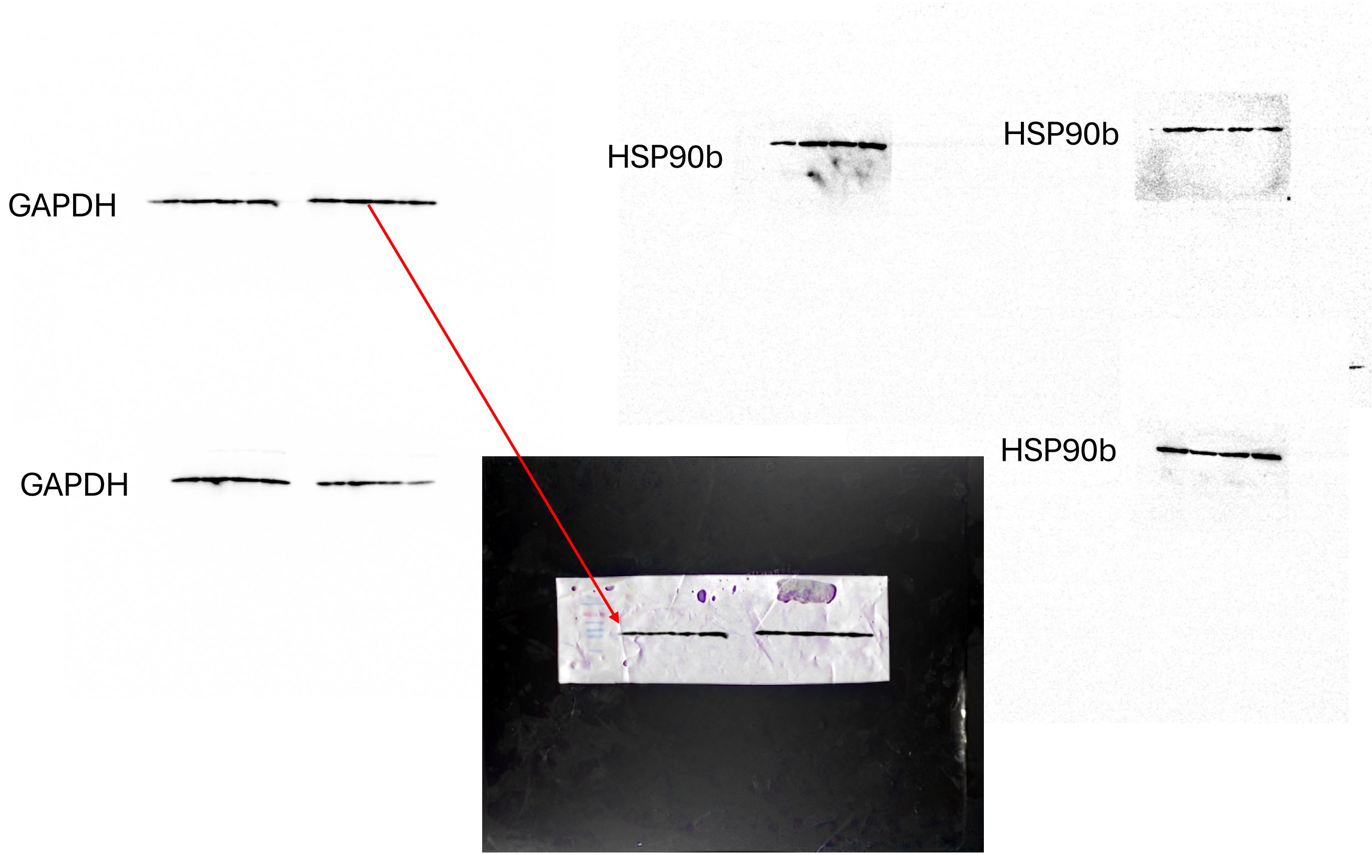

Supplement: Supplementary file 1 [file cancers-17-03517-s001.zip › cancers-3776093-supplementary/RAW BLOTS OVCAR-3.pdf]
