# Supplementary material for: Non-Invasive Physical Plasma as an Oncological Therapy Option: Modulation of Cancer Cell Growth, Motility, and Metabolism Without Induction of Cancer Resistance Factors
Source: Cancers (Basel). 2025 Oct 31;17(21):3517. doi: 10.3390/cancers17213517 (PMC12607350; doi:10.3390/cancers17213517)

PC3 HSP27 day1

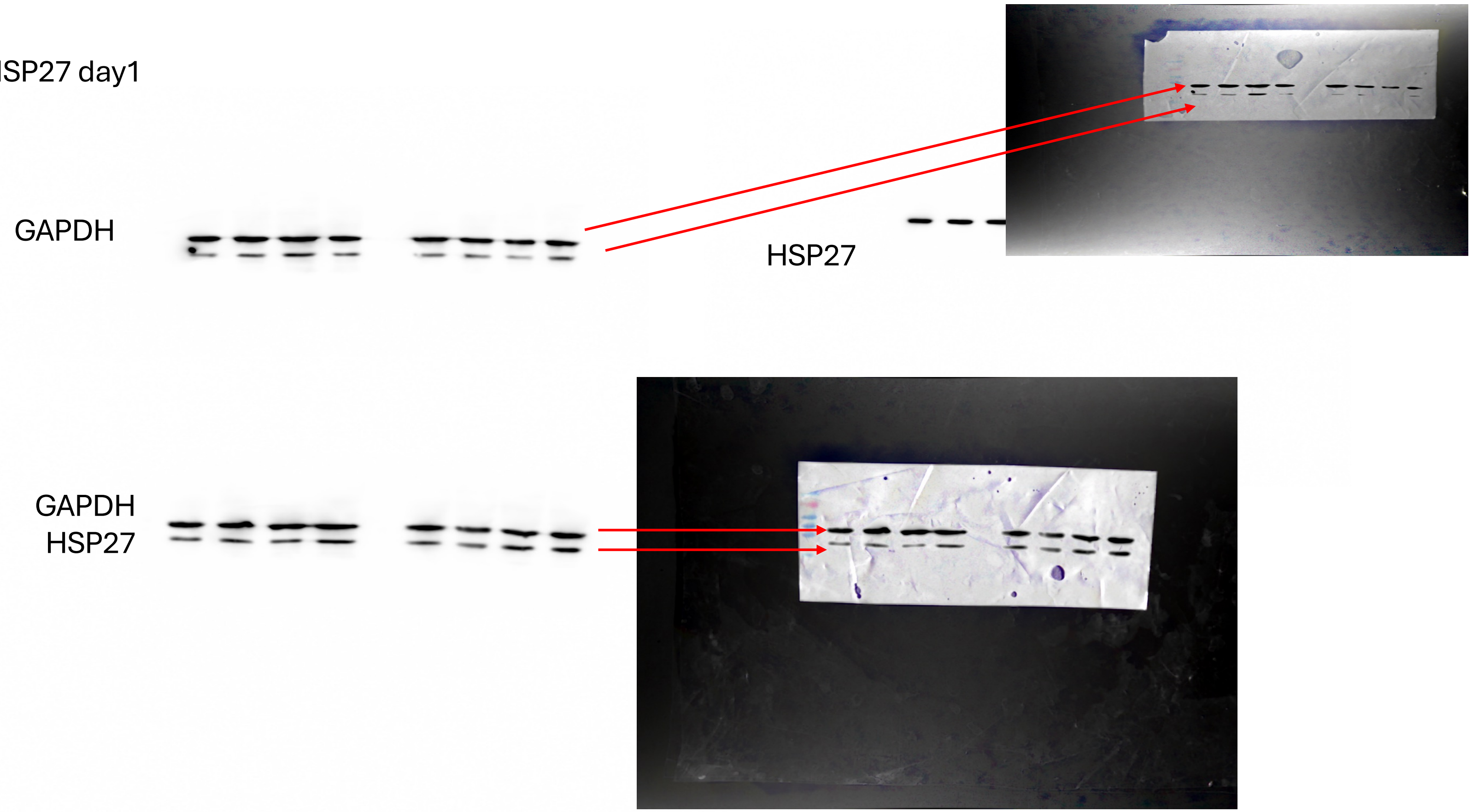

PC3 HSP27 day2

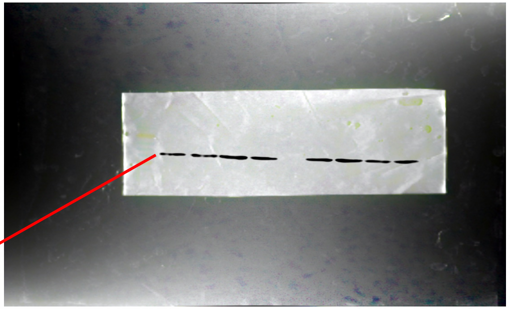

GAPDH

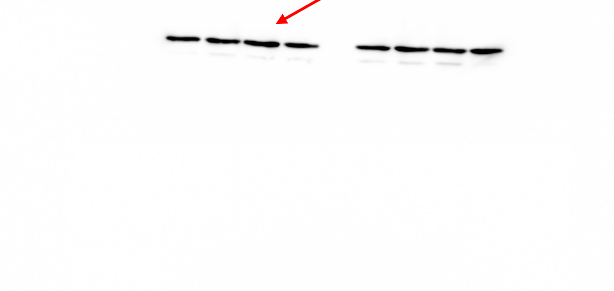

HSP27

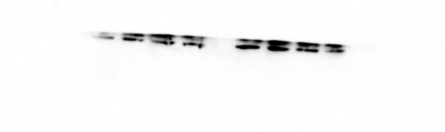

GAPDH

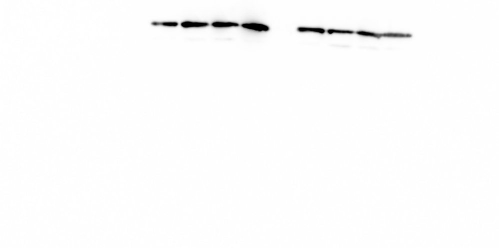

HSP27

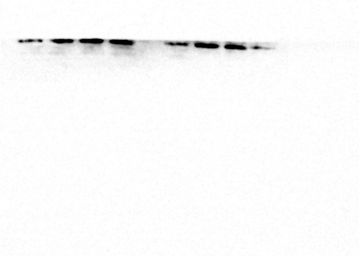

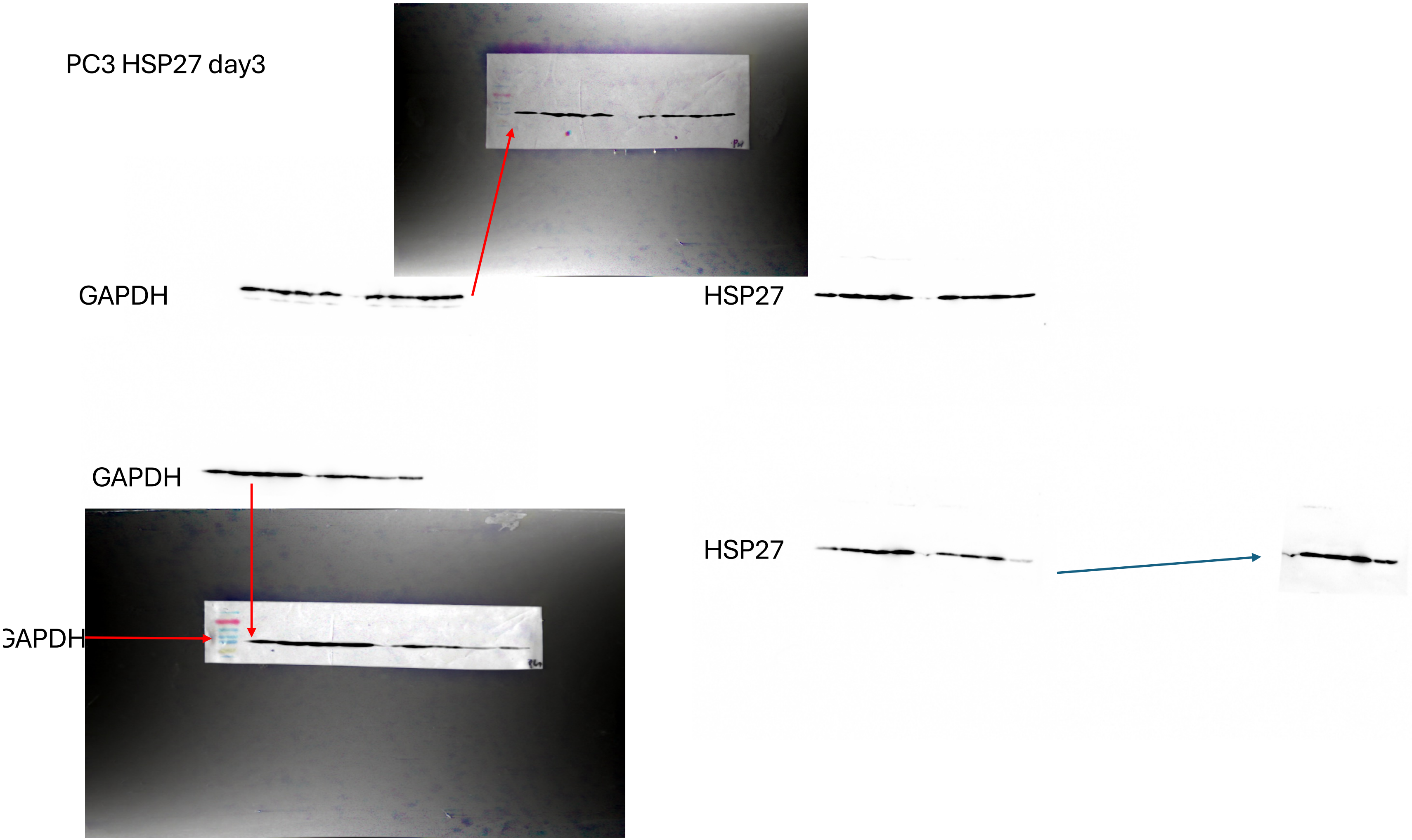
PC3 HSP27 day3
GAPDH
HSP27
GAPDH
HSP27
GAPDH

PC3 HSP40 day1

GAPDH

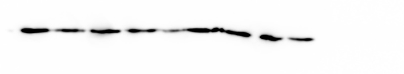

HSP40

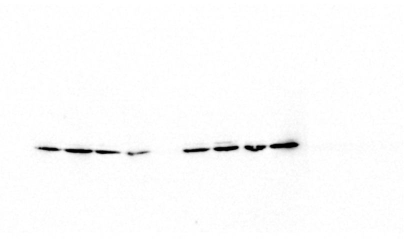

HSP40

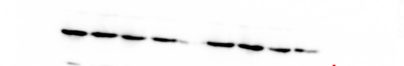

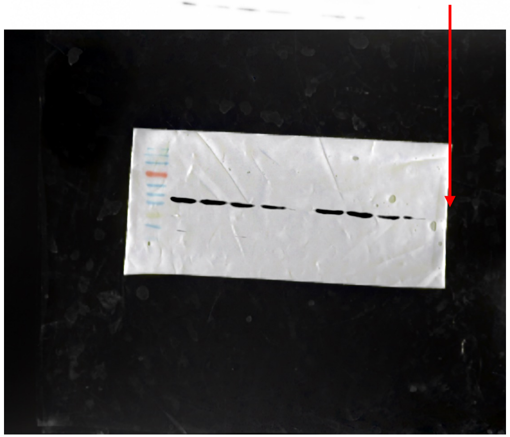

GAPDH

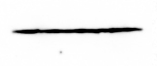

GAPDH

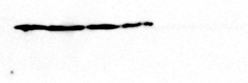

PC3 HSP40 day2

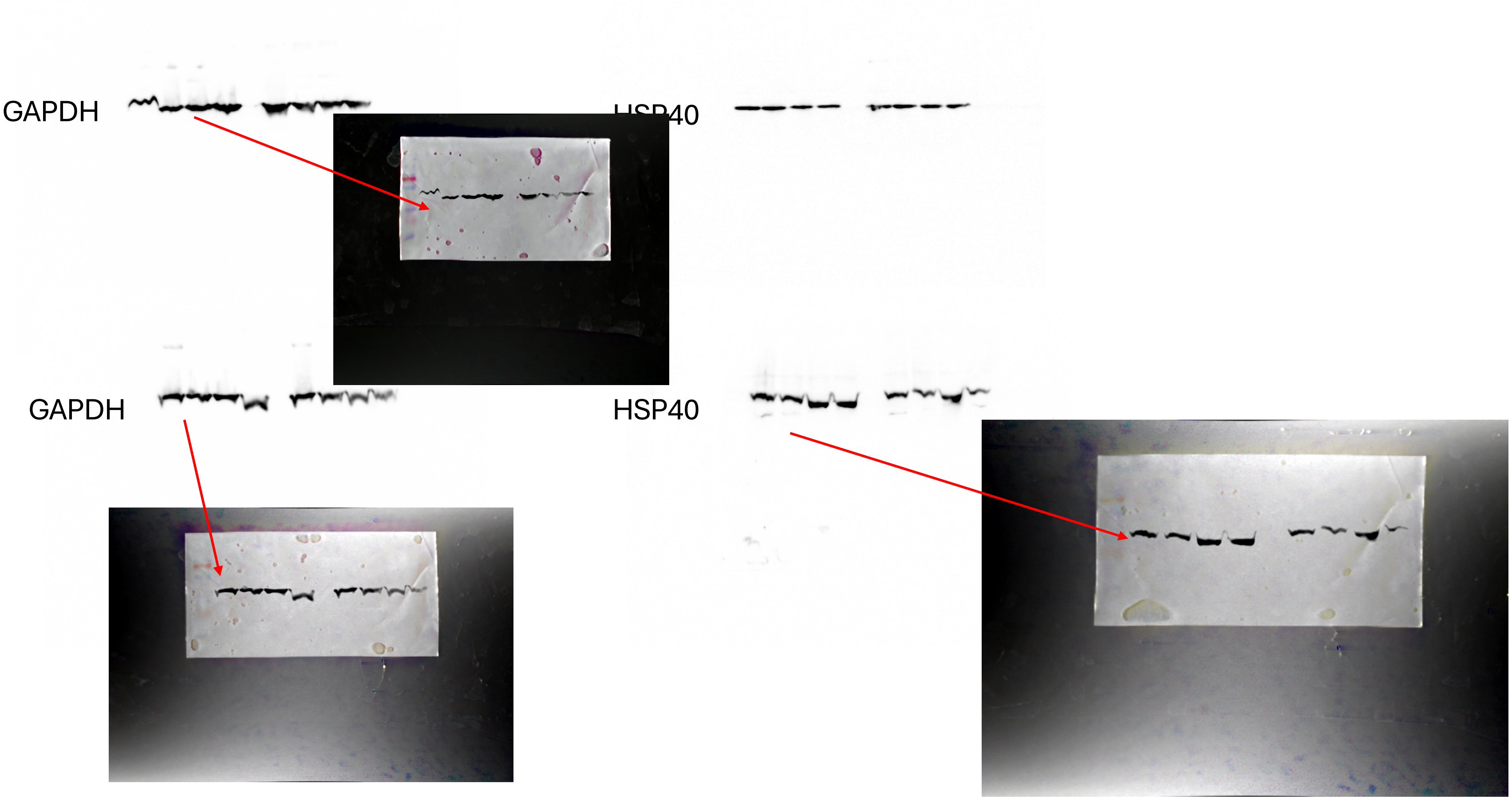

PC3 HSP40 day3

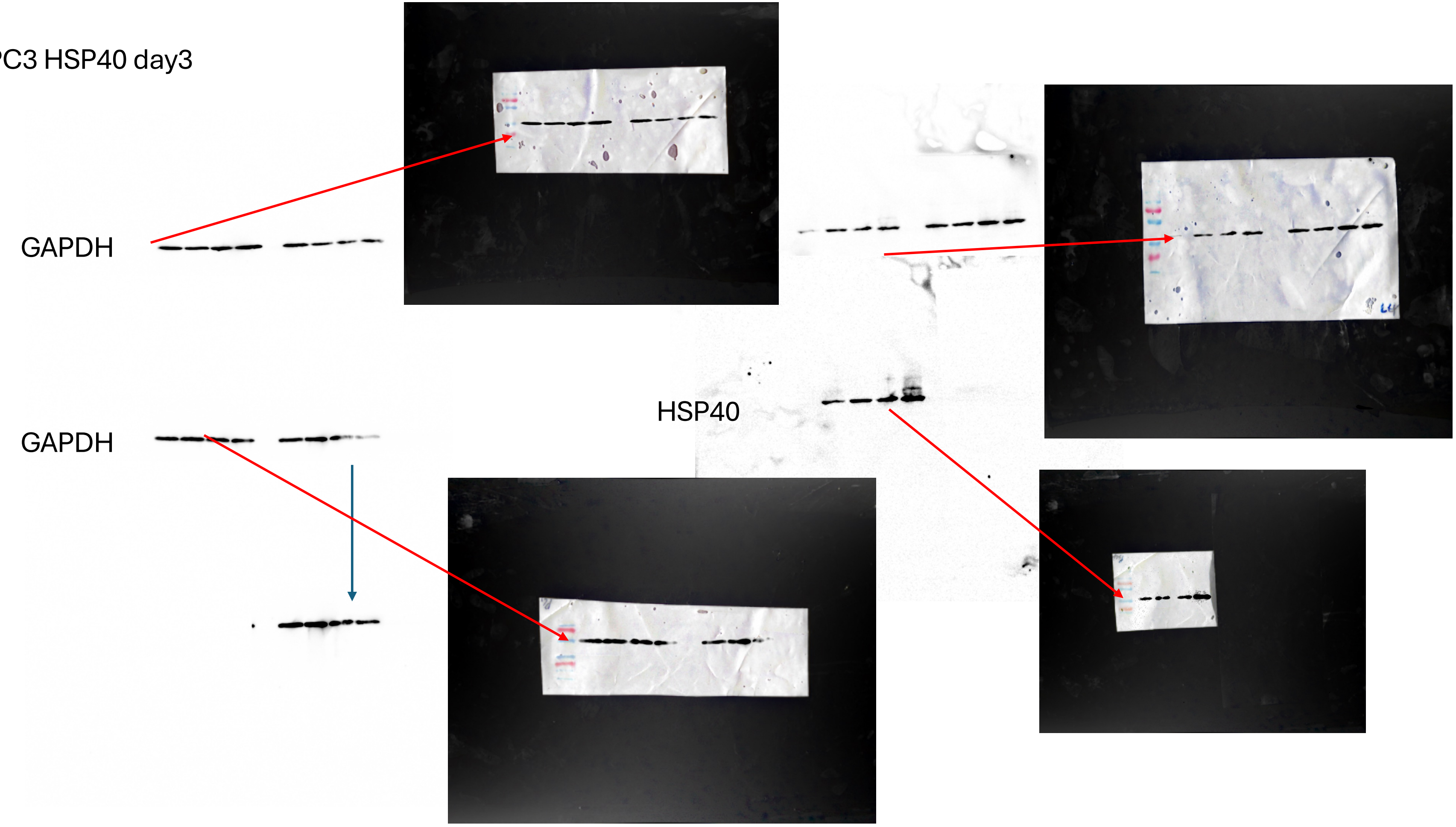

PC3 HSP70 day1

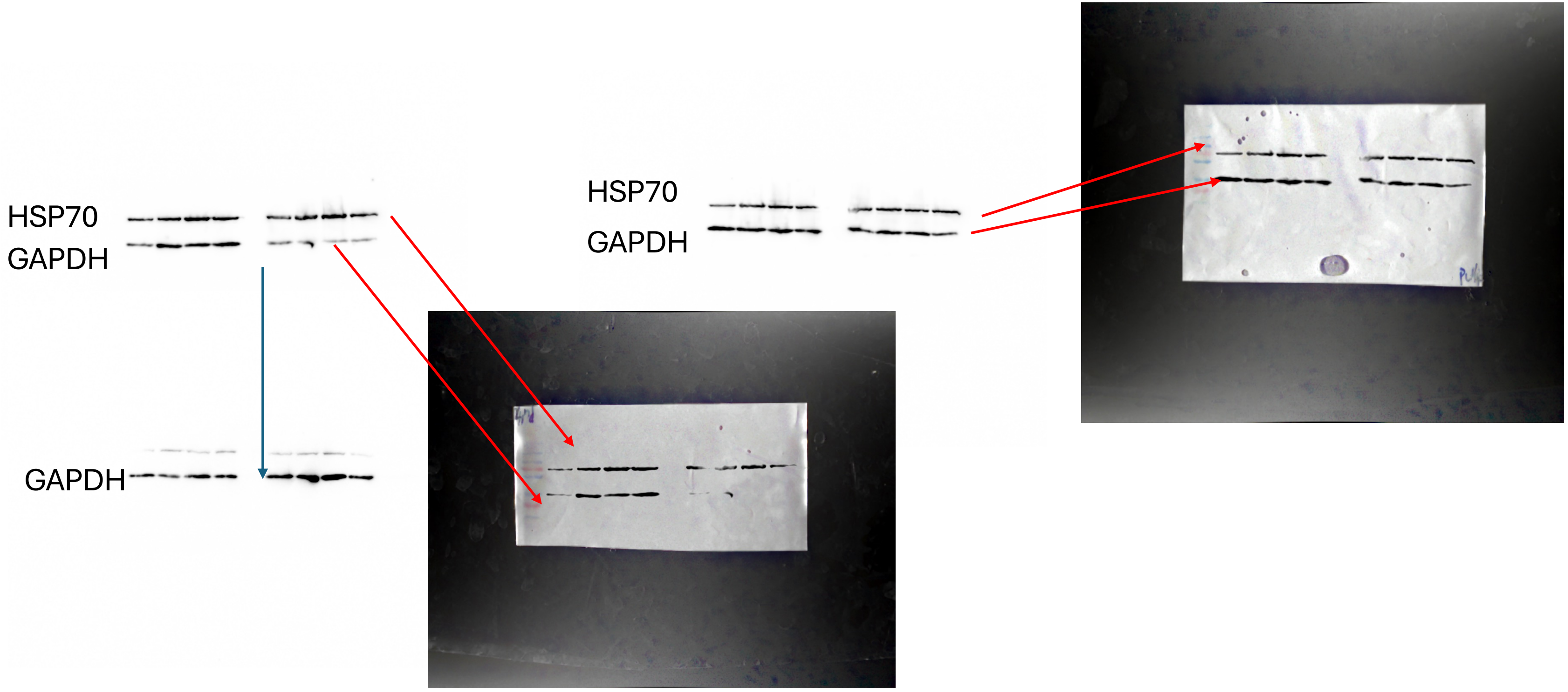

PC3 HSP70 day2

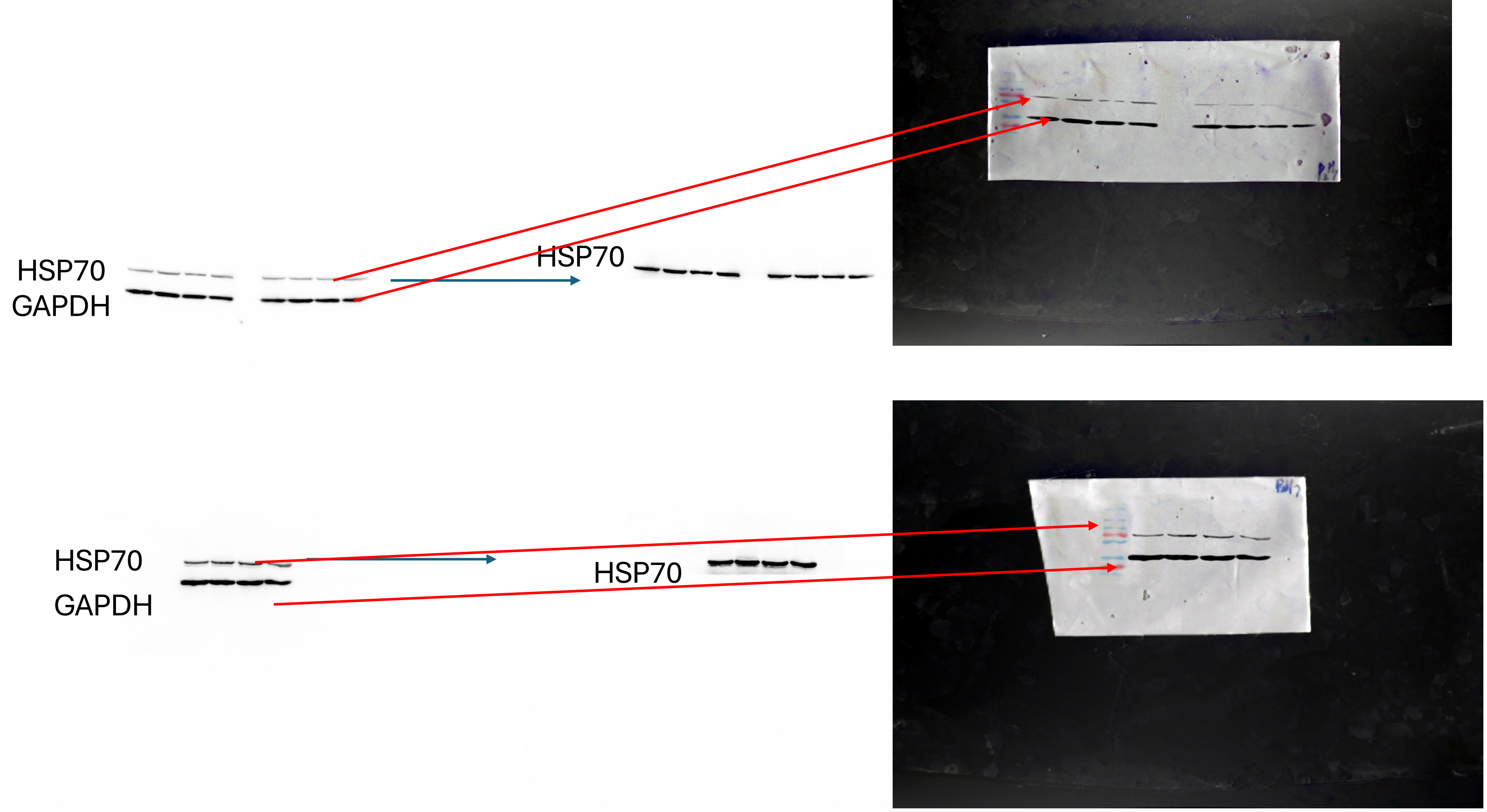

PC3 HSP70 day3

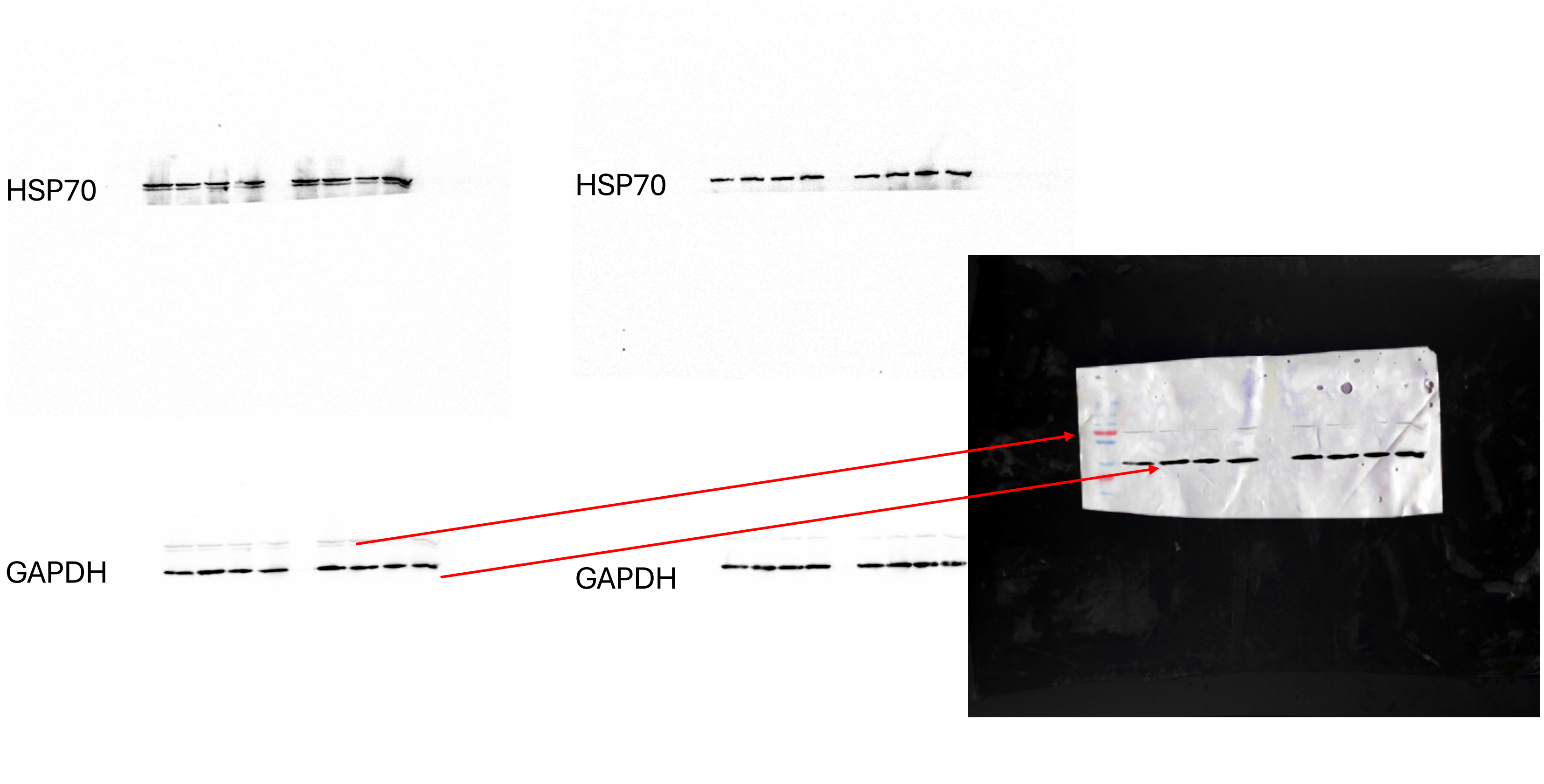

PC3 HSP90a day1

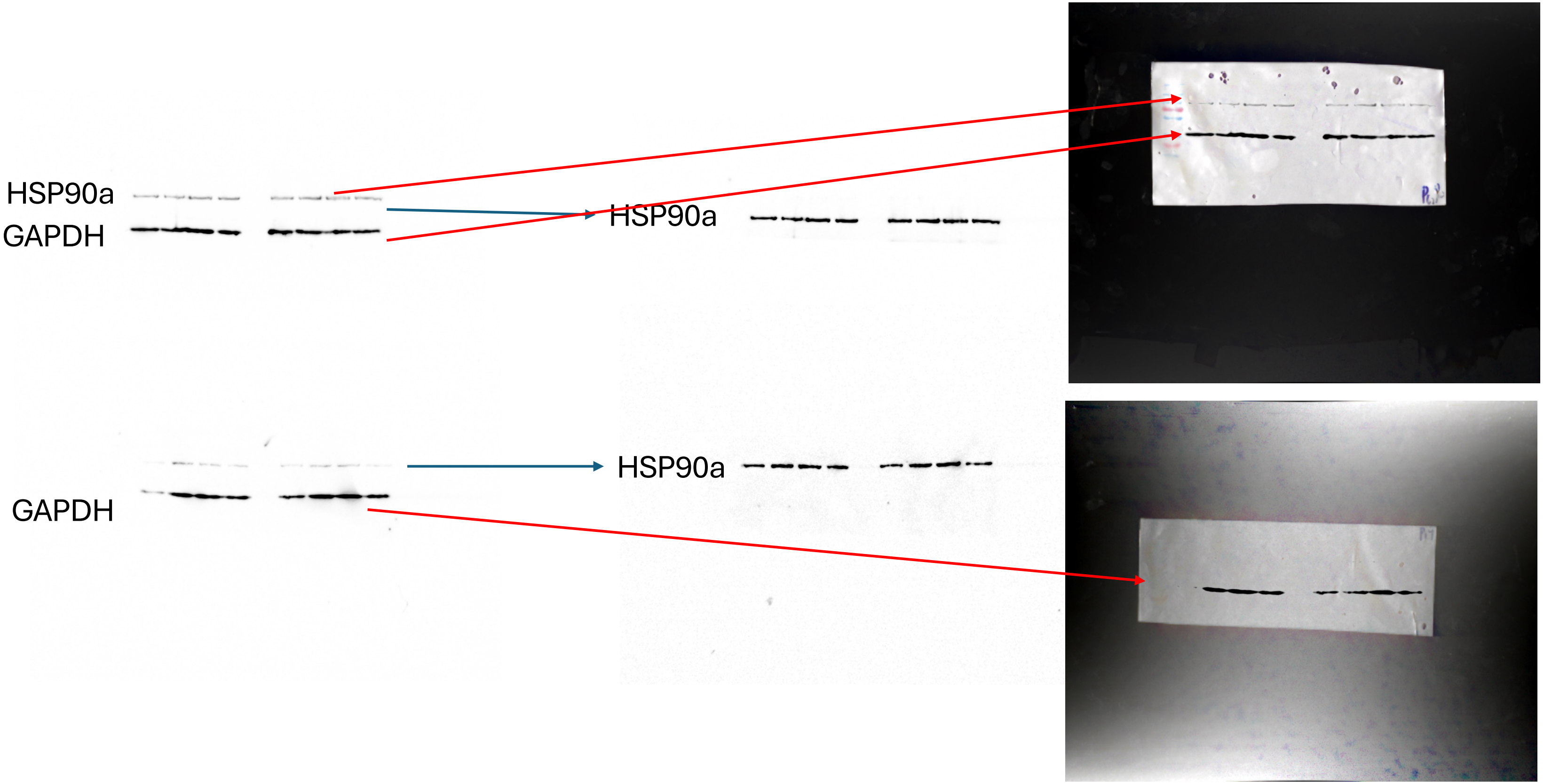

PC3 HSP90a day2

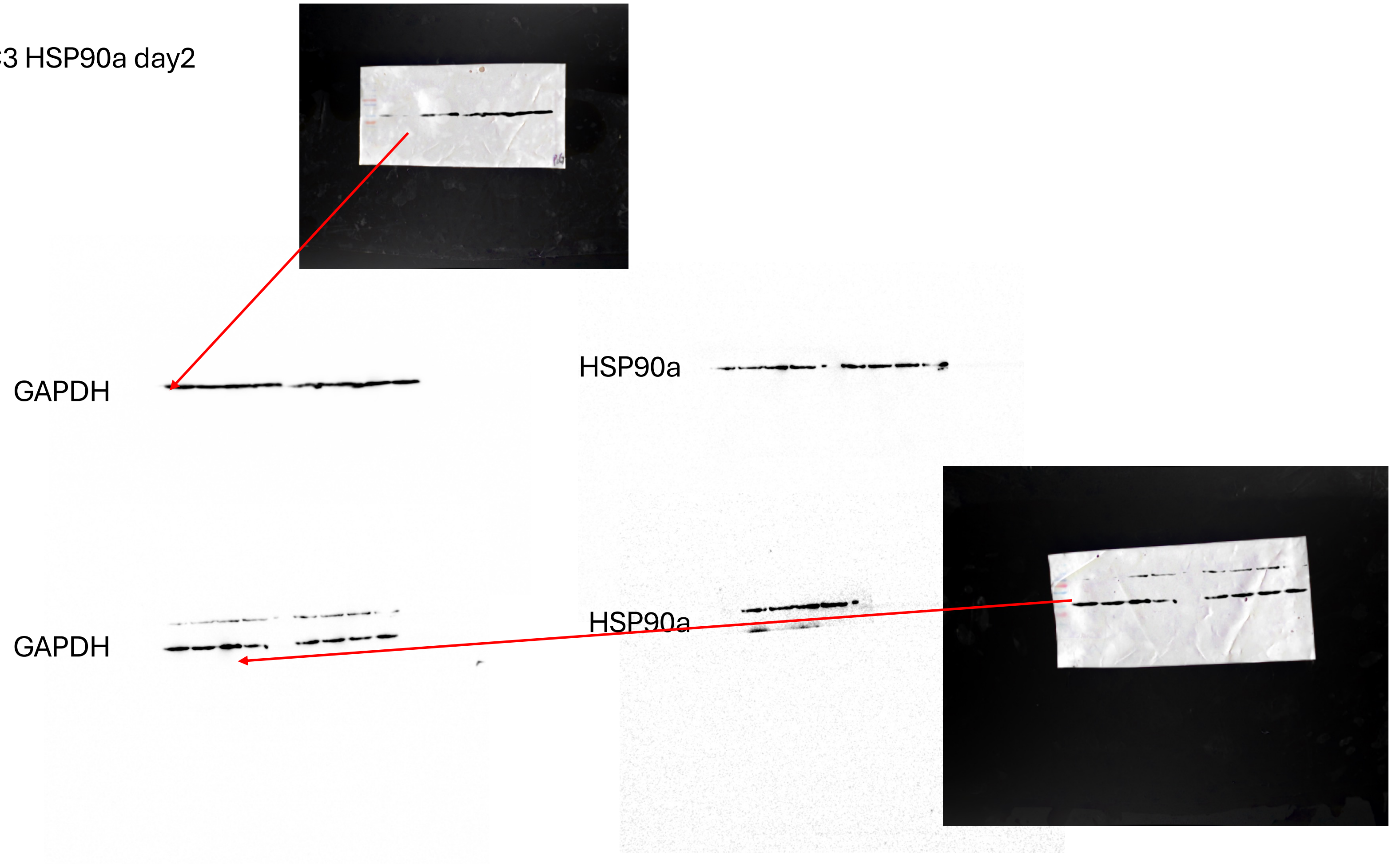

PC3 HSP90a day3

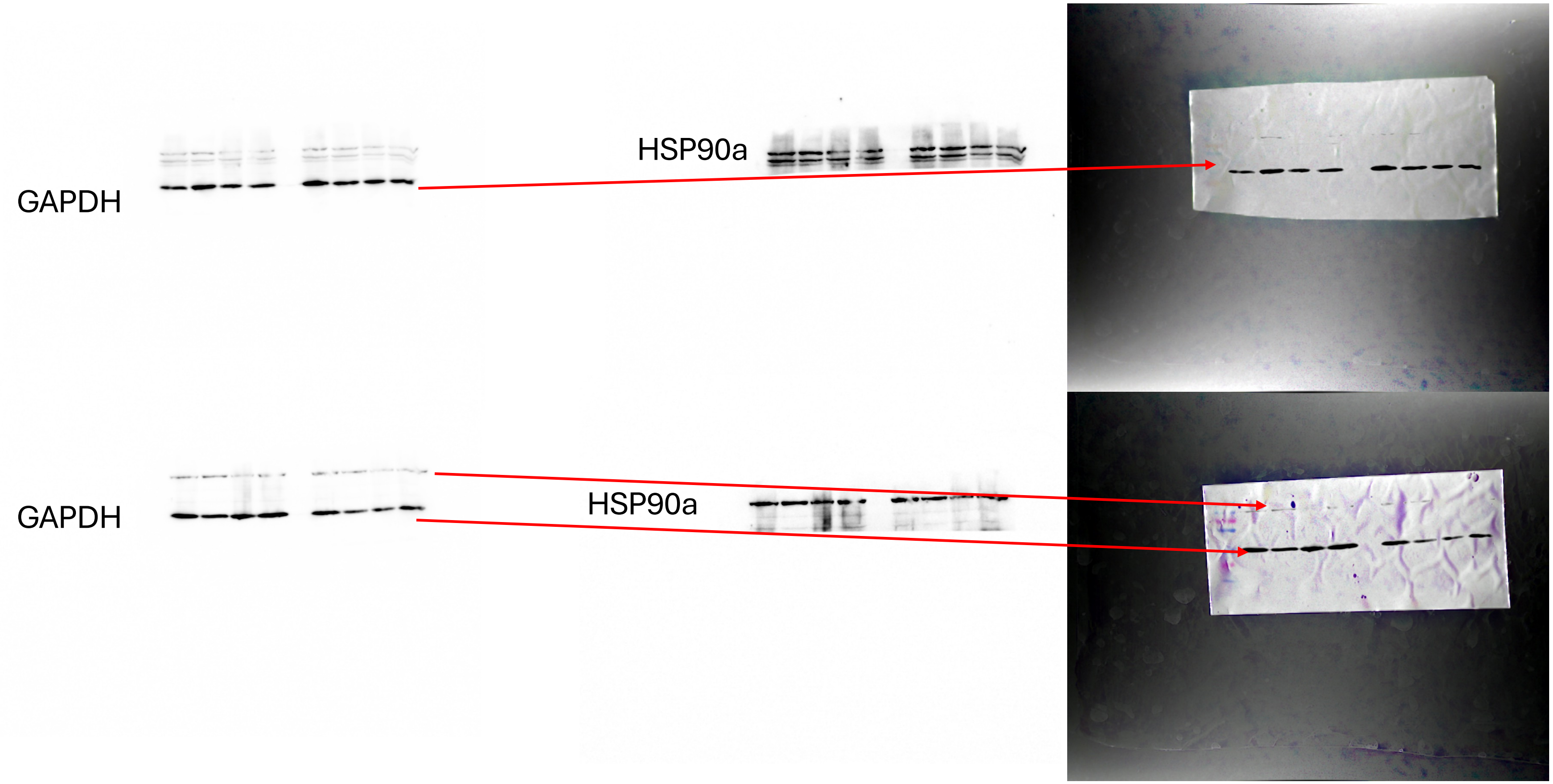

PC3 HSP90b day1

GAPDH

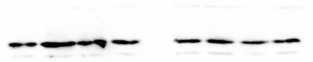

GAPDH

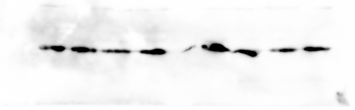

HSP90b

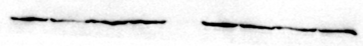

HSP90b

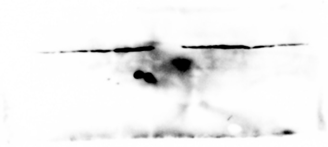

PC3 HSP90b day2

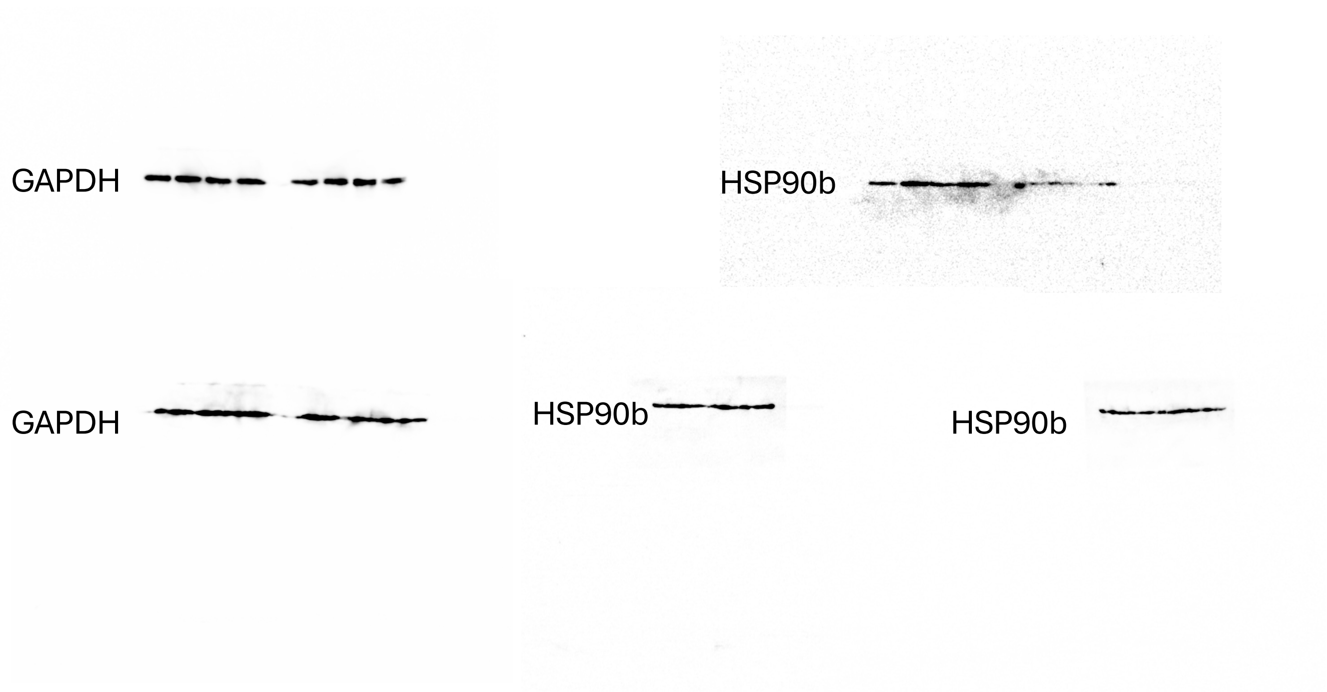

PC3 HSP90b day3

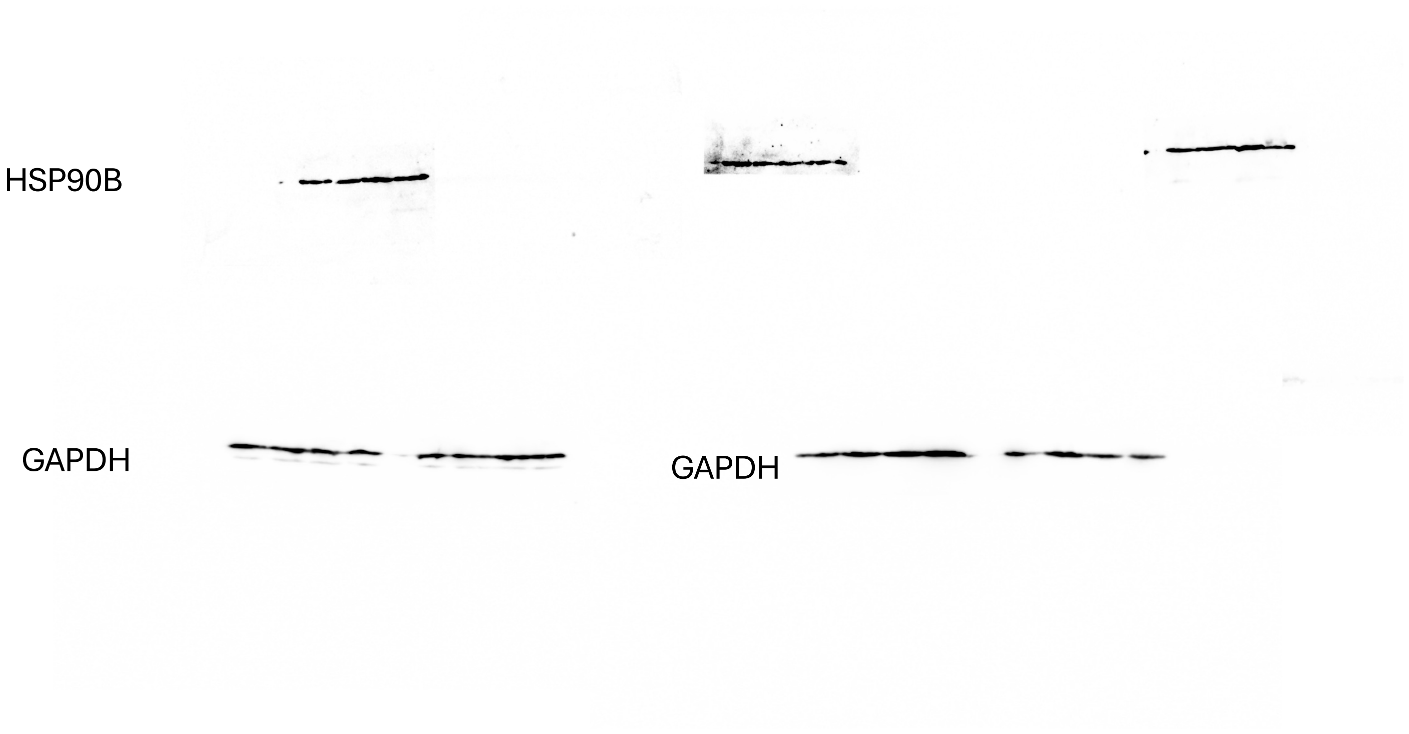

Supplement: Supplementary file 1 [file cancers-17-03517-s001.zip › cancers-3776093-supplementary/RAW BLOTS PC-3.pdf]
