# Supplementary material for: Non-Invasive Physical Plasma as an Oncological Therapy Option: Modulation of Cancer Cell Growth, Motility, and Metabolism Without Induction of Cancer Resistance Factors
Source: Cancers (Basel). 2025 Oct 31;17(21):3517. doi: 10.3390/cancers17213517 (PMC12607350; doi:10.3390/cancers17213517)

SKOV 3 HSP27 day1

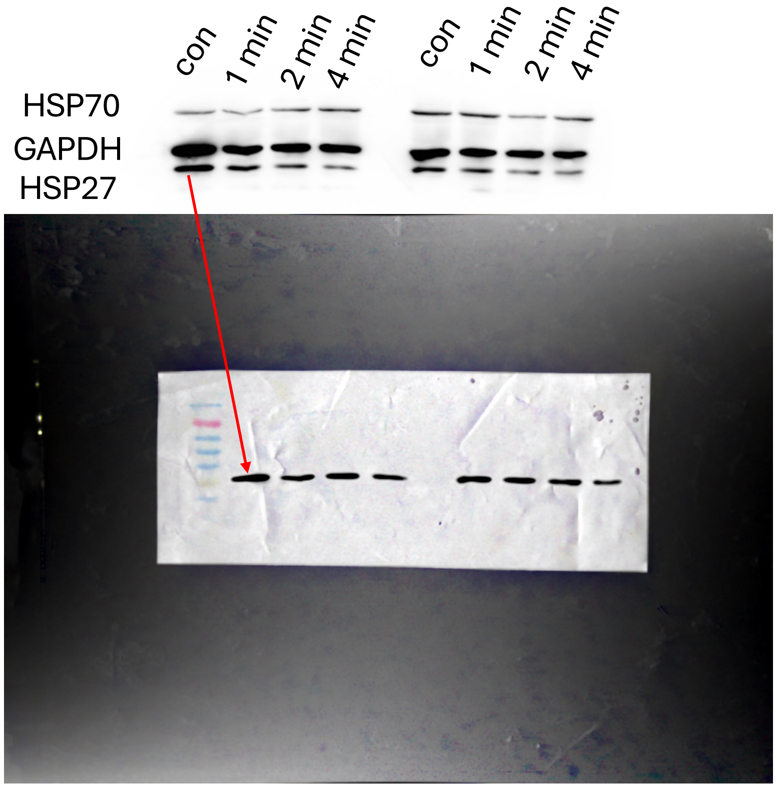

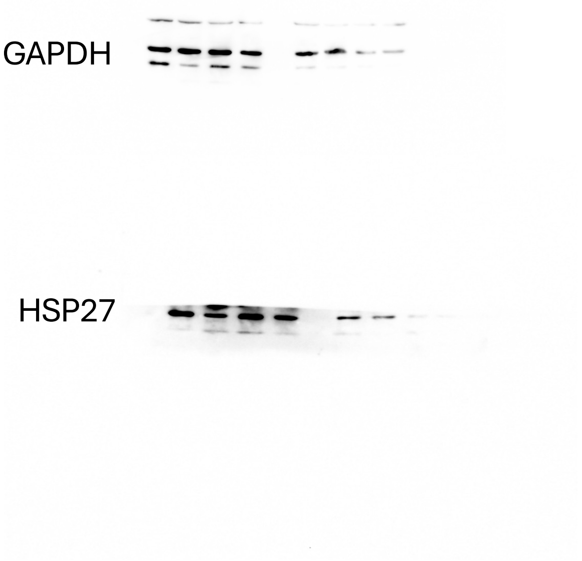

SKOV 3 HSP27 day2

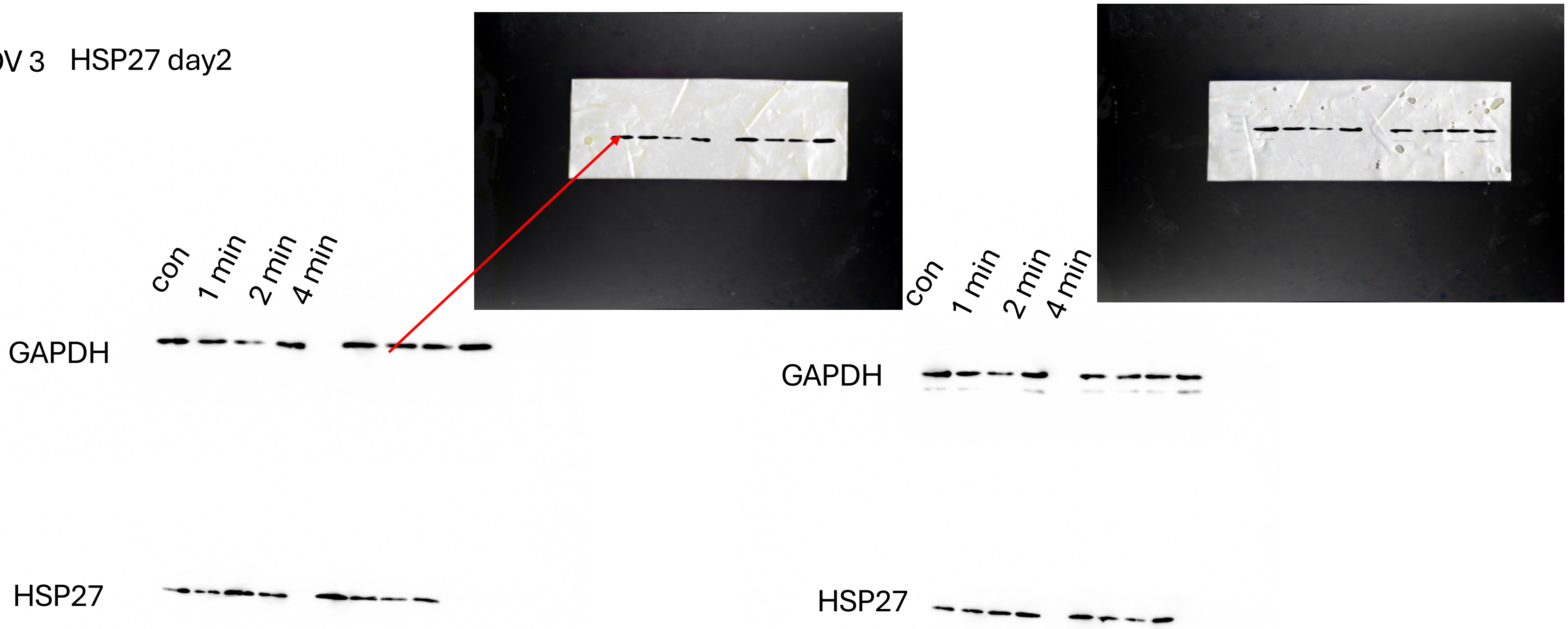

SKOV 3 HSP27 day 3

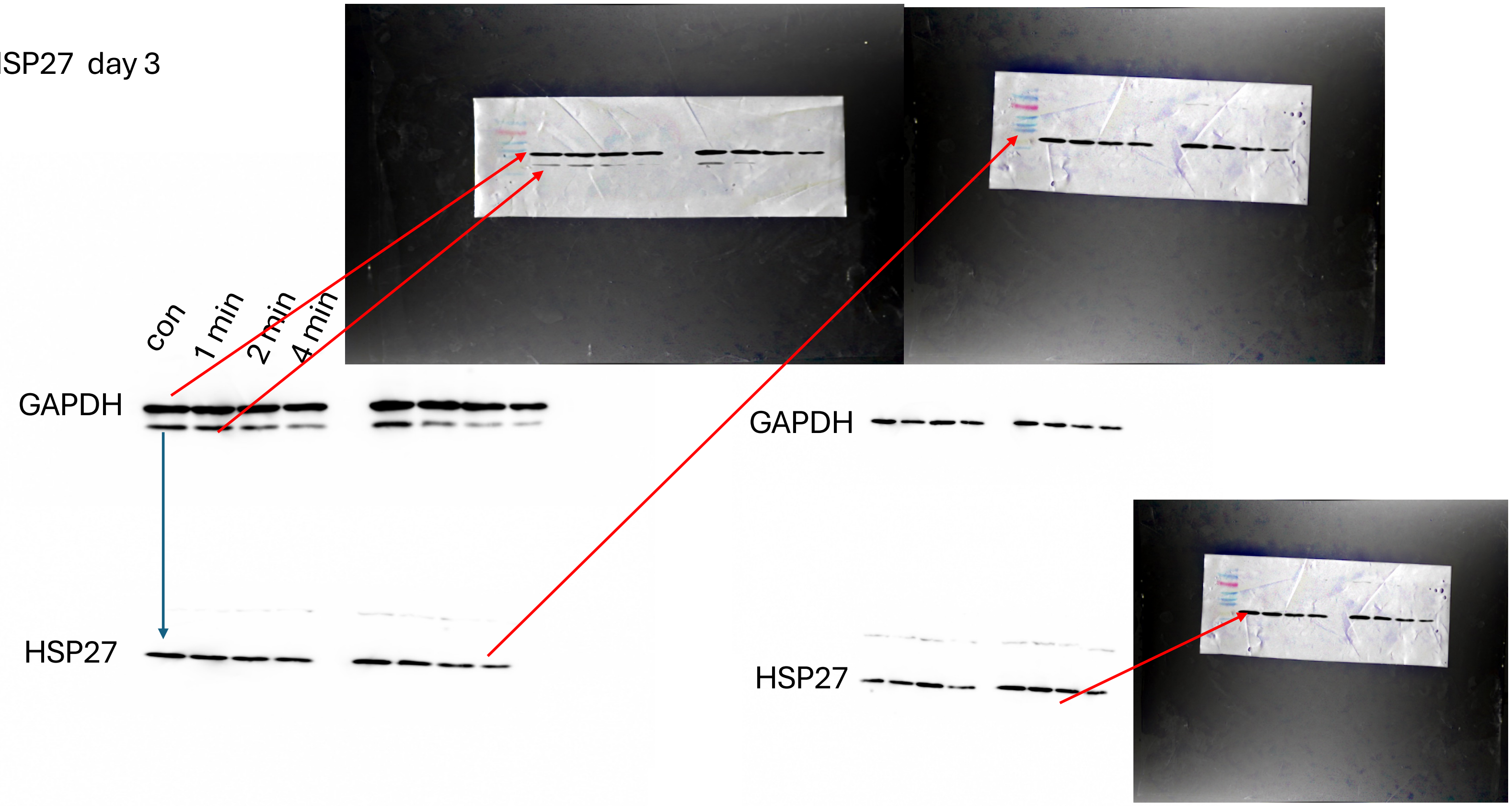

SKOV3 HSP40 day1

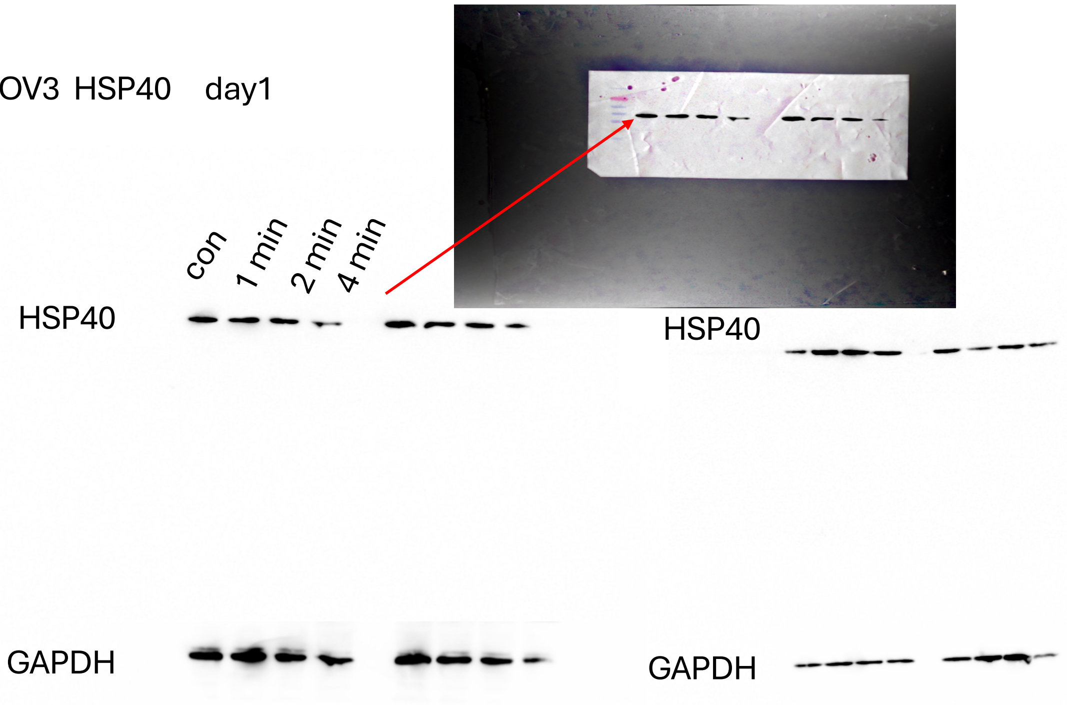

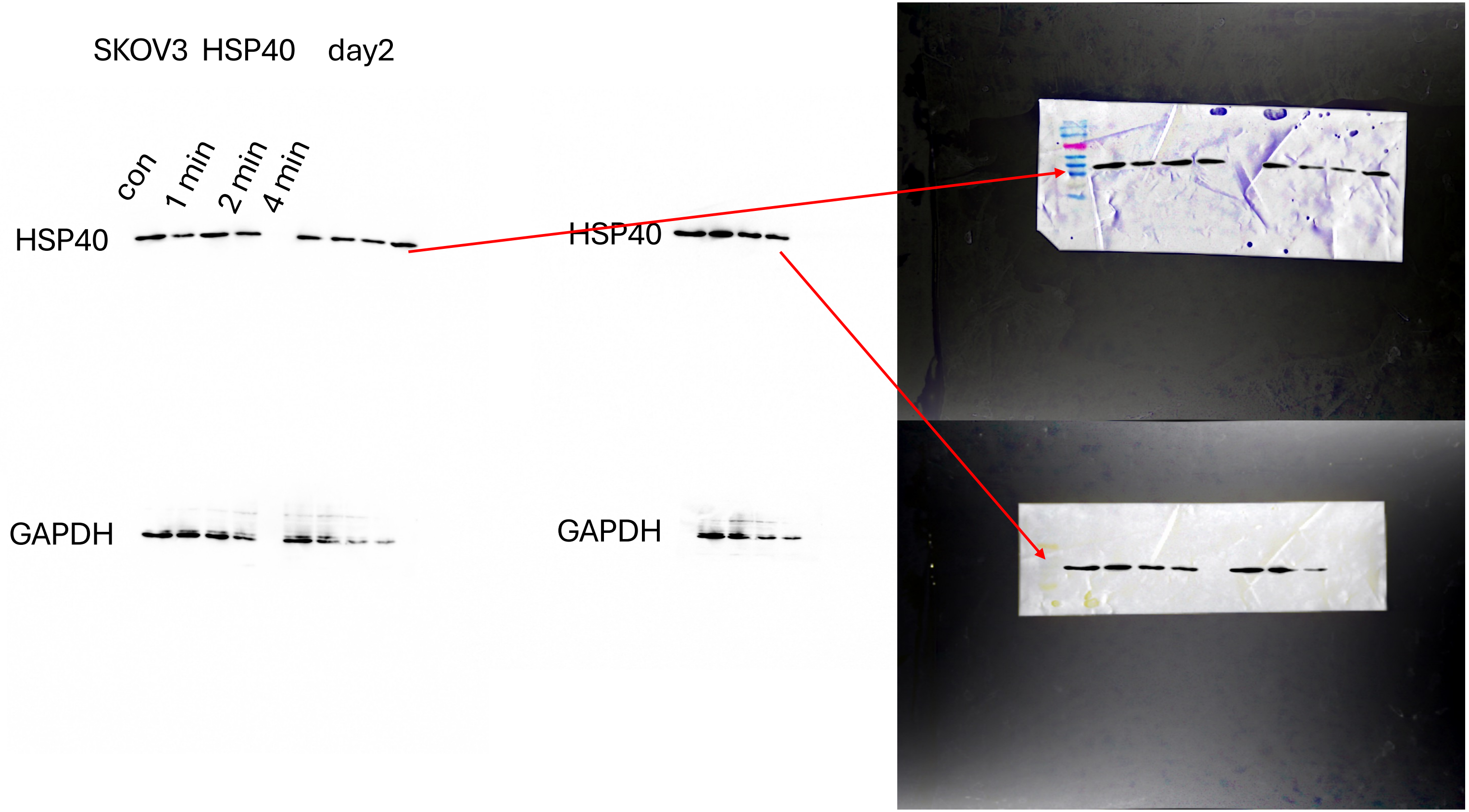
SKOV3 HSP40 day2
con
1 min
2 min
4 min
HSP40
GAPDH
HSP40
GAPDH

SKOV3 HSP40 day3

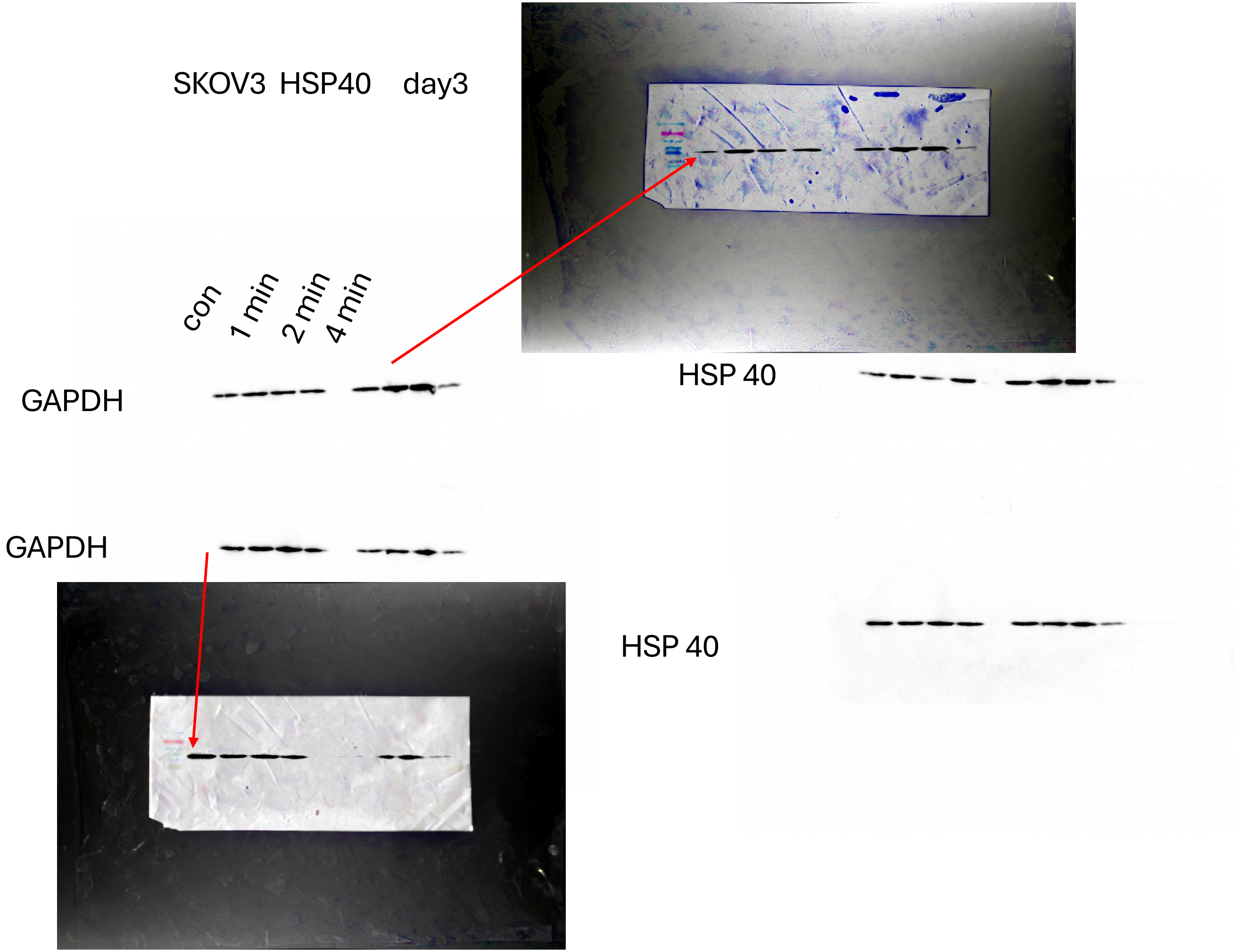

SKOV3 HSP70   day1

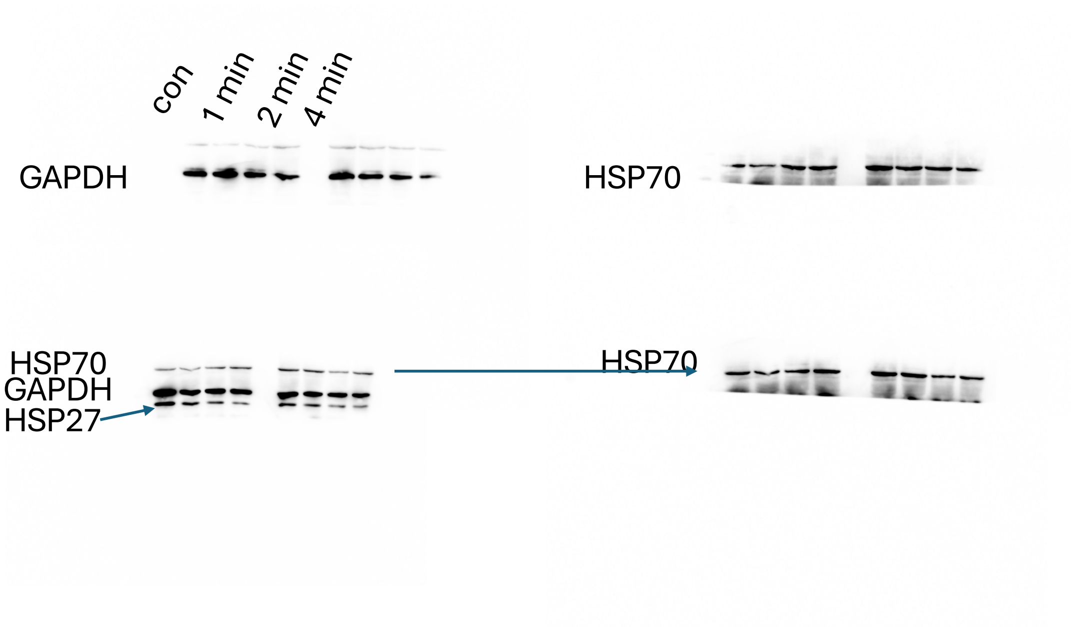

SKOV3 HSP70 day2

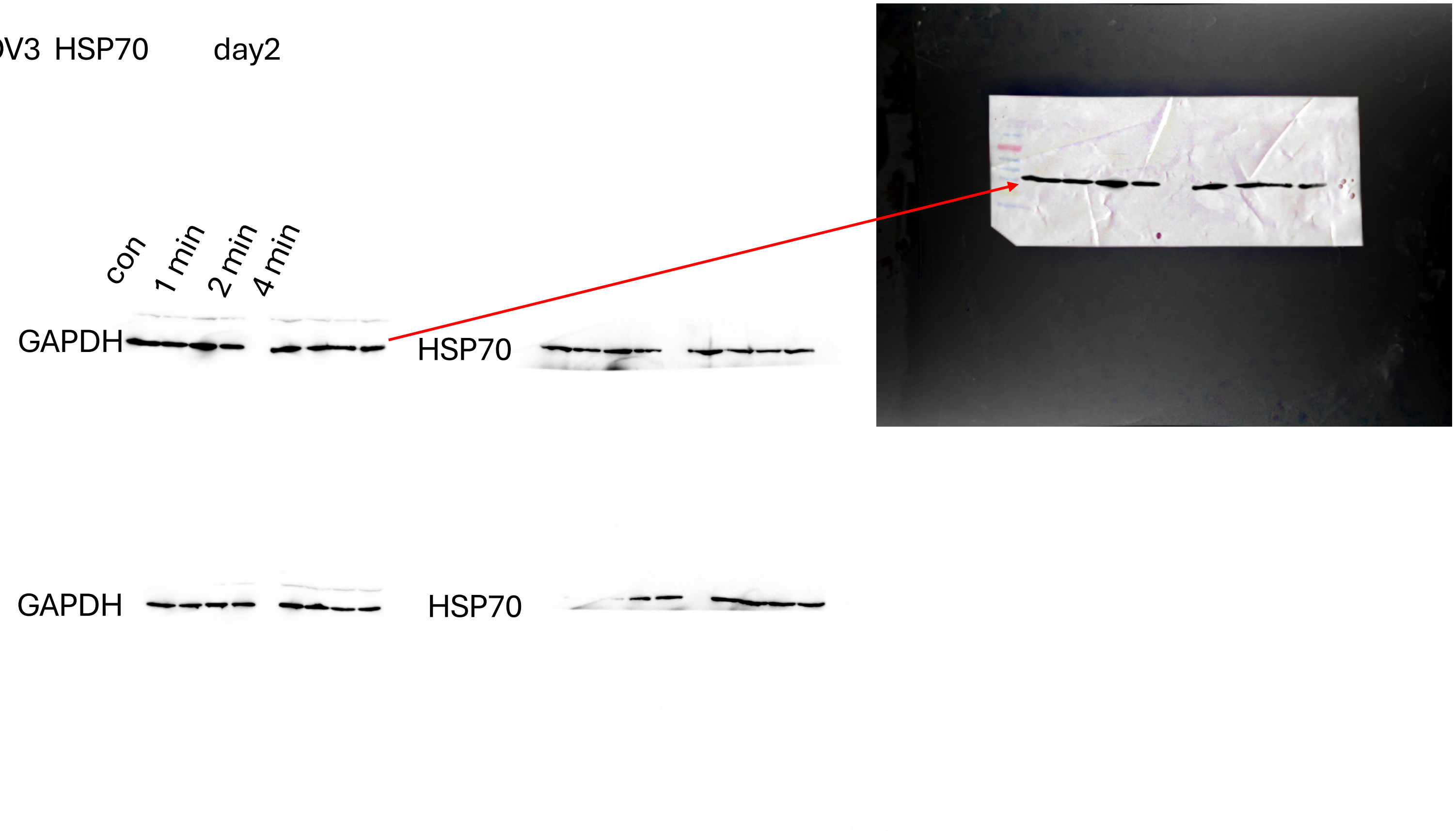

SKOV3 HSP70 day3

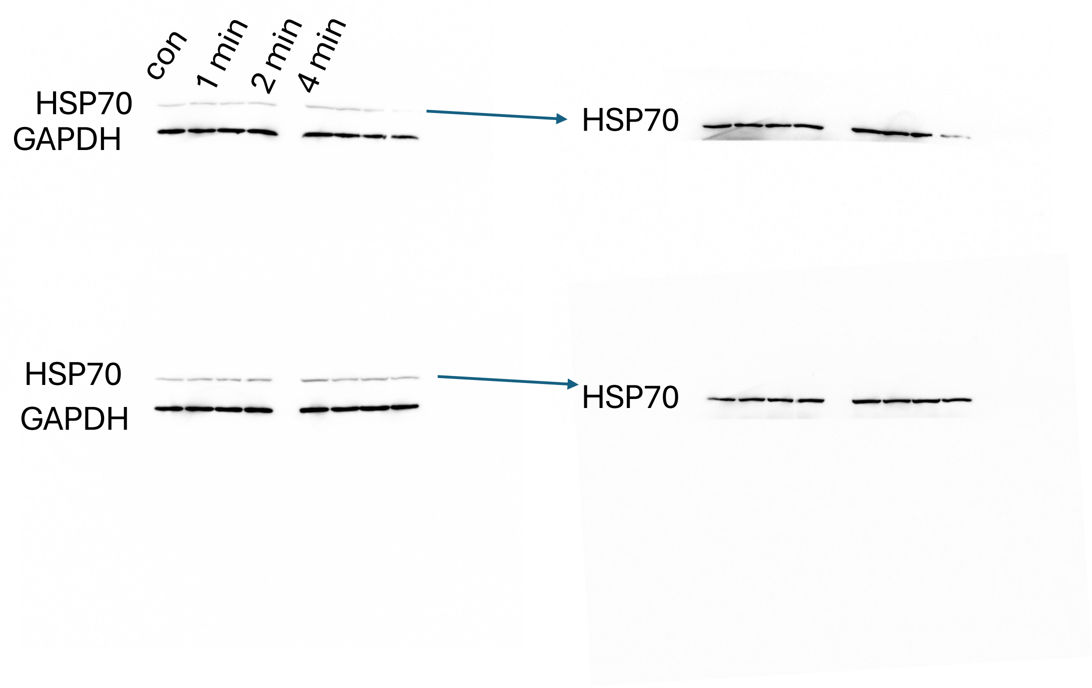

SKOV3 HSP90a day1

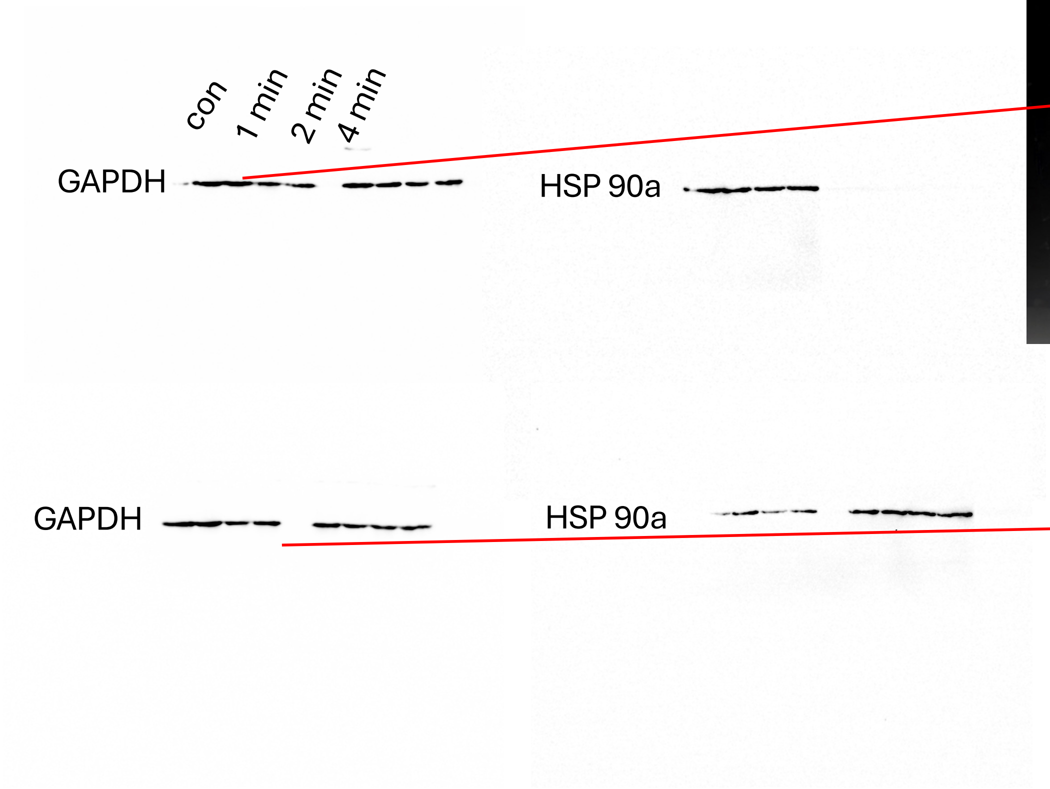

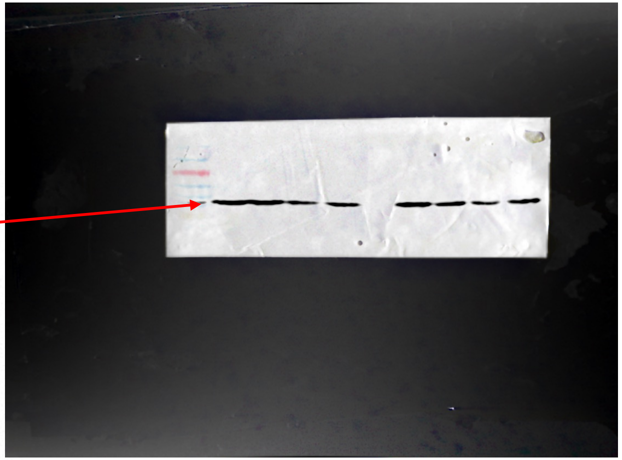

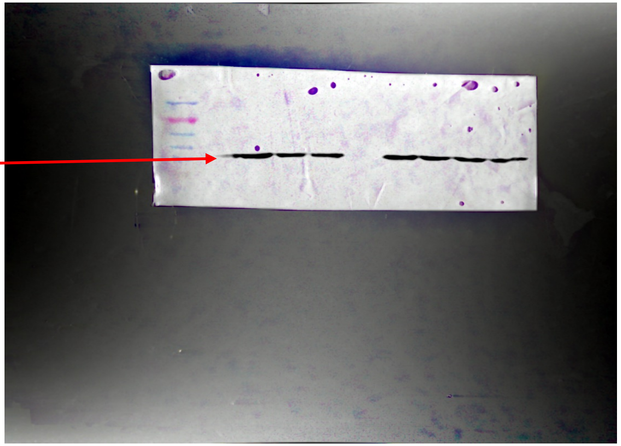

SKOV3 HSP90a day2

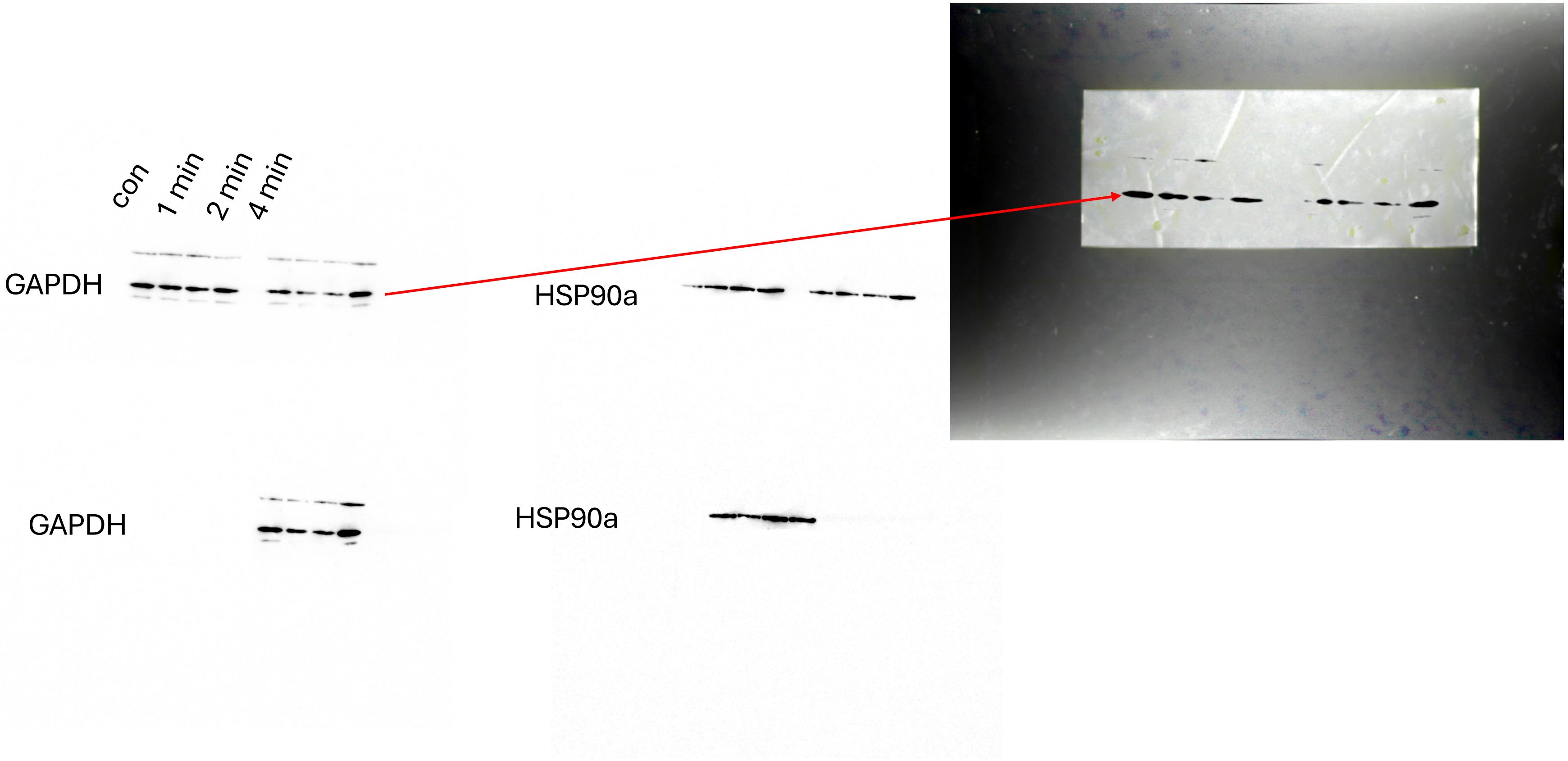

SKOV3 HSP90a day3

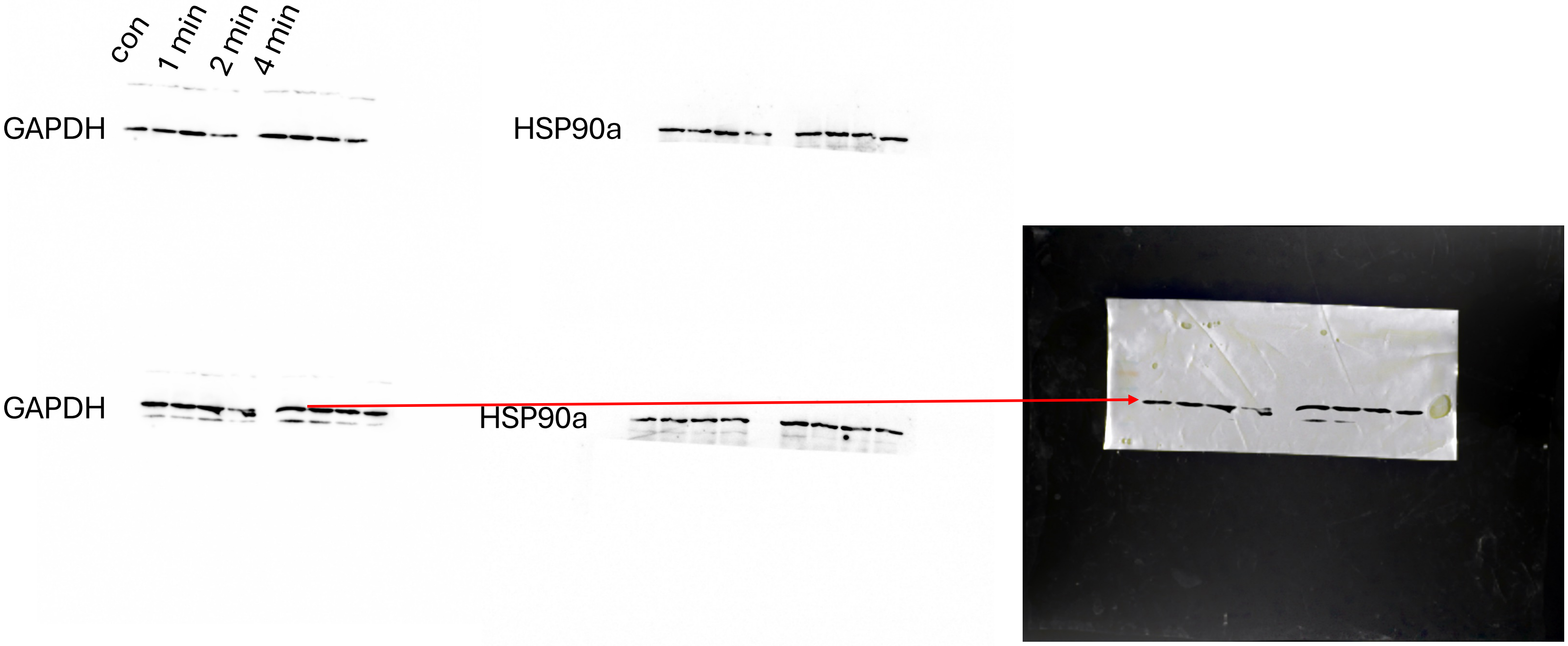

SKOV3 HSP90b day1

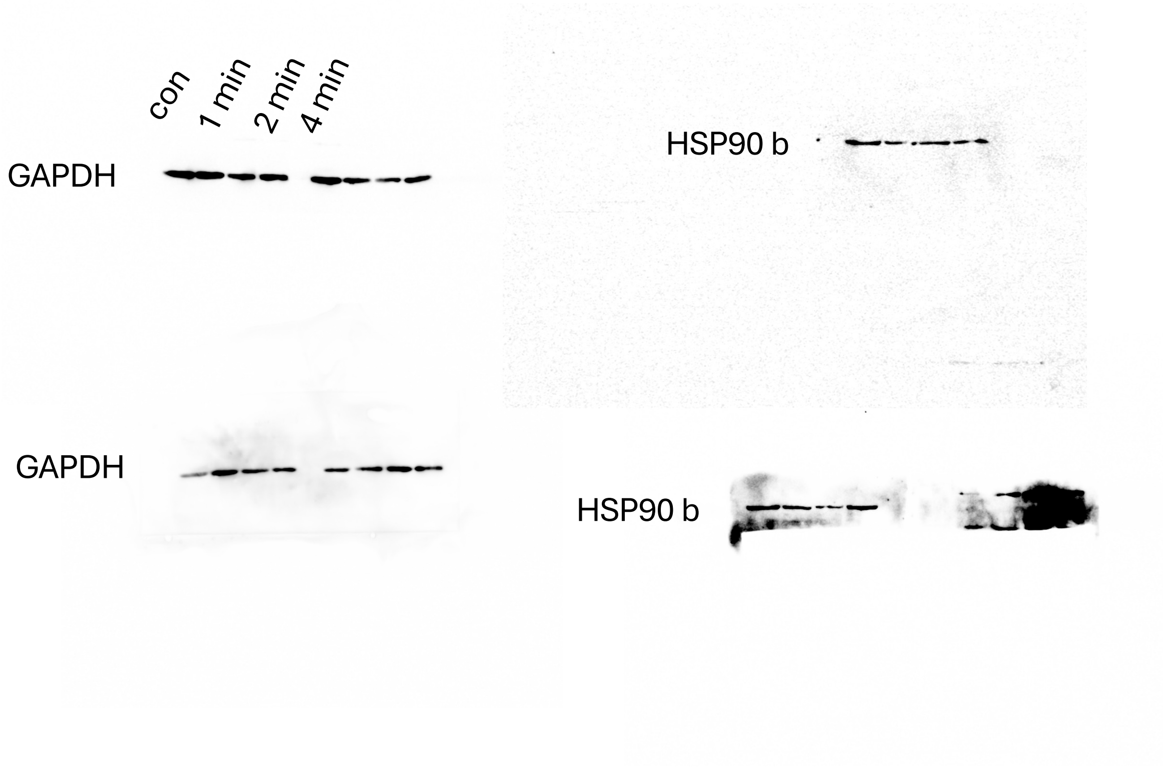

SKOV3 HSP90b day2

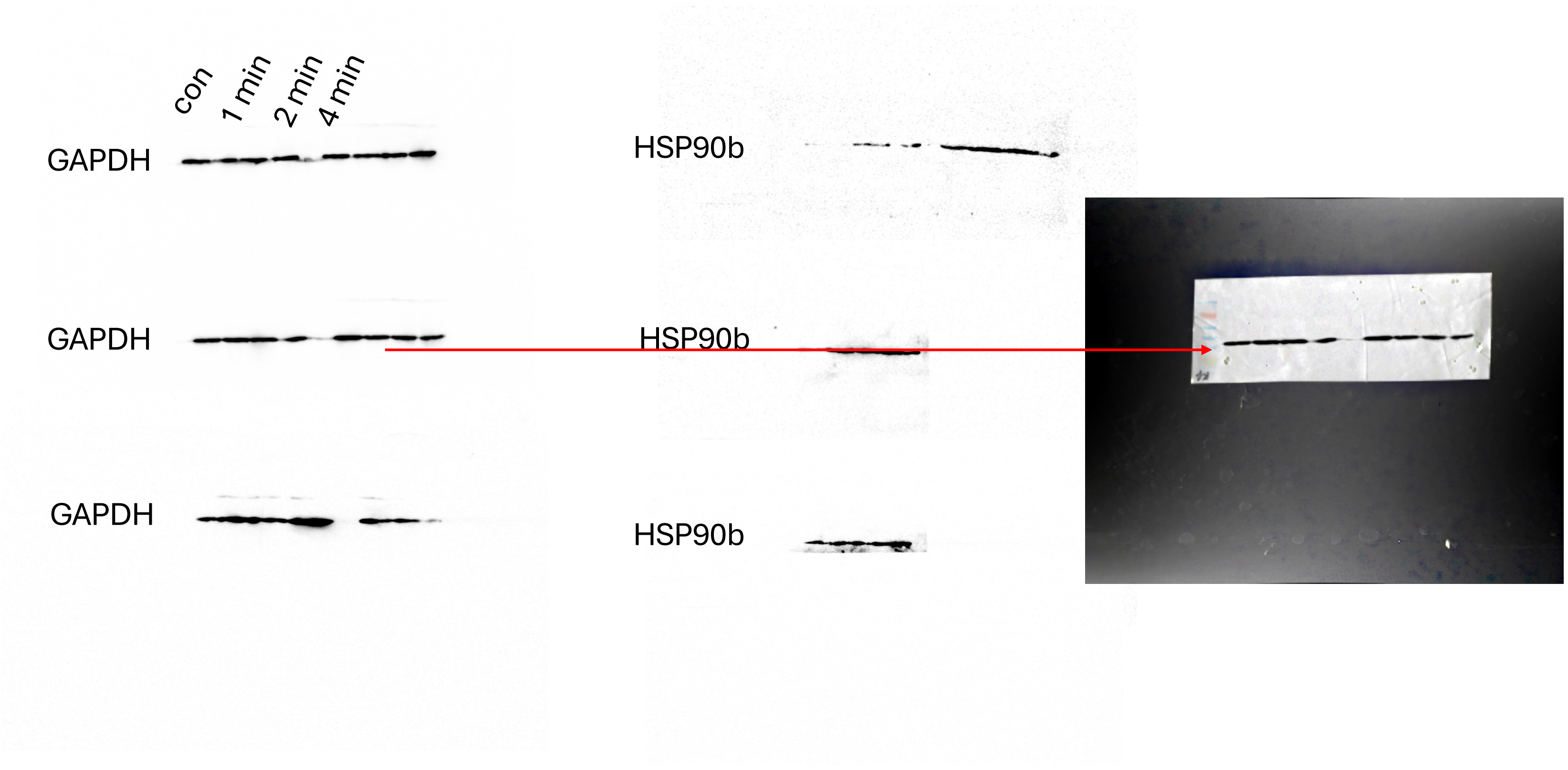

SKOV3 HSP90b day3

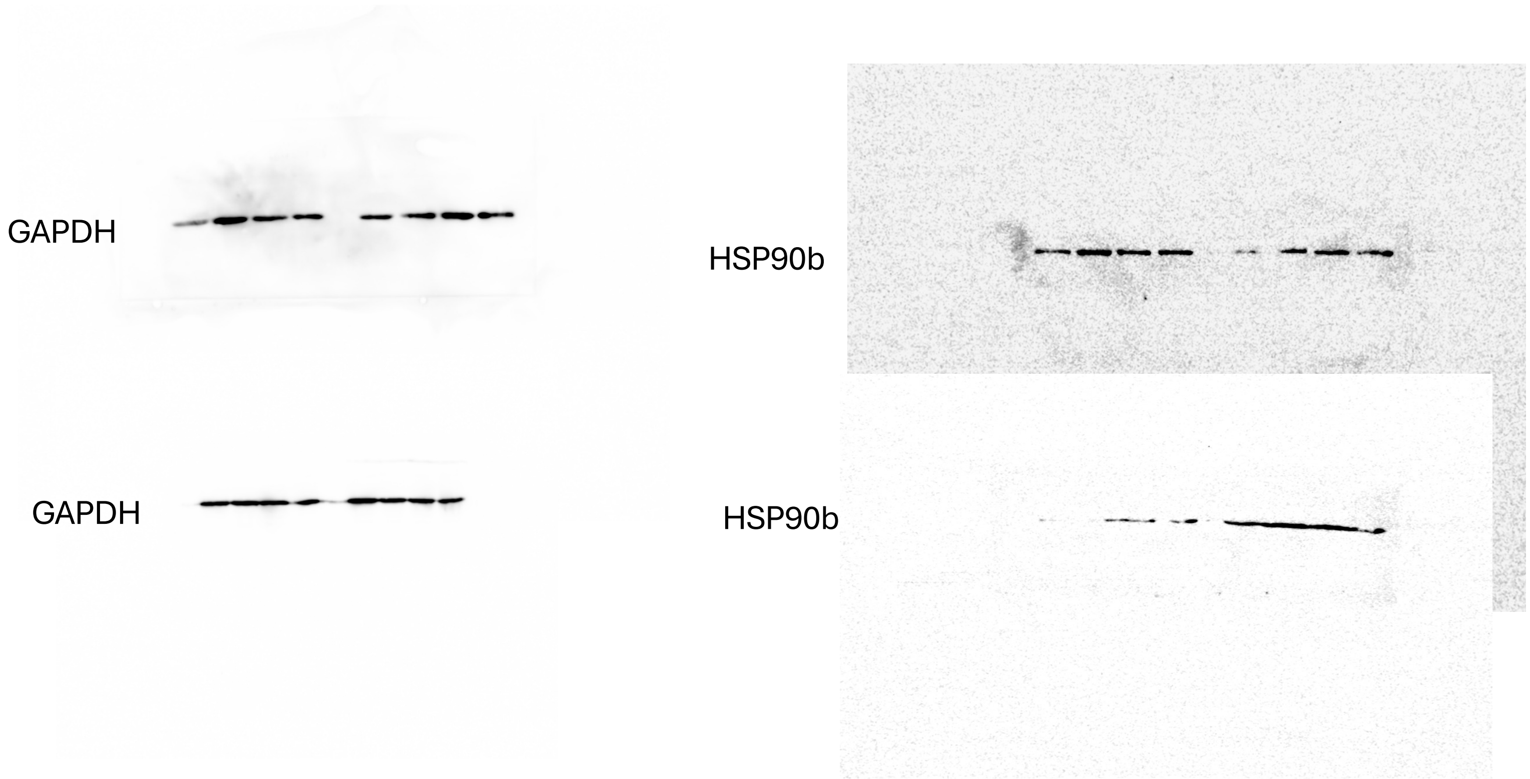

Supplement: Supplementary file 1 [file cancers-17-03517-s001.zip › cancers-3776093-supplementary/RAW BLOTS SKOV-3.pdf]
